# Intelligent multimodal-energy-driven piezoelectric antibacterial platforms: From structural control to system-level diagnosis

**DOI:** 10.1016/j.mtbio.2026.103299

**Published:** 2026-05-29

**Authors:** Jiaqi Liu, Jun Li, Huiyan Sun, Jungmok Seo, Yanmei Zhang, Yunlong Yu

**Affiliations:** a College of Life Science, Dalian Minzu University, Economical and Technological Development Zone, Dalian, 116600, China; b State Key Laboratory of Catalysis, Dalian Institute of Chemical Physics, Chinese Academy of Science, P. O. Box 110, Dalian, 116023, China; c School of Electrical and Electronic Engineering, Yonsei University, Seoul, 03722, Republic of Korea; d Institute of Burn Research, Southwest Hospital, Third Military Medical University (Army Medical University), Chongqing, 400038, China

**Keywords:** Antibiotic resistance, Piezoelectric catalytic technology, Intelligent antibacterial platform, Rational material design, Spatiotemporal control, Adaptive diagnosis and treatment system

## Abstract

Piezocatalysis offers a non-antibiotic, physical-chemical approach to address the challenge of bacterial infection resistance, but its reliance on single energy sources and empirical design limits its potential. This review focuses on intelligent piezoelectric antibacterial platforms powered by multimodal energy and enhanced by artificial intelligence, systematically summarizing the latest research progress in the field. First, based on an in-depth analysis of the limitations of traditional antibacterial strategies, the evolutionary trajectory of piezoelectric technology from single-energy to multimodal synergistic driving is clarified. Second, we systematically explain the piezocatalytic antibacterial mechanism and reveal its synergistic enhancement with other antibacterial components under multiphysical fields, highlighting their comprehensive advantages in improving antibacterial efficacy and achieving precise spatiotemporal control. Furthermore, the synergistic regulatory effects of key structural parameters on the piezoelectric properties and antibacterial activity of materials are thoroughly analyzed. Additionally, typical application cases of such intelligent platforms in cutting-edge scenarios, such as smart wound management, functional anti-infection implant coatings, and precise intervention for localized infections in the lungs and oral cavity, are systematically reviewed. Finally, critical challenges related to material stability, biosafety, and scalable production are discussed, and future research directions empowered by AI are prospected. This review establishes an interdisciplinary theoretical framework and extensible technical pathway for constructing adaptive piezoelectric antibacterial platforms.

## Graphical abstract

To overcome antibiotic resistance and the limitations of conventional therapies, this review explores how integrating multimodal energy-driven piezocatalysis with coordination chemistry and AI can create intelligent antibacterial platforms with precise spatiotemporal control for next-generation infection treatment.

**Figure:**
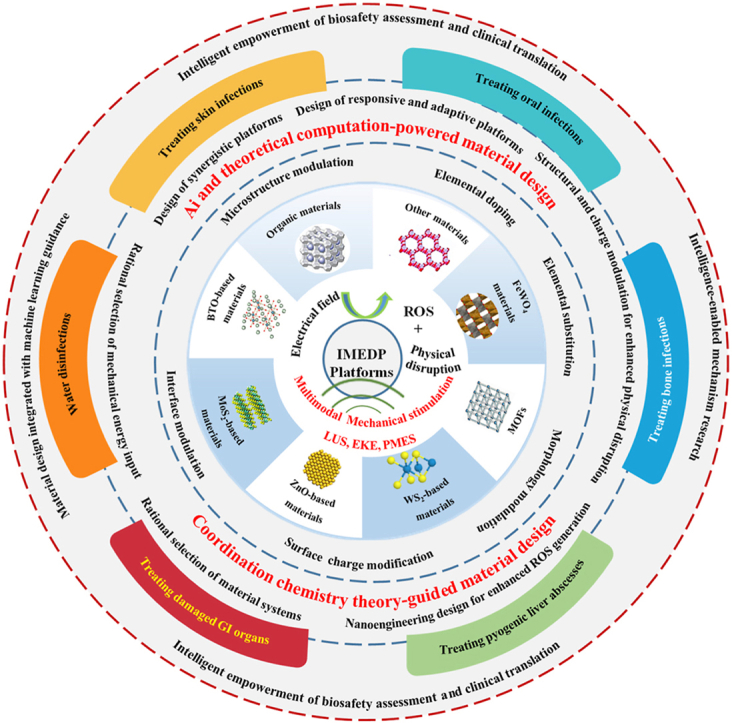

## 1 Introduction

Against the escalating global public health crisis, the rapid dissemination of antimicrobial resistance (AMR) has been recognized by the World Health Organization as one of the top ten most severe public health threats worldwide [1]. Projections have demonstrated that AMR is projected to claim 10 million lives annually worldwide by 2050 in the absence of effective intervention measures [2]. In this grim context, biofilm-associated infections stand out as a pressing clinical issue, as biofilms are formed in over 80% of bacterial infectious cases [3,4]. The unique physical barrier structure and quorum sensing system of biofilms enhance bacterial tolerance to antibiotics by 10 to 1000 times, making refractory issues such as chronic wound infections and medical device-associated infections difficult to eradicate [5]. Current clinical practice faces a triple dilemma: the pipeline for novel antibiotic development is increasingly depleted, resistance rates to existing drugs continue to rise, and traditional therapies exhibit limited penetration capabilities against biofilms [6,7]. Concurrently, various passive antimicrobial materials suffer from inherent shortcomings, including short activity duration, a tendency to induce local toxicity, and a lack of intelligent responsiveness. These conventional strategies generally lack spatiotemporal precision and environmental adaptability, rendering them insufficient to meet the demands of the precision medicine era for intelligent antibacterial diagnostic and therapeutic systems.

To overcome the limitations of existing antibacterial strategies, piezoelectric catalytic antibacterial technology has emerged. This technology establishes a non-antibiotic-dependent “physical-chemical” synergistic antibacterial mechanism by converting external stimuli such as mechanical energy into localized electric fields and reactive oxygen species (ROS), thereby opening a novel therapeutic pathway [8,9]. Piezoelectric catalytic materials can construct “endogenously driven, intelligently responsive” antibacterial systems. By harnessing the mechanical energy ubiquitously present in living organisms, such as ultrasound, blood flow pulsation, and respiratory movements, these systems enable in situ precise intervention for deep-seated infections [[10], [11], [12]]. The core mechanism lies in the charge separation and formation of a strong built-in electric field within the material under mechanical stimulation, which directly catalyzes the generation of highly oxidative ROS from surrounding water molecules and oxygen [13].

Compared to traditional antibacterial methods, this system offers distinct advantages. On one hand, it can activate materials within deep tissues using high tissue-penetrating ultrasound or endogenous physiological mechanical energy, effectively overcoming the tissue penetration limitations inherent in methods like traditional photodynamic therapy [14]. On the other hand, its antibacterial activity is triggered on-demand only under mechanical stimulation, demonstrating excellent spatiotemporal controllability and significantly reducing nonspecific damage to surrounding healthy tissues [15,16]. Furthermore, this technology can be integrated with physiological energy sources, such as blood flow and muscle contractions, to construct self-sustaining antibacterial systems, providing continuous and reliable antibacterial protection for long-term implantable medical devices.

However, the current development of piezoelectric antibacterial technology still faces numerous challenges. First-generation systems primarily rely on single-energy input modes, resulting in limited energy conversion efficiency. Material design often employs empirical trial-and-error approaches, lacking systematic theoretical guidance [17,18]. The systems’ insufficient intelligence also hinders precise regulation of therapeutic processes. Notably, recent interdisciplinary convergence has injected new momentum into this field. The integration of theoretical computation and artificial intelligence technologies, grounded in coordination chemistry principles, has shifted material design from empirical screening to rational construction [19,20]. Moreover, by leveraging multimodal energy synergy strategies, piezoelectric catalysis significantly enhances energy utilization efficiency, while the integration of artificial intelligence technology provides robust support for the development of intelligent diagnostic and therapeutic systems. Correspondingly, these cutting-edge advancements are collectively propelling piezoelectric antibacterial platforms toward more intelligent and precise development [21].

While current review studies on the mechanisms and typical applications of piezoelectric catalysis have achieved certain results, there remains a noticeable gap in the theoretical framework for systematic intelligence-driven development [[22], [23], [24], [25], [26], [27], [28], [29], [30], [31], [32], [33]]. A complete theoretical framework for the transition from “responsive materials” to “self-adaptive intelligent systems” has yet to be established. Significant research gaps persist, particularly in critical areas such as dynamic response mechanisms to the infectious microenvironment, real-time biological information feedback systems, and precise on-demand therapeutic strategies [14,34,35].

**Scheme 1:**
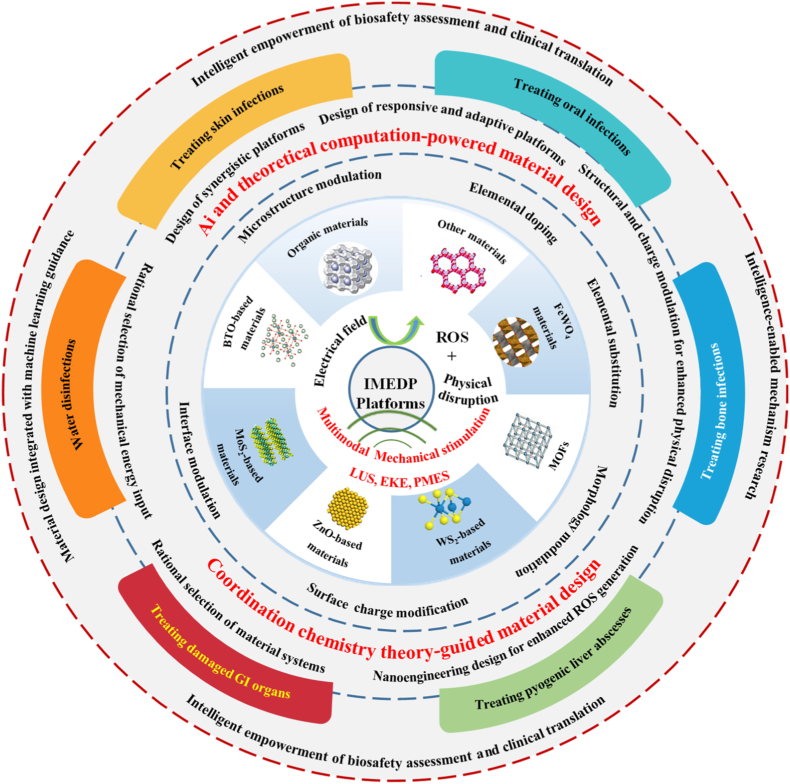
Principles and applications of intelligent multimodal-energy-driven piezoelectric platforms (IMEDPP): from structural control to system-level diagnosis for advanced antibacterial applications.

In this context, as outlined in Scheme 1, this review aims to systematically consolidate research progress in this field and expand its theoretical boundaries. It not only explores rational material design based on theoretical computation and artificial intelligence but also examines the integrated design and functional fusion pathways from “piezoelectric materials” to “AI-enabled intelligent diagnostic-therapeutic systems”. Furthermore, the review provides an in-depth analysis of the overall regulatory mechanisms of piezoelectric systems on the infectious microenvironment. By establishing a comprehensive analytical framework spanning mechanisms of action, design principles, and clinical application evaluation, this review aspires to offer systematic theoretical guidance and a technical roadmap for the development of next-generation intelligent platforms targeting biofilm-associated and deep-seated infections, thereby advancing the field toward a higher level of intelligence and precision. Furthermore, we clarify four often-confused terms in this review to avoid ambiguous usage and better reflect the different levels of system complexity. Self-powered systems refer to devices that harvest environmental energy (e.g., mechanical vibration, body movement) without requiring an external power supply, but they lack the ability to respond actively to changing environmental conditions [36]. Adaptive systems, in contrast, are capable of adjusting their behavior or properties based on predefined microenvironmental thresholds, such as pH changes caused by bacterial infection, but they do not necessarily incorporate feedback loops [37]. Smart systems integrate self-powering and adaptive functions with sensing-actuation feedback loops, enabling real-time response to environmental cues [38]. Intelligent systems represent the highest level of complexity, incorporating machine learning and closed-loop control to learn, predict, and autonomously optimize their performance across different disease contexts [39]. These definitions, together with the representative examples provided, clearly delineate the hierarchical progression from simpler energy-harvesting devices to fully autonomous intelligent systems.

## 2 Mechanism of piezocatalysis-driven antibacterial systems

Piezocatalytic antibacterial therapy is a physico-chemical synergistic strategy that utilizes piezoelectric materials to generate piezoelectric potential under mechanical stress (such as ultrasound, vibration, or fluid shear forces), thereby catalyzing the production of ROS or directly disrupting bacterial structures.

### 2.1 Mechanism of piezocatalysis

**Scheme 2:**
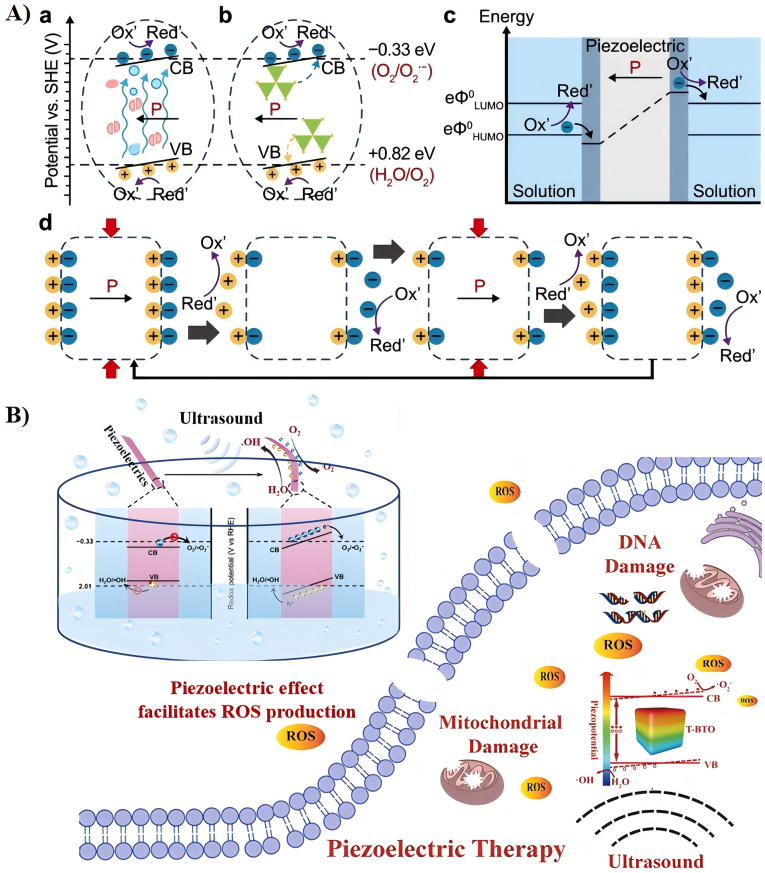
A) Schematic illustration of free carriers in piezoelectric materials generated (a) via mechanical excitation and (b) through intrinsic defects. VB: Valence Band, CB: Conduction Band; (c) Schematic diagram of the electrocatalysis-like mechanism. LUMO: Lowest Unoccupied Molecular Orbital, HUMO: Highest Occupied Molecular Orbital; (d) Dynamic screening phenomenon induced by the piezoelectric effect. P: Polarization field, Ox’: Oxidant, Red’: Reductant [42]. Copyright 2025, Elsvier; B) Schematic illustration of the ultrasound (US)-induced piezoelectric polarization of a piezoelectric material, which may drive ROS-producing reactions on its surfaces. Reproduced with permission [43,44]. Copyright 2020, American Chemical Society.

Currently, three main theoretical frameworks have been proposed to explain the mechanism of piezocatalysis: energy band theory, electrocatalysis-like theory, and the screened charge effect [40,41]. Within energy band theory, the origin of free carriers in piezocatalysis remains debated, with two possible sources proposed: excited electron/hole pairs generated by mechanical energy excitation and intrinsic free charges in the piezocatalyst (Scheme 2A-(a-b)). In electrocatalysis-like theory, it is suggested that the piezopotential generated by the piezocatalyst can alter its band gap, contrasting with energy band theory where the piezopotential only tilts the conduction band/valence band of the catalyst while the band gap remains unchanged (Scheme 2A-c). The piezocatalytic reaction based on the screened charge effect mechanism (Scheme 2A-d) can be summarized as follows: (i) Under applied mechanical force, the piezocatalyst surface adsorbs screening charges from the surrounding medium. (ii) Upon removal of the force, the adsorbed screening charges are released from the surface due to the disappearance of the internal electric field. (iii) Given that the redox capability of the adsorbed screening charges is comparable to that of the conduction band/valence band edge sites in the piezocatalyst, redox reactions occur between the screening charges and certain substances in the surrounding medium (e.g., O_2_, H_2_O, persulfate molecules). (iv) When external stress is reapplied, polarization is reestablished in the piezocatalyst, and screening charges from the surrounding medium are once again adsorbed onto its surface, thereby initiating further redox reactions [42].

### 2.2 Piezocatalysis-driven antibacterial mechanism through ROS-induced oxidative stress

As shown in Scheme 2B, the piezocatalysis-mediated ROS generation involves three mechanistically distinct yet synergistic pathways. Initially, mechanical stimuli such as ultrasound, fluid flow, or physiological movement, induces pronounced crystal lattice distortion in piezoelectric nanomaterials, establishing intense internal electric fields via the mechanical polarization effect. Subsequently, these piezoelectric potentials markedly improve charge carrier separation kinetics, yielding an enhancement in quantum efficiency. Ultimately, the spatially separated electrons and holes drive concerted ROS generation through multiple routes: (i) superoxide radical (·O_2_^−^) formation via oxygen reduction, (ii) hydroxyl radical (·OH) production through water oxidation, and (iii) singlet oxygen (^1^O_2_) generation via energy transfer to molecularly engineered sonosensitizers. This multifaceted oxidative assault collectively creates a highly potent antimicrobial microenvironment capable of efficient pathogen eradication [[45], [46], [47], [48], [49]].

### 2.3 Piezoelectricity-driven antibacterial mechanism through physical disruption and multiple bioelectrical regulation

When subjected to mechanical stimuli, piezoelectric surfaces develop highly localized electric fields, which directly target bacterial cellular integrity and metabolic processes through several interconnected mechanisms [50,51]. The physical disruption effects in piezocatalytic antimicrobial systems operate through two primary mechanisms [52]. First, ultrasonic cavitation creates localized extreme conditions that mechanically compromise microbial structures, while resonant vibration induces μm-level oscillations at material-specific frequencies [53]. These combined physical actions produce three synergistic outcomes. First, mechanical shearing forces enable complete biofilm disintegration. Second, improved tissue permeability enhances the penetration of antimicrobial agents. Third, exceptional bacterial dispersion efficiency effectively prevents microbial recolonization. Together, these physical disruption mechanisms complement the electrochemical effects to achieve comprehensive antimicrobial efficacy. Moreover, the localized strong electric field generated on the surface of piezoelectric materials exerts potent antimicrobial effects through multiple bioelectrical regulation mechanisms. Primarily, the intense electric field directly disrupts bacterial membrane potential, causing transmembrane potential imbalance [14]. Subsequently, the electric field induces electroporation in lipid bilayers, compromising membrane structural integrity. At the molecular level, the field interferes with the activity of key enzymes in the electron transport chain (ETC), disrupting normal metabolic processes [54,55]. Ultimately, by inhibiting ATP synthase function, bacterial energy metabolism is severely impaired. This multilevel regulatory cascade, ranging from membrane potential interference to energy metabolism blockade, constitutes the unique antimicrobial mechanism of piezoelectric electric fields.

### 2.4 Intelligence multi-modal synergistic diagnosis and therapeutic action

Based on the aforementioned mechanisms, it can be concluded that the integration of the unique advantages of piezocatalytic antibacterial technology with intelligent design concepts positions it as a cutting-edge solution for addressing complex clinical challenges such as biofilm-related infections and deep-tissue infections. This technology achieves efficient sterilization through mechanochemical energy conversion, and its advancement toward intelligence is fundamentally centered on system designs that incorporate environmental sensing, adaptive regulation, and real-time feedback.

Firstly, its antibacterial activity is strictly dependent on the application of external mechanical stimuli, enabling precise spatiotemporal control characterized by “on-demand activation and immediate cessation”, thereby minimizing non-specific damage to healthy tissues. With the introduction of intelligent response modules, the system can autonomously adjust piezoelectric output based on biomarkers of the infected microenvironment (e.g., pH, specific enzymes, or metabolites) or real-time physiological signals, achieving dynamically adaptive and precise therapy [56,57]. In terms of applicability, this technology can be activated using high tissue-penetrating mechanical energy, such as ultrasound, or by directly coupling endogenous physiological mechanical energy like heartbeat and respiration. Through energy harvesting and management systems coupled with micro-sensor networks, the system can monitor the distribution of mechanical energy fields at the infection site in real time and optimize energy transfer efficiency, enabling highly targeted treatment of deep tissues [58,59].

From a mechanistic perspective, the technology does not rely on specific biochemical targets. By integrating artificial intelligence algorithms to optimize design parameters across multiple scales, including crystal phase engineering, defect modulation, and heterostructure construction, the system can autonomously adjust the intensity and mode of physicochemical synergistic effects, enabling personalized and programmable antibacterial strategies [60]. Notably, intelligent piezocatalytic antibacterial systems demonstrate self-learning and decision-making capabilities. By integrating biosensors and data processing units, the system can analyze changes in infection status in real time and dynamically adjust treatment protocols. Combined with wireless communication modules, remote monitoring and intervention of treatment processes can be achieved, offering a novel paradigm for clinical precision medicine. It is also important to highlight that next-generation systems are equipped with self-diagnostic and early warning functions. By monitoring trends in piezoelectric output signals, the system can detect early signs of biofilm formation or the development of drug resistance, enabling preventive interventions and truly achieving the goal of “integrated intelligent diagnosis and treatment”.

In summary, intelligent piezocatalytic antibacterial systems represent an interdisciplinary frontier that merges materials science, microelectronics, and artificial intelligence. By establishing a complete intelligent loop of “perception-decision-execution-feedback”, this technology opens new pathways for developing next-generation adaptive, programmable, and precise anti-infective diagnostic and therapeutic platforms, with the potential to fundamentally transform traditional treatment paradigms for infectious diseases precise diagnostic and therapeutic platforms for biofilm and deep-tissue infections [61,62].

## 3 Components and rational design of piezoelectric intelligent antibacterial systems

The design framework of piezoelectric intelligent anti-infection systems encompasses three core modules, forming a complete technological chain that spans from energy input and conversion amplification to functional output. This framework provides a clear logical pathway and design basis for the performance optimization and functional integration of the system.

Specifically, the antibacterial efficacy of piezoelectric catalytic systems represents a complex function governed by five interdependent factors that operate through synergistic interactions across multiple scales [50,52,60,62,63]. These factors include: (1) Intrinsic material properties, such as piezoelectric coefficients, phase structure, and band alignment, which determine the fundamental mechanoelectric conversion capability and catalytic potential and can be rationally designed using principles of coordination chemistry [64,65]; (2) Microstructural design strategies, including morphology control, defect engineering, and heterojunction construction, which directly regulate charge carrier dynamics, enhance the local electric field, and increase the density of active sites, with coordination chemistry providing controllable synthetic pathways at the molecular level [66,67]; (3) Mechanical energy input characteristics, such as stimulation type, intensity, and frequency, which must match the material's resonant behavior to maximize energy conversion [68]; (4) Factors within the infectious microenvironment, including pH, ionic strength, and oxygen concentration, which critically modulate reaction pathways and biological responses and can be leveraged to develop environmentally responsive piezoelectric materials [69,70]; (5) Integrated system design, which requires a multi-level strategy encompassing material optimization, external energy input tuning, environmental adaptation, and in-depth mechanistic studies [61].

Ultimately, improving the antibacterial activity of piezoelectric catalysis depends on the systematic integration of intelligent design throughout the entire system construction process. At the material level, combining machine learning and high-throughput computational methods enables the reverse design and performance prediction of piezoelectric materials. At the system level, integrating sensing, feedback, and control modules facilitates the construction of adaptive diagnostic and therapeutic platforms capable of real-time monitoring of infection status and dynamic adjustment of mechanical stimulation parameters, forming an integrated “sensing-decision-response” intelligent antibacterial system. Through such coordinated approaches, the antibacterial performance of piezoelectric catalytic materials can be significantly enhanced, thereby advancing their practical applications in biomedical and environmental remediation fields.

### 3.1 Selection of mechanical energy input

Given that the rational design of materials is both extensive and critical, we will discuss it in depth in a later section, and first introduce here the types and characteristics of input mechanical energy (Table 1). As the fundamental driving force of piezocatalysis, mechanical energy has traditionally been studied with a focus on single stimulus types (e.g., ultrasound). However, actively designing and regulating the type, intensity, and other characteristics of input mechanical energy can significantly optimize the performance of piezocatalytic materials.

**Table 1:** Piezoelectric effects and antibacterial mechanisms induced by different types of mechanical energy.

Mechanical Energy Type	Deformation Type	Electric Field Characteristics	Primary ROS	Antibacterial Efficiency	Typical Applications
Low-intensity ultrasound	Rapid periodic deformation	Strong local electric field	·OH, ·O_2_^−^, H_2_O_2_	High (minute-level sterilization)	Acute infections in deep tissues
Low-frequency vibration/weak mechanical forces (friction, pressure)	Periodic slight deformation	Mild electric field	Low-level ROS	Sustained inhibition (hour–day level)	Wearable antibacterial coatings
Water flow/wind energy	Local minor deformation	Low-potential electric field	Minimal ROS	Metabolic interference (long-term)	Flexible electronics, touch surfaces
Water flow/wind energy	Continuous irregular deformation	Sustained low-potential field	Trace ROS	Biofilm inhibition (continuous)	Water treatment, environmental purification
Biomechanical signals (muscle contraction, heartbeat, respiration)	Periodic micron-scale deformation	Endogenous physiological field	Controllable low-level ROS	Targeted, low side effects (long-term)	Implantable devices, in vivo therapy
Multi-mechanism synergy	Combined deformation	Synergistically enhanced field	Significantly elevated ROS	Anti-drug-resistant (synergistic enhancement)	Complex infections, multidrug resistance

In the presence of highly efficient piezocatalytic materials, even weak environmental stimuli (such as minimal mechanical energy) can activate the piezocatalytic effect, thereby avoiding tissue damage caused by high-energy external stimuli such as light, microwaves, or magnetism [71,72]. In fact, piezocatalytic antibacterial technology can utilize a wide variety of mechanical energy sources, ranging from high-intensity ultrasound to subtle biomechanical signals. These sources include externally applied mechanical forces (e.g., pressure, vibration, and friction), natural forces (such as water flow, wind energy, and biological motion), as well as environmental fluctuations (e.g., sound waves and temperature variations) [73,74]. Furthermore, artificial devices (e.g., piezoelectric energy harvesters) and microscopic-scale motions (e.g., molecular vibrations or nanoscale deformations) also serve as viable sources of mechanical energy [75,76].

Different types of mechanical energy induce distinct piezoelectric effects and antibacterial mechanisms. High-intensity ultrasound primarily induces rapid deformation of piezoelectric materials through high-frequency vibration, generating strong local electric fields that subsequently trigger the generation of large amounts of ROS, leading to efficient sterilization. In contrast, low-frequency vibration or weak mechanical forces (e.g., friction, pressure) cause periodic slight deformation of the material, producing relatively mild piezoelectric fields suitable for continuous, low-intensity antibacterial needs. Natural forces such as water flow or wind energy typically result in continuous, irregular mechanical stimulation, which induces sustained low-potential electric fields on the material surface, interfering with bacterial membrane potential and inhibiting bacterial growth. Biomechanical signals (e.g., muscle contraction, heartbeat, respiratory motion) can excite micro-scale deformation of implantable piezoelectric devices, generating endogenous electric fields compatible with the physiological environment, thereby achieving targeted antibacterial therapy with low side effects. Furthermore, the inherent energy conversion capability of piezoelectric materials allows for seamless integration with other therapeutic approaches to enhance antibacterial performance. This approach enables their adaptation to vastly different application scenarios, ranging from in vivo biomedicine to environmental remediation [77,78]. The core objective of this strategy is to achieve a precise match between the energy input and the target application.

From the perspective of energy-driven approaches and addressing antibacterial resistance challenges, in this section, we systematically review the energy input strategies of piezocatalytic systems. Currently, energy inputs can be primarily categorized into three major types: external mechanical energy, environmental kinetic energy, and endogenous physiological mechanical energy [[79], [80], [81]]. Each category demonstrates unique advantages in addressing specific bacterial resistance challenges. However, when these energy inputs are used together in a multimodal strategy, they exhibit complementary and sometimes synergistic effects. For example, combining ultrasound (high-frequency mechanical vibration) with light exposure (photoactivation) has been shown to significantly enhance ROS generation through cooperative mechanisms [82,83]. At the same time, depending on the parameters used, these energy inputs may also produce non-linear enhancement or, in some cases, mutual inhibition of antibacterial effects. More precise and adaptive energy integration may be the key to overcoming challenges such as biofilm formation, deep tissue penetration, and drug resistance [84,85].

#### 3.1.1 External mechanical energy stimulation

##### 3.1.1.1 Low-intensity ultrasound (LUS)

**Fig. 1:**
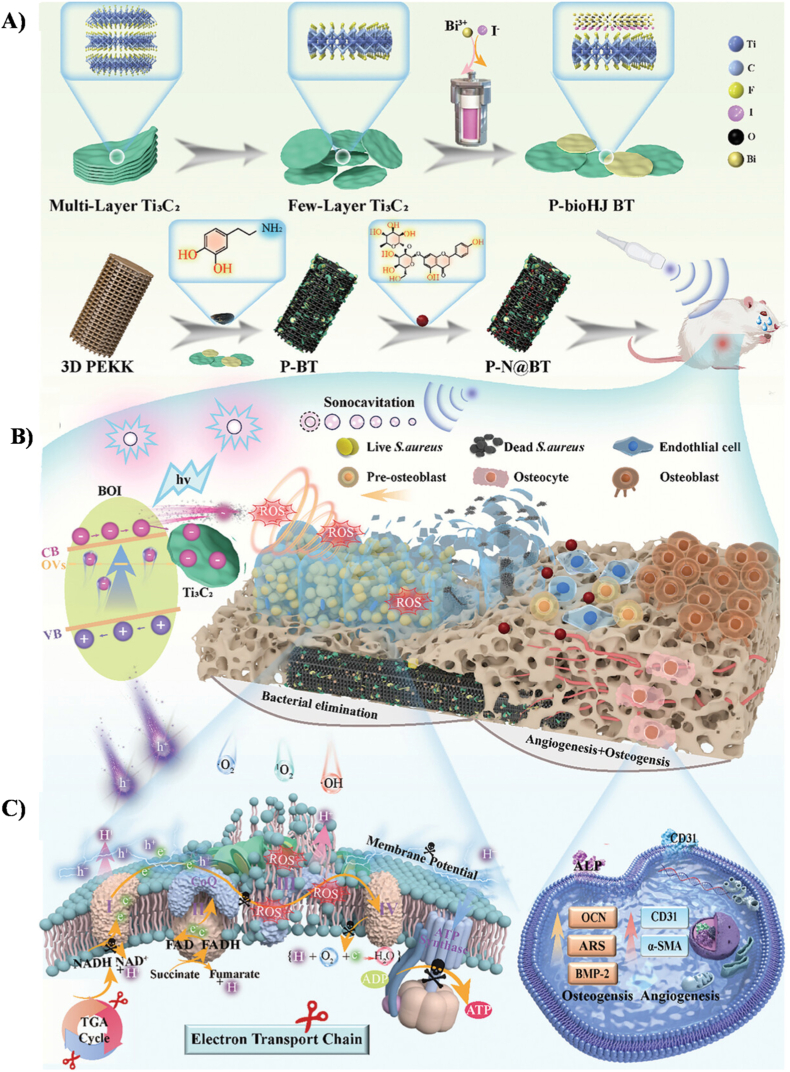
A) Preparation process of the P-bioHJ; B) LIU-activated piezocatalytic therapy; C) Proposed antibacterial and tissue repair mechanisms. Reproduced with permission [54]. Copyright 2025, Wiley-VCH GmbH.

As one of the most used mechanical energy sources, low-intensity ultrasound (LIU)-mediated piezoelectric catalytic therapy holds significant potential for remote treating deep intractable bacterial infections due to its non-invasive nature and ability to penetrate deep tissues, rather than being limited to the treatment of superficial infected wound. However, its effectiveness is often limited by the insufficient piezoelectric response of sensitizers under low-intensity ultrasound, leading to poor charge separation efficiency, reduced sterilization performance, and constrained catalytic activity. To address this challenge, Chen et al. developed a piezocatalytic bio-heterojunction (P-bioHJ) by integrating BiOI with a small amount of MXene to achieve rapid antibacterial effects (Fig. 1A). The engineered P-bioHJ features a narrow bandgap that responds to sonoluminescence generated by the acoustic cavitation effect, inducing interfacial polarization and oxygen vacancies. These characteristics enhance carrier separation and increase the yield of free radicals, leading to efficient sterilization. Transcriptomic analysis revealed that P-bioHJ exerts its antibacterial function by disrupting the bacterial electron transport chain and impairing metabolism and energy synthesis (Fig. 1B). In vitro experiments demonstrated excellent cytocompatibility of P-bioHJ, while in vivo studies confirmed its superior antibacterial performance in a low-intensity ultrasound (LIU)-mediated skin infection model. Furthermore, when combined with naringin, P-bioHJ promoted angiogenesis and osteogenesis in an infectious bone defect model (Fig. 1C). This study provides valuable insights into enhancing piezocatalytic therapy by leveraging the acoustic cavitation effect [86].

The aforementioned study represents a classic “material-centric” optimization approach. It focuses on improving the mechano-electro-chemical conversion efficiency at its source through band engineering and defect modulation, thereby providing a more effective tool for deep tissue anti-infection therapy. However, translating advanced materials into safe and reliable clinical applications requires further addressing challenges related to treatment precision, controllability, and biofunctionality. This necessity has driven research toward a higher level of “system integration and intelligent regulation”. In a subsequent work, Li et al. designed a metal-piezoelectric hetero-nanostructure on titanium (Ti) implants to achieve mechanical energy-driven antimicrobial properties for treating osteomyelitis [87]. Tschon et al. developed an intelligent injectable piezoelectric nanocomposite hydrogel system combined with low-intensity pulsed ultrasound therapy, and validated its efficacy in promoting cartilage regeneration in two preclinical models of osteoarthritis using rabbits and sheep. The research team innovatively established computational models based on the actual anatomical structures of the animals to simulate the propagation path and energy attenuation of ultrasound in tissues, thereby achieving precise control and optimization of the therapeutic dose. The piezoelectric hydrogel is loaded with autologous adipose-derived mesenchymal stem cells. Under ultrasound stimulation, it activates embedded barium titanate nanoparticles to generate endogenous bioelectrical signals, effectively promoting chondrogenic differentiation and suppressing joint inflammation. Furthermore, the study systematically evaluated for the first time the influence of sex on therapeutic response, revealing that female animals exhibited superior cartilage repair outcomes, providing important scientific evidence for individualized osteoarticular regenerative therapy. This system integrates smart materials, computational modeling, and physical stimulation, demonstrating promising potential for clinical translation [88].

Fungal infections, particularly those caused by pathogens such as Cryptococcus and Aspergillus, pose significant treatment challenges. Invasive candidiasis, for example, is difficult to manage due to its deep tissue penetration and tendency to form biofilms under the skin. To address these issues, researchers developed ZnO nanomaterials modified with carbonized wormwood (CMZ), which feature a narrow bandgap and responsiveness to yellow light (YL) and ultrasound (US). Under combined YL and US irradiation, CMZ generates abundant ROS through piezoelectric-photocatalytic action. These ROS disrupt genes associated with fungal virulence, metabolic activity, hyphal growth, and biofilm formation, effectively eradicating both planktonic *Candida albicans* and mature biofilms. Remarkably, CMZ demonstrated the ability to inactivate 99.9% of *C. albicans* even through a 1 cm tissue barrier, showcasing its potential for deep antifungal therapy in *vitro* [89].

##### 3.1.1.2 Environmental kinetic energy (EKE)

As a widely distributed and sustainable form of green energy, environmental kinetic energy demonstrates significant potential in constructing intelligent clean energy systems, capable of efficiently converting the abundant mechanical energy in the environment into electrical energy. Such systems not only exhibit excellent output performance and lightweight structures but also show potential for integration with intelligent sensing and feedback systems, providing a foundation for self-powered and self-regulating intelligent environmental governance systems. In recent years, smart or intelligent antibacterial systems based on piezoelectric materials have emerged as a research hotspot, as they can autonomously respond to external mechanical stimuli to achieve efficient and controllable microbial inactivation. Roy et al. developed an intelligent piezoelectric polymer film enhanced with barium titanate nanorods. This material can generate a piezoelectric response under various mechanical stimuli (such as ultrasound, water flow, and stirring) and further produce highly ROS in situ through a piezocatalytic mechanism, enabling intelligent and responsive inactivation of various bacteria, including *E. coli* and *S*. *aureus*. The study further constructed a continuous-flow reaction system driven by fluid kinetic energy, which can autonomously sense fluid dynamics and continuously convert environmental kinetic energy into antibacterial activity. This system achieves chemical-free, self-regulating, and intelligent water disinfection, providing a new approach for developing environmentally responsive water treatment technologies [90]. Poudel et al. designed an intelligent wind energy harvesting-driven disinfection system based on the galloping effect. This system can adaptively adjust its output characteristics according to changes in wind speed and utilizes the electroporation principle to achieve precise and efficient bacterial inactivation. Operating at a driving voltage of approximately 0.1 V for 25 min, the system achieved an efficient inactivation of *Escherichia coli* with a reduction of approximately 2.33 log, demonstrating its potential for constructing self-powered, intelligent, and distributed water disinfection systems in remote or power-scarce areas [91]. These studies collectively demonstrate the feasibility of piezoelectric materials in achieving intelligent antibacterial responses driven by environmental kinetic energy. They not only realize energy self-sufficiency and process autonomy but also highlight the system's adaptive capability to external conditions.

#### 3.1.2 Internally generated physiological mechanical energy stimulation (PMES)

The management of deep tissue infections represents a significant clinical challenge due to limited penetration of conventional therapies and the persistent risk of systemic dissemination [92,93]. The core of addressing this challenge lies in the development of intelligent systems capable of efficiently harvesting and converting the body's endogenous micro-energy. Leveraging inherent physiological mechanical energy, such as cardiac pulsation, respiratory movements, muscle contractions, and gastrointestinal peristalsis, enables the creation of self-powered, long-term, and sustained antibacterial platforms [94,95]. These intrinsic stimuli are characterized by ultra-low frequency (<10 Hz), low amplitude, and non-periodicity, imposing stringent requirements on the low-frequency response and energy harvesting efficiency of piezoelectric materials. To overcome these hurdles, research strategies have converged on several fronts: advancing flexible piezoelectric polymers (e.g., PVDF and its copolymers) that, with their low Young's modulus, conform effectively to soft tissue micro-deformations; designing hierarchical or porous architectures to amplify strain transduction and energy conversion; and integrating power management circuitry to rectify, store, and controllably release the generated electrical energy [96,97].

**Fig. 2:**
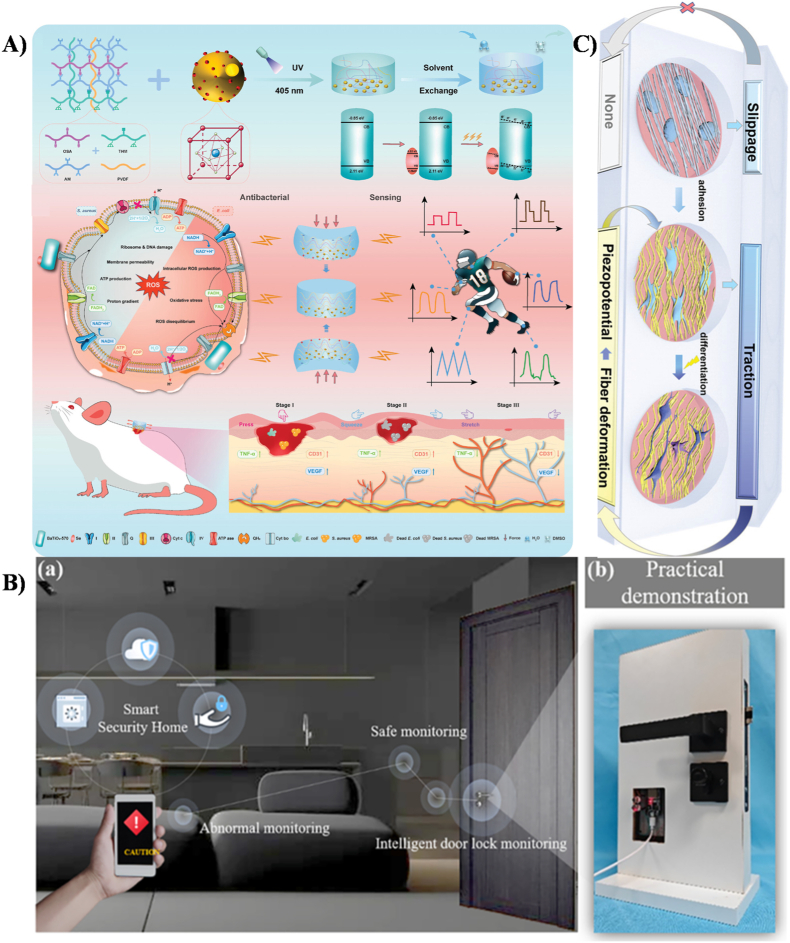
Schematic illustration of integrated smart electroactive systems for bio-sensing, therapy, and monitoring.(A) Dynamic wound monitoring and antibacterial piezoelectric hydrogel dressing: An OAPS hydrogel enables real-time motion sensing and electron-transfer-based antibacterial therapy under mechanical deformation, facilitated by UV crosslinking and solvent exchange. (B) Cell-traction-mediated on-demand piezoelectric stimulation for stem cell differentiation: After mature FAs form, cell-generated traction deforms fibers, generating piezopotential to stimulate stem cell differentiation. This provides in situ, wireless, and on-demand electrical stimulation without external triggers. (C) Self-powered security monitoring with a flexible piezoelectric nanogenerator (PENG): A NN/P(VDF-TrFE)-based PENG sensor is applied for smart home safety, enabling real-time monitoring of door lock status and anomaly detection [67,98,99]. Copyright 2025, Wiley-VCH GmbH; Copyright 2021, Wiley-VCH GmbH; Copyright 2024, Elsevier Ltd.

For instance, Liu et al. developed an intelligent heterogeneous piezoelectric antibacterial asymmetric hydrogel (OAPS) specifically designed for dynamic management of deep wounds and associated infections (Fig. 2A). Fabricated via photochemical cross-linking with Se-doped KH570-modified BaTiO_3_/Se heterojunction nanoparticles as the functional core, this hydrogel intelligently harvests and converts micro-mechanical energy from high-frequency body movements. Without external power, it utilizes the piezoelectric effect to drive charge transfer, achieving a highly efficient electron-transfer-mediated antibacterial action (>99.6% efficacy against *S. aureus*, *E. coli*, and MRSA). Simultaneously, it transduces mechanical motion into electrical signals for real-time, intelligent monitoring of wound status. In vivo experiments demonstrated its remarkable ability to promote the healing of infected deep wounds, achieving a healing rate of 99.75% within 14 days. This work establishes a novel strategy for intelligent, theranostic wound management in deep tissue by harnessing the body's own energy [67].

Deng et al. engineered a self-powered, flexible piezoelectric nanogenerator (PENG) sensor based on a NaNbO_3_/P(VDF-TrFE) composite film, highlighting its potential for integrated monitoring in contexts where deep infection risks exist, such as post-surgical or implant sites (Fig. 2B). Fabricated via electrospinning, the highly aligned NaNbO_3_ particles significantly promoted the β-phase formation in PVDF-TrFE (reaching 91%), markedly enhancing the piezoelectric response. Under mechanical stimulation at approximately 3 Hz, the PENG generated output voltages of 17.3 V and 12 V in transverse and longitudinal modes, respectively, with a maximum power density of 0.67 μW/cm^2^ under a 100 MΩ load. This sensor intelligently harvests weak mechanical energy from bodily movements, demonstrating high sensitivity and rapid response in activity monitoring. Its capability for real-time status sensing underscores its potential as a component in intelligent, self-powered systems for early warning and monitoring in scenarios prone to deep-seated infections [98]^.^

Liu et al. developed an intelligent piezoelectric scaffold based on poly(vinylidene fluoride) (PVDF) nanofibers, which innovatively utilizes the cell's own traction force as the driving power source to achieve on-demand, self-feedback electrical stimulation (Fig. 2C). The scaffold possesses mechanical stiffness similar to collagen and can deform in response to cellular traction forces after cell adhesion and mature focal adhesion formation, thereby intelligently converting mechanical energy into electrical signals (piezopotential) to specifically promote stem cell differentiation toward a neuronal lineage. This endogenous, cell-driven stimulation approach avoids interference with cell spreading and early adhesion. For the first time, a constant material system achieves adaptive regulation of the dynamic cellular microenvironment, providing a new paradigm for designing next-generation intelligent tissue engineering scaffolds [99].

In summary, current research consistently demonstrates that piezoelectric material-based intelligent diagnosis and treatment systems exhibit significant feasibility and superior intelligent capabilities for the prevention and treatment of deep tissue infections. Depending on the specific clinical application scenarios, corresponding mechanical energy sources can be selectively utilized. These systems not only achieve self-powered energy supply and adaptive regulation of the treatment process but also dynamically optimize response strategies based on the real-time physiological microenvironment, thereby laying a solid foundation for the development of next-generation intelligent diagnosis, treatment, and monitoring platforms tailored to diverse clinical needs.

### 3.2 Rational design of piezoelectric material for antibacterial applications

Piezoelectric materials demonstrate distinctive advantages in catalysis and antibacterial applications owing to their unique mechano-electrical conversion mechanism. Their catalytic activity originates from the charge separation induced by mechanical stress: the relative displacement of positive and negative charge centers within the crystal generates a polarized electric field, which drives the creation and migration of surface electron-hole pairs, thereby catalyzing the production of ROS and other free radicals [100]. This process relies not only on the non-centrosymmetric crystal structure of the material but can also be systematically regulated through precise design of the microstructure.

**Fig. 3:**
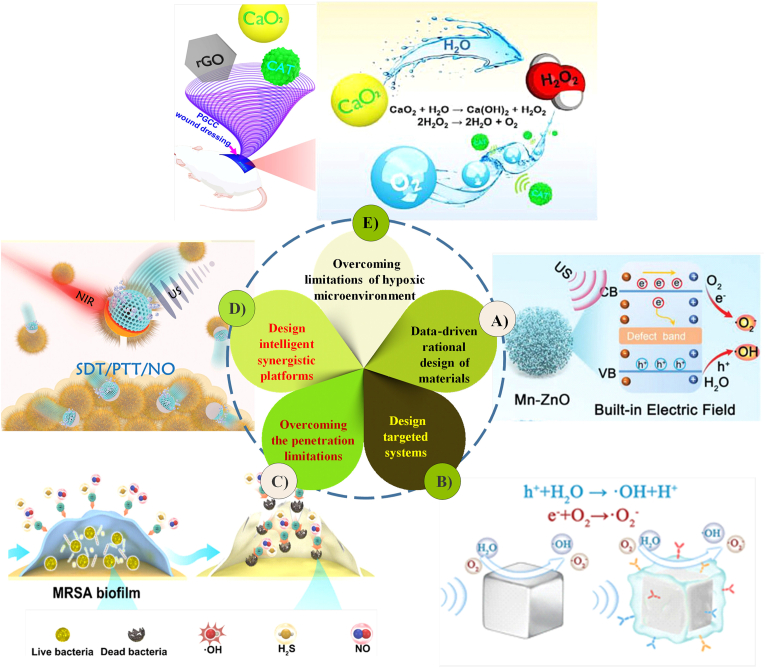
Typical multi-level synergistic modulation strategies to improve the antibacterial efficiency of piezoelectric semiconductors for various applications [[101], [102], [103], [104], [105]]. Copyright 2023, American Chemical Society; Copyright 2024, American Chemical Society; Copyright 2024, Wiley-CH; Copyright 2023, Elsevier; Copyright 2024, Wiley-CH.

To enhance the catalytic and antibacterial performance of such piezoelectric materials, as shown in Fig. 3, current research focuses on multi-level synergistic modulation strategies with the core logic of addressing key bottlenecks in ROS therapy, such as low efficiency, poor targeting, and limitations imposed by the lesion microenvironment, through material design and functional integration [[101], [102], [103], [104], [105]]. At the material design level, systematic optimization of the piezoelectric coefficient and charge separation efficiency can be achieved through rational selection of material systems, implementation of crystal phase engineering, precise defect modulation, and construction of heterostructures. Furthermore, rational design of the coordination environment between central metals and ligands based on coordination chemistry principles enables fine-tuning of the material's band structure and surface catalytic activity [20,106,107]. To overcome the barrier of biofilms, the system employs a penetrable nanoplatform to penetrate deep into the biofilm, where ROS, together with signaling molecules such as H_2_S and NO, eliminate viable bacteria and disrupt the biofilm structure. In terms of synergistic therapy, a near-infrared light-excited sonodynamic/photothermal/NO combined platform can simultaneously achieve thermal killing and oxidative killing, while activating the immune response. To address the issue of hypoxia at the lesion site, calcium peroxide (CaO_2_) can continuously generate oxygen and hydrogen peroxide through its reaction with water, which not only alleviates hypoxia but also provides sufficient substrates for ROS production. Furthermore, a multifunctional wound dressing loaded with CO, CaO_2_, and catalase has been shown to synergistically promote wound healing in a living mouse model through anti-inflammatory effects, sustained oxygen supply, and enzymatic regulation. Overall, this figure comprehensively demonstrates the full-chain technical pathway from laboratory mechanism verification to in vivo application, covering structural optimization, targeted design, penetration breakthrough, synergistic construction, and hypoxia improvement, thus achieving an upgrade from single ROS killing to multimodal precise synergistic therapy.

#### 3.2.1 Data-driven rational selection and design of piezoelectric materials

Material design, as a core driving force in modern technological advancement, has long been constrained by a traditional research paradigm reliant on extensive repetitive experimentation, leading to prolonged development cycles and low efficiency [108,109]. In recent years, as shown in Table 2, data-driven approaches integrating first-principles calculations and artificial intelligence have been systematically transforming the paradigm of new material design, particularly in the field of high-performance piezoelectric materials [124]. First-principles calculation methods represented by Density Functional Theory (DFT) enable precise prediction of key physicochemical properties, such as electronic structure, bandgap characteristics, interfacial behavior, and piezoelectric coefficients, from the atomic scale, providing a theoretical foundation for understanding the piezoelectric effect and its applications in catalysis and photocatalysis. However, given the vast space of candidate materials, relying solely on theoretical computations remains insufficient for efficiently screening materials that combine excellent performance with synthetic feasibility [61,125].

**Table 2:** Core roles and representative applications of DFT calculations and machine leaning in the rational design of antibacterial piezoelectric nanomaterials.

Optimization Dimension & Objective	Key DFT Calculation Descriptors/Methods	Physicochemical Significance	Guidance and Prediction for Material Performance	Typical Application Examples	Methods	Ref.
**1.** Electronic structure modulation (Optimizing charge separation & transfer)	• Band structure calculations • Density of states analysis • Work function and band edge positions	Reveals intrinsic electronic properties, carrier types, excitation thresholds, and the direction and quantity of interfacial charge transfer.	Guides bandgap engineering to screen optimal doping elements or heterojunction pairs, enabling efficient carrier generation, separation, and migration, thereby enhancing ROS generation capacity.	Bi_4_O_5_Br_2_@Ru	DFT	[110]
				TiO_2_/Bi_2_WO_6_	DFT	[111]
				P-bioHJ	DFT	[54]
				BiFeO_3_/ZnIn_2_S_4_/Ag	DFT	[112]
				Bi_4_O_5_Br_2_@Ru	DFT	[113]
				UHf NPs	DFT + VASP	[114]
**2.** Surface adsorption & activation (Enhancing substrate capture & conversion	• Piezoelectric coefficient calculations • Strain-band structure relationships • Excited-state simulations	Quantitatively evaluates the material's surface affinity for key reactants, their activation degree, and the electronic interaction mechanism.	Rationally designs materials with high-adsorption-energy surfaces to preferentially capture and activate substrate molecules, overcoming environmental limitations such as hypoxia or high GSH levels at infection sites.	(BiFe)_0.9_(BaTi)_0.1_O_3-x_	DFT	[115]
				CFO@MoS_2_	DFT	[116]
				PLLA-ZnO/RPLLA	DFT	[117]
				1T/2H MoS_2_-x	DFT	[118]
				UHf NPs	DFT + VASP	[114]
**3.** Catalytic reaction pathway analysis (Reducing reaction energy barriers, improving efficiency)	• First-principles molecular dynamics • Free energy calculations	Depicts the full catalytic reaction pathway at the atomic scale, identifies the rate-limiting step, and quantifies the contribution of modification strategies to lowering reaction barriers.	Clarifies key bottlenecks in performance optimization and guides the direct modification of the RDS energy barrier through active site engineering (e.g., single-atom doping) to achieve multiplicative gains in catalytic efficiency.	(BiFe)_0.9_ (BaTi)_0.1_O_3-x_	DFT	[115]
				Bi_4_O_5_Br_2_@Ru	DFT	[110]
				ReS_2_@C-40	DFT	[119]
				MoSe_2_	DFT	[101]
				PtRu/C_3_N_4_	DFT	[120]
**4.** High throughput screening platform (Screening suitable materials rapidly)	• Automated workflows integrating DFT and ML • Active learning-guided computations • Intelligent mining of material databases	• Rapid screening of thousands of candidate materials. • Active learning guides focused computational regions. • Inverse design of materials meeting multiple objectives.	This intelligent computational framework achieves precise prediction and efficient design from performance targets to material structures through the triple synergy of high-throughput screening, active learning, and inverse design.	BaTiO_3_	ML	[121]
				MXene	ML	[122]
				H-TPMS	ML	[123]

The introduction of machine learning (ML) has significantly enhanced the efficiency and precision of material discovery [126,127]. By integrating high-throughput computing with machine learning models, it is possible to establish a relationship map between material structure and piezoelectric properties, thereby enabling rapid prediction of piezoelectric response and stability in new systems [128]. Feature engineering plays a crucial role in the model construction process. In this process, feature engineering plays a critical role in model construction. To address the challenge of discovering high-performance piezoelectric materials, Hu et al. conducted a systematic study aimed at designing and evaluating advanced machine learning models for predicting piezoelectric moduli from material compositions and structures. The authors trained multiple prediction models by combining extensive feature engineering with various machine learning models, and by introducing automated feature learning methods based on deep graph neural networks. Using these models, they predicted the piezoelectric coefficients of 12,680 materials and identified the top 20 potential high-performance piezoelectric materials [126,127].

In terms of feature construction and selection, as exemplified in another work by Li et al., intrinsic atomic properties such as atomic radius, electronegativity, and ionization energy were selected as initial descriptors, and the Lasso algorithm was employed for feature selection to enhance model generalizability and interpretability. Furthermore, by incorporating active learning strategies with optimization methods such as Expected Improvement, the system can intelligently screen the most promising candidate materials for subsequent computational validation. In their study, machine learning models successfully identified over 140 MXene materials with high piezoelectric stress coefficients (e_31_ > 3 × 10^−10^ C/m), substantially improving the efficiency of material screening [122].

Regarding model performance, various models, including random forest regression, gradient boosting regression, support vector regression, and multilayer perceptron regression, demonstrated strong predictive capabilities for MXene systems. Among these, support vector regression achieved a coefficient of determination (R^2^) of 0.89 on the single-atom MXene test set, confirming its effectiveness in screening materials with high piezoelectric coefficients. Additionally, by combining natural language processing with historical experimental data, artificial intelligence can recommend synthetic pathways and enable automated material preparation and characterization through autonomous experimental platforms.

Beyond predictive functionality, machine learning can also extract key physical descriptors influencing piezoelectric performance from high-dimensional feature spaces through methods such as symbolic regression and multi-task learning, thereby establishing interpretable structure-property relationship models [129,130]. Combined with first-principles calculations, these approaches can further elucidate the modulation mechanisms of external stress on material electronic structures and clarify the microscopic mechanisms of “mechanical-electrical-chemical” coupling processes, providing deeper theoretical guidance for rational material design [61,127].

In summary, by integrating artificial intelligence with computational materials science, researchers can not only efficiently screen candidate materials with suitable bandgaps, high piezoelectric coefficients, and good stability but also significantly shorten the cycle from theoretical prediction to experimental validation through digital twins, cloud computing, and other means. Looking ahead, with further advancements in autonomous laboratories and general artificial intelligence models, the design and development of piezoelectric antibacterial nanomaterials are expected to achieve higher throughput, stronger interpretability, and broader applicability.

#### 3.2.2 Selecting appropriate piezoelectric materials based on application scenarios

Based on their chemical composition, piezoelectric nanomaterials can be primarily classified into three categories: inorganic piezoelectric nanomaterials, organic piezoelectric nanomaterials, and organic-inorganic hybrid piezoelectric nanomaterials. The intrinsic properties of these materials directly determine their core application domains.

Inorganic piezoelectric materials (e.g., BaTiO_3_, ZnO) form the core of traditional systems and typically exhibit high piezoelectric coefficients and excellent chemical stability. Their piezoelectric performance fundamentally depends on symmetry breaking in the crystal structure and distortion of cation coordination polyhedra (e.g., TiO_6_ octahedra). Through doping, defect engineering, or constructing nano-heterostructures (e.g., core-shell, heterojunctions), their coordination environment and local polarization can be regulated at the atomic/nanoscale. For example, introducing metal ions of different valence states or modulating oxygen vacancy concentrations can effectively tune the band structure and carrier density of the material, thereby optimizing its mechanical-electrical-chemical energy conversion efficiency. It is noteworthy that Yuan et al. proposed a framework combining machine learning and feature engineering for efficiently screening barium titanate-based ceramic materials with high piezoelectric constants. Their study compared multiple feature selection strategies and found that models based on domain knowledge and gradient boosting trees were most instructive. Within just two iterative cycles, an optimized material with a piezoelectric constant of approximately 430 pC/N was experimentally synthesized from nearly nine million candidate compositions, fully demonstrating the effectiveness of data-driven methods in accelerating the development of high-performance piezoelectric materials [121].

Because of their high piezoelectric coefficients, high stiffness, and excellent thermal stability, inorganic piezoelectric materials are primarily employed in applications demanding high output performance and reliability, such as high-precision sensors, high-power energy harvesters, and micro-actuators, where they serve as essential functional components. However, most inorganic materials are relatively brittle and face challenges in mechanical compatibility and long-term biocompatibility with biological tissues [[131], [132], [133], [134]].

In contrast, organic piezoelectric nanomaterials (exemplified by PVDF and its copolymer nanofibers) dominate applications that require conformal integration with the human body or complex curved surfaces, owing to their outstanding flexibility, low density, excellent biocompatibility, and ease of processing. Typical applications include wearable health monitoring devices, flexible self-powered sensors, and biomedical scaffolds or tissue engineering constructs.

To overcome the limitations of single-component systems in integrating performance and functionality, organic-inorganic composite piezoelectric materials have emerged as a significant frontier in current research. These materials aim to synergistically combine the high piezoelectric responsiveness of inorganic components with the flexibility, processability, and favorable biocompatibility of organic components such as polyvinylidene fluoride (PVDF) and its copolymers, thereby achieving enhanced overall performance [97,135]. The core design principle hinges on interface engineering driven by coordination chemistry, which entails constructing directional intermolecular interactions, such as coordination bonds, hydrogen bonds, or π–π stacking, at the organic-inorganic heterointerface. This approach enhances mechanical coupling, improves stress transfer, and promotes interfacial charge separation. This not only enhances the mechanical integrity and piezoelectric output of the material but also optimizes the dynamics of photo- or piezo-induced charge carriers through band structure regulation, thereby significantly boosting its catalytic efficiency for generating ROS.

With the revolutionary development of the Internet of Things and smart wearable industries, piezoelectric materials have garnered increasing attention. To address the limitations of piezoelectric materials in wearable applications, scientists have combined highly piezoelectric inorganic oxides with flexible organic polymer matrices to develop ceramic-polymer nanocomposites, significantly enhancing the output performance of flexible piezoelectric devices. During energy conversion processes, the interfacial coupling between oxide fillers and the polymer matrix is a key determinant of the electromechanical coupling efficiency in nanocomposites. However, the effects of the geometric morphology, spatial orientation, and material constants of oxide fillers on the effective performance of nanocomposites have not yet been systematically studied, posing significant challenges to the design and fabrication of high-performance piezoelectric nanocomposites. To address these challenges, Li et al., investigated the stress transfer efficiency, effective dielectric constant, and piezoelectric coefficient of polymer/ceramic nanocomposites using a method combining high-throughput phase-field simulation and machine learning [136]. Through high-throughput phase-field simulations, the effects of the morphology and spatial orientation of oxide fillers on the piezoelectric, mechanical, and dielectric properties of piezoelectric nanocomposites were systematically revealed.

It should be pointed out that metal-organic frameworks (MOFs), characterized by their porous coordination structures, hybrid polar nature, and tunable chemical functionalities, have demonstrated considerable potential as high-performance triboelectric/piezoelectric functional units. Their large specific surface area, adjustable porosity, and flexible topology also provide an ideal platform for energy harvesting and conversion. However, the efficient integration of multifunctional MOFs with triboelectric/piezoelectric nanogenerators remains challenging, particularly in terms of structural compatibility, interfacial synergy, and device stability, which require further in-depth investigation [137,138]. Although there is currently no literature on using machine learning to enhance the piezoelectric performance of MOFs, a substantial body of research demonstrates that machine learning has been widely applied in the structural design, optimization, and various applications of MOF materials. Therefore, in future research, applying machine learning to the screening and design of MOF materials with piezoelectric properties represents a highly promising direction. Typically, further applying the principles of coordination chemistry to develop novel bio-based metal-organic frameworks with precisely tailored interfaces, biodegradability, and environmental friendliness, and advancing their integration with highly sensitive, broadband energy harvesting devices, holds significant scientific and application value. On this basis, integrating machine learning and artificial intelligence technologies can enable the high-throughput screening of novel bio-based metal-organic framework materials with excellent piezoelectric response and biocompatibility, while autonomous experimental platforms can facilitate their controlled synthesis and performance optimization. By advancing the integration of such materials with high-sensitivity, broadband energy harvesting devices, it is anticipated that a new generation of intelligent piezoelectric systems, combining high performance, stability, and favorable biocompatibility, can be developed, offering innovative solutions for fields such as wearable medical devices and smart antibacterial materials.

The classification of piezoelectric materials have been addressed in earlier reviews and will not be examined in detail here [23,24,28]. Instead, in Table 3, the fundamental characteristics, advantages and disadvantages, mechanisms of action, antibacterial performance, and applicable fields of various piezoelectric materials are systematically summarized, aiming to provide readers with clear guidance for selecting suitable material types for specific applications.

**Table 3:** Comparison of the performance of different types of piezoelectric materials for antibacterial therapy.

Types	Advantage and disadvantage	Materials	Optimization method (Coordination/Defect)	Key performance metric (Piezoelectric)	Instrument	Antibacterial Condition	Antibacterial Mechanism	Antibacterial Efficacy	Application	Ref.
Inorganic piezoelectric materials	Advantage: high piezoelectric performance; excellent thermal stability Disadvantage: high brittleness and low fracture toughness	TiO_2_/BTO	Ba^2+^ doping (TiO_2_:Ba)	Lattice distortion & polarization enhancement	Xenon Lamp Ultrasonic Device	N/A	ROS (•O_2_^−^, •OH) under US + Light	N/A	Degrade organic pollutants	[139]
N/A N/A		ZnO NWs@TiO_2_-xNy	N-doping (substitutional & interstitial)	N/A	Xenon Lamp Ultrasonic cleaner	300 W Visible light 200 W US	ROS (•O_2_^−^, •OH) under US + Light	100% (*E. coli*, 40 min) 95% (*S. aureus*, 60 min)	N/A	[140]
		BTO	N/A	N/A	N/A	100 μg/ml BTO	N/A	Inhibition zone: 16–18 mm (*P. aeruginosa*), 16–18 mm (*S. aureus*)	N/A	[141]
		Vo-BTO	Oxygen vacancy defects (via NaBH_4_ thermal reduction, optimal at 400 °C)	N/A	Ultrasonic cleaner	1.5 W/cm^2^, 1 MHz US 800 μg/ml BTO-400	ROS (•OH, ^1^O_2_) under US	100% (*E. coli*, 4 min) 99% (*S. aureus*, 4 min)	Promote wound healing	[142]
		ZnO	N/A	N/A	Ultrasonic cleaner	90 W US 0.1 g/mL ZnO	Physical disruption + ROS	*S. aureus*	Infection-preventing biomedical implants	[143]
		ZnO@Bdell0	Surface functionalization modification (HS-PEG2000-NHS links ZnO with Bdellovibrio bacteria)	N/A	N/A	N/A	ROS (•OH, •O_2_^−^)	*F. nucleatum, E. coli, P. gingivalis, T. forsythia*	Treat periodontitis	[144]
		ZnO@TiO_2-x_	Oxygen vacancies in the TiO_2_ shell (annealed in Ar)	Piezoelectric coeff, d33 = 25 p.m./V	Chattanooga 2776	2.5 W/cm^2^, 1 MHz US	ROS (•OH, •O_2_^−^, ^1^O_2_) under US	98.53% (*S. aureus*, 30 min) 98.05% (MRSA, 30 min)	Treatment of osteomyelitis	[145]
		PDMS/WS_2_NFs	Native vacancies (sulfur vacancies Vs^−^ and tungsten vacancies V_W‴)	PFM piezoelectric response signal of 24 mV; TUNA piezoelectric current of 5 nA	Ultrasonic vibrator	300 W, 40 kHz US	ROS (•OH, •O_2_^−^) under US	99.99% (*E. coli*, 60 min)	Degrade organic pollutants	[146]
		WS_2_	N/A	Piezoelectric coeff, d33 = 2.12 p.m./V	Ultrasonicator	150 W, 40 kHz US	ROS (•O_2_^−^, •OH) under US	99% (*E. coli*,120 min)	Degrade organic pollutants	[133]
		Vs-B/MoS_2_	Dual-defect engineering: sulfur vacancies; boron doping	Piezoelectric coeff, d33 = 35.1 p.m./V; PFM amplitude = 403.9 p.m.; PFM potential = 72.2 mV	Ultrasonic cleaner	N/A	ROS (•O_2_^−^, •OH) under US	100% (*E. coli*,12 h)	Antibiotic degradation	[147]
		HNTM-MoS_2_	Heterointerface engineering	Piezoelectric response (butterfly loop + 180° phase switching)	N/A	1.5 W/cm^2^, 1 MHz US 500 μg/ml HNTM-MoS_2_	ROS (^1^O_2_, •O_2_^−^) under US	98.5% (MRSA, 15 min)	Bone infection therapy	[132]
Inorganic piezoelectric materials	Advantage: high piezoelectric performance; excellent thermal stability Disadvantage: high brittleness and low fracture toughness	g-ZnN_4_-MoS_2_	N/A	Piezoelectric coeff, d33 = 20.4 p.m./V	Intelect mobile ultrasound(Chattanooga 2776)	1.5 W/cm^2^, 1 MHz US 400 μg/ml g-ZnN_4_-MoS_2_	ROS (^1^O_2_) under US	99.58% (MRSA, 20 min)	Bone infection therapy	[148]
		MoS_2_/Fe_S_	N/A	Bandgap narrowing (ΔEg = −0.25 eV)	Microwave	0.1 W/cm^2^, 2.45 GHz US 1 mg/ml MoS_2_/Fe_S_	ROS (^1^O_2_, •O_2_^−^) under Microwave	100% (*S. aureus*,20 min) 100% (*E. coli*, 20 min)	Treatment of osteomyelitis	[149]
		MoS_2_/Fe_3_O_4_	Heterointerface engineering	N/A	Microwave	0.1 W/cm^2^, 2.45 GHz US 1 mg/ml MoS_2_/Fe_3_O_4_	ROS (^1^O_2_, •O_2_^−^) under Microwave	100% (*S. aureus*,20 min) 100% (*E. coli*, 20 min)	Treatment of osteomyelitis	[150]
		MoSe_2_	N/A	Piezoelectric coeff, d33 = 18.5 p.m./V	N/A	0.3 W/cm^2^, 1 MHz US 100 μg/ml MoSe_2_	ROS (•O_2_^−^, •OH) under US	99.93% (*E. coli*) 99.99% (MRSA)	Deep-seated biofilm infection	[101]
		ReS_2_	N/A	Piezoelectric coeff, d33 = 22.1 p.m./V	N/A	100 mW/cm^2^ Visible light 100 W, 40 kHz US 1 mg/ml ReS_2_	ROS (•O_2_^−^, •OH) under US	99.99% *(E. coli*, 30 min) 96.67% (*S. aureus*, 30 min)	Water sterilization	[119]
		FeWO_4_	N/A	Piezoelectric response (butterfly loop + 180° phase switching)	N/A	1 W/cm^2^, 1 MHz US 0.1% (w/v) FeWO_4_	ROS •O_2_^−^, •OH) under US	81% (*S. aureus*, 4h)	Promote wound healing	[151]
		P-bioHJ	Heterointerface engineering; Surface oxygen vacancy defects	Piezoelectric coeff, d33 = 129.22 p.m./V	N/A	0.01-3 W/cm^2^ US	ROS (•O_2_^−^, •OH, ^1^O_2_) under US	99.85% (*S. aureus*, 10 min) 99.3% (*E. coli*, 10 min)	Infected bone defect repair	[86]
		Bi_4_O_5_Br_2_@Ru	Ru-doping	O_2_ adsorption energy (ΔEads) from −3.188 to −3.506 eV	N/A	1 W/cm^2^, 1 MHz US 200 μg/ml Bi_4_O_5_Br_2_@Ru	ROS (^1^O_2_, •O_2_^−^) under US + Light	99% (*E. coli*, 10 min) 99% (MRSA, 10 min)	Deep bacterial abscess	[110]
		TiO_2_/Bi_2_WO_6_	Heterointerface engineering	N/A	NIR laser	1 W/cm^2^ light	ROS (•O_2_^−^, •OH) under Light	99.03% (*E. coli*,10 min) 99.11% (MRSA, 10 min) 98.31% (*P. gingivalis*, 10 min)	Osseointegration	[111]
Organic piezoelectric materials	Advantage: flexibility and mechanical adaptability; biocompatibility and low toxicity Disadvantage: high fabrication cost; poor biocompatibility	PVDF-TrFE	Ionic liquid doping	Piezoelectric coeff, d33 = 34 p.m./V	N/A	10/20 wt%, IL PVDF-TrFE	N/A	N/A	Orthopedic implant coating	[152]
ezoelectric organic-inorganic composites	Advantage: synergistic enhancement effect; optimized mechanical properties; Disadvantage: interfacial challenges, processing limitations	PVDF/4Ag-pBT	Surface modification with Ag Nps	Piezoelectric coeff, d33 = 8.2 p.m./V	N/A	N/A	ROS (•O_2_^−^) under US	81% (*E. coli*, 24 h)	Bone scaffold	[153]
		ZnO@GDY	Coordination with graphene derivative	Piezoelectric coeff, d33 = 14.4 p.m./V	DM-200F	1 W/cm^2^, 1 MHz US 200 μg/ml ZnO@GDY	ROS (•OH, •O_2_^−^, ^1^O_2_) under US	99.99% (MRSA, 30min) 99.99% (*P. aeruginosa*, 30 min)	Promote wound healing	[154]
		ZnO@HTCS	Schottky heterojunction interface; Surface oxygen vacancy defects	Piezoelectric field: ZnO@HTCS 2.43 × 10^6^ V/m; Bandgap: 1.37 eV	Intellect mobile ultrasound (Chattanooga 2776)	1.5 W/cm^2^, 1 MHz US 50 μg/ml ZnO@HTCS	ROS (•OH, ^1^O_2_) under US	99.78% (MRSA, 15 miin)	Treatment of osteomyelitis	[86]
		PVDF/BZT-BCT	Doping with BZT-0.5BCT ceramic nanoparticles	β-phase content of 92.1%; Output voltage of 6.37 V (at 26.5 kPa)	Ultrasonic cleaner	40 MHz US	ROS (•O_2_^−^, •OH) under US	99.86% (*E. coli*, 60 min) 98.39% (*S. aureus*, 60 min)	Vital signs monitoring	[155]
		PVDF/T-ZnO	Heterointerface engineering	Piezoelectric output voltage: PVDF/T-ZnO 0.05 V (pure PVDF 0.03 V); Bandgap: 3.16 eV	UV Point Light Source Ultrasonicator Programmed Stepper Motor	30 mW/cm^2^ light	ROS (•O_2_^−^, •OH) under US + Light	*S. aureus*, 30 min	Self-cleaning intelligent mask	[156]
		MOF@Au-DNase-I	Heterointerface engineering	Bandgap: 2.20 eV	N/A	1 W/cm^2^ light 1.5 W/cm^2^ US	ROS (•OH, ^1^O_2_) under US + NIR propulsion + eDNA hydrolysis	*S. aureus*	Deep-seated biofilm infection	[157]

∗Note: To enable cross-study comparison, antibacterial efficacy is reported under specified conditions (ultrasound parameters, material concentration, bacterial load, and matrix environment) where available. d_33_ values are as reported by original authors; measurement conditions may vary. N/A: not available.

#### 3.2.3 Rational design of piezocatalytic nanomaterials for enhanced antibacterial performance

The performance of piezoelectric nanomaterials is fundamentally determined by their crystal structure, size and morphology, crystalline phase composition, and interfacial structure. At the experimental level, coordination chemistry strategies, such as coordination assembly, template-directed synthesis, and ligand modification, enable precise control over these critical parameters, serving as essential synthetic pathways for fabricating high-performance piezoelectric nanomaterials. On the other hand, theoretical computational methods, including first-principles calculations and molecular dynamics simulations, elucidate the structure-property relationships between microscopic structure and macroscopic performance from a mechanistic perspective. These methods not only predict material properties but also provide theoretical guidance for optimizing synthesis routes, thereby establishing a collaborative research paradigm of “computation-guided design and experimental validation and optimization”. The deep integration of these two approaches will accelerate the development of piezoelectric nanomaterials with enhanced performance and controllable fabrication, advancing the field toward rational design and targeted synthesis. To better show the impact of material optimization on piezoelectric performance and related parameters, Table 4 presents some representative results.

**Table 4:** Comparison of piezoelectric performance and related parameters of piezoelectric materials based on different optimization strategies.

Representative Material	Modification Strategy	Optimized Material	Key Performance Improvements (Original Text)	Ref.
BTO	Oxygen vacancy defects (NaBH_4_ thermal reduction, 400 °C)	BTO-400	RhB degradation: ∼31% → 100%	[142]
UIO-66	Au nanoparticle deposition (in situ reduction)	UIO-66-Au	d_33_: 71 → 122 p.m./V (+71.8%)	[158]
BiOI	Heterointerface engineering with few-layered Mxene (Ti_3_C_2_) + surface oxygen vacancies	BT (BiOI/Ti_3_C_2_)	d_33_: 35.9 → 129.22 p.m./V (3.6-fold); PL lifetime: 0.314 → 3.36 ns	[54]
MoS_2_	Dual-defect engineering: sulfur vacancies + boron doping	Vs-B_3.0_/MoS_2_	d_33_: 5.6 → 35.1 pC/N (6.27-fold); PFM amplitude: 104.8 → 403.9 p.m.	[147]
ZnO NWs	Core-sheath heterojunction (ZnO NWs@TiO_2-x_N_y_) + N-doping	ZnO NWs@ TiO_2-x_N_y_	Bandgap: 3.05 → 2.81 eV; Photocurrent: 0.16 → 0.73 μA/cm^2^ (3.56-fold)	[140]
ZnO	Schottky heterojunction with graphdiyne (GDY)	ZnO@GDY	d_33_: 7.3 → 11.0 p.m./V (+50.7%); Piezocurrent: ∼6 → ∼10 nA	[154]
ReS_2_	Vacancy defects (Ar ion beam etching, 40 s)	ReS_2_@C-40	d_11_: 0 → 23.07 p.m./V (piezoelectric response triggered by defects)	[119]
Bi_4_O_5_Br_2_	Ru-doping (oxygen vacancies + lattice distortion)	Bi_4_O_5_Br_2_@Ru	d_33_: 12 → 39 p.m./V (3.25-fold); Oxygen vacancy content: 19.2% → 39.5%	[113]
P(VDF-TrFE)	Ionic liquid ([Emim][HSO_4_]) doping (20 wt%)	P(VDF-TrFE)/20%IL	β-phase content: 82.83% → 92.1% (Output voltage: 2.24 → 6.37 V)	[152]
PVDF/BaTiO_3_	Surface modification with Ag NPs (strawberry-like structure)	PVDF/4Ag-pBT	d_33_: ∼5.5 → 8.2 pC/N; Output current: ∼95 → 142 nA (+50%); Output voltage: ∼7.1 → 10 V (+40%)	[153]
ZnO	Carbon sphere (HTCS) interface (Schottky heterojunction)	ZnO@HTCS	Bandgap: 3.12 → 1.37 eV; Carrier lifetime: 1.89 → 5.35 ns; Piezoelectric field: 1.45 × 10^6^ → 2.43 × 10^6^ V/m	[159]

##### 3.2.3.1 Bandgap structure engineering

Piezocatalytic generation of ROS plays a crucial role in antibacterial therapy. However, the limited catalytic performance of most piezocatalytic materials remains a major bottleneck restricting their practical application [160]. The efficiency of piezocatalytic ROS generation is fundamentally governed by the generation, separation, and migration efficiency of electron-hole pairs. As the core electronic property of materials, the band structure directly regulates these processes: the bandgap width influences the yield of charge carriers, while the band edge positions determine their redox capacity in interfacial reactions, both collectively governing the overall catalytic efficacy [161,162]. In recent years, coordination chemistry has provided a new perspective for optimizing this process. By precisely modulating the coordination environment of the central metal ions, including ligand type, coordination number, and spatial configuration, the electron cloud distribution and orbital hybridization state of the material can be systematically adjusted, thereby enabling rational design and customization of the band structure, including both bandgap width and band edge positions [163,164].

Building on this, Wang et al. designed and constructed an intelligent wearable system based on bio-MOFs with integrated piezoelectric response and electrocatalytic functionality, guided by coordination chemistry. Through directed metal-ligand coordination, the team synthesized a Zn-Car MOF with a high piezoelectric coefficient (d_33_ ≈ 11.17 p.m. V^−1^) and a Cu-HHTP MOF with electrocatalytic activity. By combining the Zn-Car MOF with microstructured PDMS, a triboelectric-piezoelectric hybrid nanogenerator was fabricated, exhibiting a broad pressure response range (1 Pa-100 kPa) and high output voltage (131 V), enabling efficient harvesting of mechanical energy from both the environment and human motion. The system further utilizes the harvested electrical energy to drive the electrocatalytic reaction of the Cu-HHTP MOF, efficiently generating ROS (^1^O_2_ and ·OH). Coupled with antibacterial drugs loaded within the MOF pores, this synergy achieves a sterilization efficiency exceeding 98% against various pathogens, including *E*. *coli*, *S. aureus*, and *Candida albicans* (*C*.*albicans*). This work demonstrates an integrated strategy of “coordination structure design-band engineering regulation-piezoelectric-catalytic synergy-intelligent antibacterial therapy”, offering an innovative pathway for the development of next-generation high-performance, self-powered biomedical electronic systems [165].

From a coordination chemistry perspective, doping and interface design can be rationalized as controlled modifications of the local coordination environment, which alter orbital overlap, charge density distribution, and electron transfer pathways [166]. These modifications are designed to enhance charge separation, minimize recombination, and extend carrier lifetime, directly amplifying ROS production. Simultaneously, the material's morphology and lattice features are paramount, demanding concerted research to optimize these structural factors. Coordination chemistry further provides strategies for morphology control through ligand-directed growth and for lattice tuning via strain engineering induced by coordinated ion incorporation. Thus, a holistic strategy that integrates both electronic and structural engineering guided by coordination chemistry principles is vital for achieving maximum piezocatalytic efficiency [167].

**Fig. 4:**
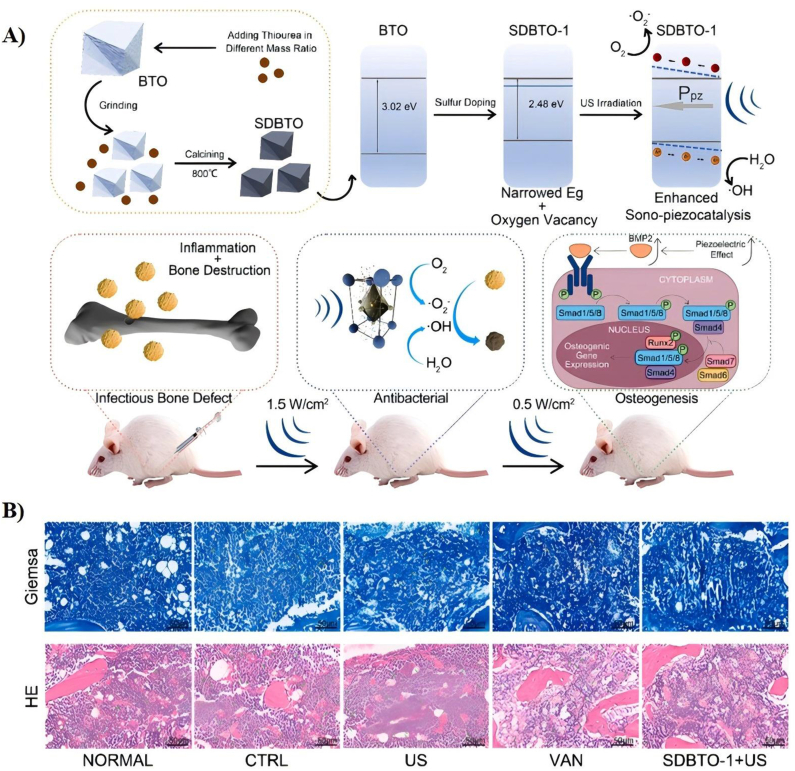
A) Piezocatalysis mechanism of antibacterial and osteogenic therapy; B) Quantified Norden score result based on Giemsa and HE staining images [174]. Copyright 2022, Elsevier.

**Elemental doping and substitution:** Doping is a key method for modifying the properties of materials to achieve desired piezoelectric characteristics for practical applications. This is accomplished by incorporating specific dopants into the base composition of piezoelectric materials. Guest molecule intercalation into the crystal lattice typically disrupts symmetry and forms polar domains, thereby amplifying the overall polarization of the doped crystal. Transition metal ions are commonly employed as dopants in this process [[168], [169], [170], [171], [172]]. From the perspective of coordination chemistry, the substitution of host cations by dopant ions alters the local coordination environment, causing changes in bond length, bond angle, and electron density distribution. Such modifications in the coordination structure may lead to lattice distortion and formation of lattice defects such as oxygen vacancies, thereby regulating the band structure and charge carrier dynamics [173]. For example, as illustrated in Fig. 4A, Lei et al. developed an ultrasound-responsive piezocatalytic system based on sulfur-doped barium titanate (SDBTO). The study thoroughly investigated the structure-activity relationship between sulfur doping levels and piezocatalytic efficiency, particularly addressing the dual demands of efficient eradication of *S. aureus* and promotion of bone regeneration. From the perspective of coordination chemistry, the introduction of sulfur not only effectively modulated the local coordination environment of the Ti-O octahedra, narrowing the material's bandgap, but also introduced oxygen vacancies into the lattice at controlled concentrations. This coordination modulation combined with defect engineering synergistically enhanced the piezoelectric properties of BTO and significantly improved the separation efficiency of piezoelectric-induced electron-hole pairs, thereby endowing SDBTO with excellent piezocatalytic activity. Specifically, the optimized SDBTO-1 achieved a 97.12% eradication rate against *S. aureus* under ultrasound activation. More importantly, the system demonstrated potential for intelligent therapy: activated by low-intensity medical ultrasound, SDBTO-1 generated mild and controllable piezoelectric signals, which could intelligently regulate the osteogenic differentiation of human bone marrow mesenchymal stem cells through specific activation of the TGF-β signaling pathway. In vivo studies further confirmed that SDBTO-1 effectively treated *S. aureus*-infected tibial bone defects in rats, significantly reducing inflammation while promoting bone regeneration alongside antibacterial action (Fig. 4B) [174]. Hu et al. developed a dual-regulation strategy that synergistically enhances the polarity and piezoelectric properties of MOF materials through amino functionalization (–NH_2_) coupled with copper ion coordination. The coordination chemistry mechanism of this strategy is as follows: the introduction of amino groups modifies the electronic structure of the organic linkers, while the Cu–N coordination bonds formed between copper ions and the amino groups in the framework further modulate the local coordination environment of the material. This coordination regulation significantly enhances the material's dipole moment (from 6.60 D to 25.99 D) and piezoelectric coefficient (d_33_ from 1.69 p.m./V to 26.21 p.m./V), thereby effectively promoting the separation of photogenerated charge carriers [175]. Furthermore, within the framework of coordination chemistry modulation, similar strategies have been applied to other systems. For instance, in the synthesized Ba^2+^-doped brookite TiO_2_ nanorods (TiO_2_:Ba), heterovalent ion doping induced lattice distortion and polarization enhancement, combined with vacancy defect engineering, synergistically optimized the material's coordination field and charge distribution, thereby significantly improving piezocatalytic ROS generation efficiency. This enhancement enabled 100% inhibition of *E. coli* even at low concentrations, reaffirming the critical role of precise coordination environment design in enhancing piezocatalysis and achieving intelligent antibacterial functionality [171].

To address the bottlenecks of hypoxic microenvironments and antioxidative defense mechanisms in the treatment of deep-seated bacterial infections using traditional sonodynamic therapy, this study developed a Ru-doped Bi_4_O_5_Br_2_ nanomaterial (Bi_4_O_5_Br_2_@Ru) with integrated piezoelectric and enzyme-like synergistic functions [110]^.^ Density functional theory (DFT) calculations revealed that Ru doping systematically enhances the sonodynamic performance of the material by modulating its electronic structure, improving oxygen adsorption capacity, and optimizing the catalytic pathway. Specifically, doping-induced lattice distortions and defect states enhance carrier separation efficiency; the synergistic effect between oxygen vacancies and Ru lowers the oxygen molecule adsorption energy from −3.188 eV to −3.506 eV, significantly promoting the generation of ROS; and the reaction energy barrier for the key catalytic step (OH∗ protonation) is reduced from +0.13 eV to −1.18 eV, transforming this process into a thermodynamically spontaneous reaction. These combined characteristics overcome the oxygen dependence of sonodynamic therapy and effectively reverse the antioxidative microenvironment at the infection site, providing a novel material design strategy for the non-antibiotic treatment of deep-seated infections.

**Constructing heterojunctions:** In the surface and interfacial engineering of piezoelectric materials, precise regulation of surface and interfacial properties based on coordination chemistry provides an intelligent and innovative strategy for the treatment of deep tissue infections [176,177]. Surface modification using functional ligands (e.g., polyethylene glycol, polydopamine) not only enhances the biocompatibility and targeting capability of the materials but also improves their in vivo stability and therapeutic efficacy through coordination interactions [178]. Meanwhile, the modulation of interface structures (e.g., constructing heterojunctions or core-shell architectures) can optimize the charge behavior under mechanical or ultrasonic stimulation, strengthen the localized electric field, and significantly promote ROS generation, thereby achieving efficient antibacterial functions [179].

The generation of ROS is key to the antibacterial process, and its efficiency is directly influenced by charge separation and interfacial reactivity. Through interfacial engineering approaches such as heterojunction construction, material polarization can be effectively enhanced, charge transfer can be promoted, and radical generation can be increased, thereby strengthening the inhibition of deep-seated pathogens. This strategy provides an important pathway for the development of intelligent and responsive anti-infection systems.reactions. For example, Wu et al. developed a barium titanate-based piezoelectric sonosensitizer, Au@BaTiO_3_ (Au@BTO) and Lei et al. developed a selenium-modified barium titanate nanoparticle (Se@BTO NPs) with antibacterial and bone regeneration capabilities [172,180]. By forming with schottky heterojunction structure, the materials facilitated the redistribution of piezo-induced charge carriers under ultrasound (US) irradiation, enhancing electron-hole pair separation, significantly improving their piezoelectric SDT efficiency.

In other recent studies, interfacial coordination engineering has been further extended to the design of complex heterojunctions. For instance, Yu et al. constructed a PCN-222-BTO heterojunction, whose interfacial integration relies on the formation of coordination bonds, and this structure exhibits excellent piezoelectric responsiveness under ultrasound activation, enhancing antibacterial activity [181]. Sharma et al. fabricated NaNbO_3_/ZnO piezoelectric nanocomposites by forming a p-n heterojunction, where the coordination environment at the interface regulates charge transport and improves catalytic and antibacterial performance [182]. Additionally, Zheng et al. developed a PLGA/Zn-KNN piezoelectric antibacterial scaffold, in which coordination synergy between ZnO and KNN enhances the piezoelectric response, achieving a combined antibacterial effect [183].

**Fig. 5:**
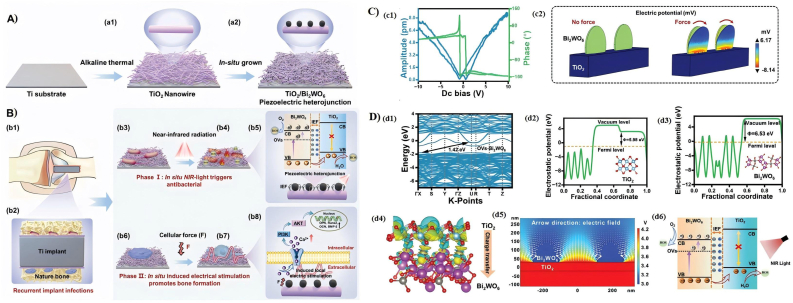
A) Schematic illustration of the in situ construction of TiO_2_/Bi_2_WO_6_ on the titanium implant surface; B) NIR light-triggered ROS (facilitated by the built-in electric field, IEF) and heat production for photodynamic and photothermal antibacterial therapy, and cellular force-induced electrical stimulation for bone formation; C) Piezoelectric properties of TiO2/Bi2WO6 heterojunctions; D) Density functional theory calculations were employed to systematically analyze TiO_2_ and Bi_2_WO_6_ for the construction of their piezoelectric heterojunction [111]. Copyright 2024,Wiley-VCH GmbH.

**Fig. 6:**
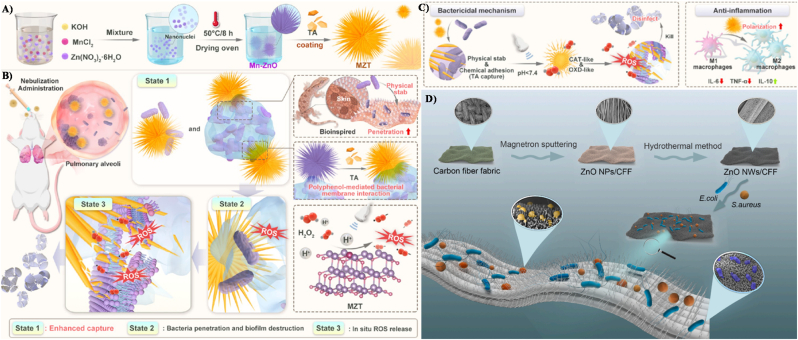
A) Design of MZT with a biomimetic cocklebur-inspired spine-like architecture for synergistic bacterial eradicationa. (1) Fabrication process of spiky MZT particles. (2) Mechanism of MZT in penetrating and disrupting antibiotic-resistant *K. pneumoniae* for the treatment of bacterial pneumonia. (3) The treatment processes and mechanisms of MZT for disintegrating the biofilm of antibiotic-resistant pathogens and treating bacterial lung infections [195]; B) Antibacterial fabrics based on synergy of piezoelectric effect and physical interaction [51]. Copyright 2025, ACS Publications; Copyright 2025, ACS Publications.

**Fig. 7:**
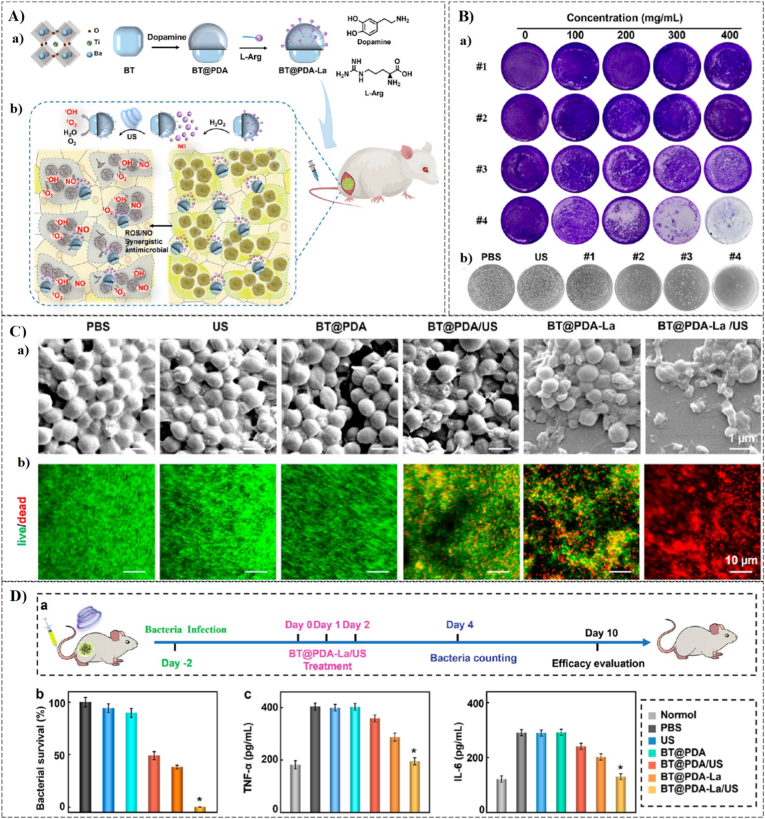
Biofilm Microenvironment-Sensitive Piezoelectric Nanomotors for Enhanced Penetration and ROS/NO Synergistic Bacterial Elimination, A)Schematic preparation process (a) and antimicrobial mechanism (b) of BT@PDA-La NMs; B) In *vitro* antibiofilm efficacy of BT@PDA-La NMs shown as photographs of crystal violet-stained MRSA biofilms (a) and bacterial survival shown as typical colony units on the plates after different treatments (b); C) SEM (a) and CLSM images of live/dead-stained bacteria after different treatments (b); D) In *vivo* treatment schedule for MRSA-infected pyomyositis with BT@PDA-La/US (a), survival rate of MRSA in infected thighs after different treatments (n = 6; ∗: p < 0.05 vs other groups) (b) and TNF-α and IL-6 (c) levels in serum after different treatments for 10 days (n = 6; ∗: p < 0.05 vs other treatments). Reproduced with permission [209].Copyright 2024, American Chemical Society. (For interpretation of the references to colour in this figure legend, the reader is referred to the Web version of this article.)

**Fig. 8:**
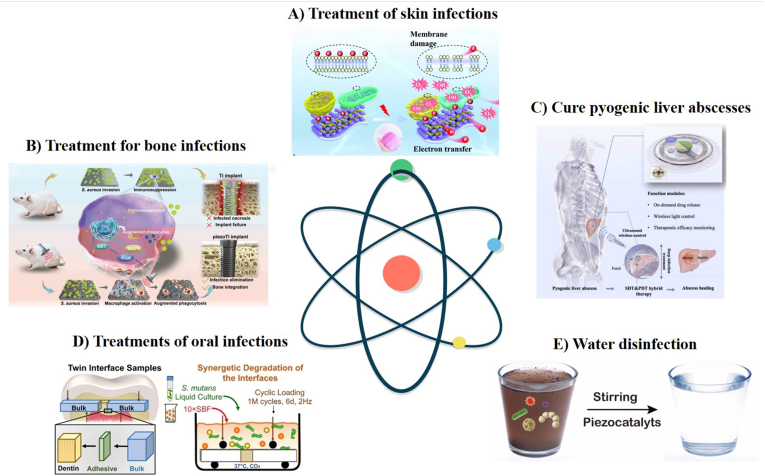
Applications of piezoelectric materials in antibacterial therapeutics [87,92,215,222,223]. Copyright 2024, Elsevier; Copyright 2021, Elsevier; Copyright 2023, Wiley-VCH GmbH; Copyright 2021, American Chemical Society; Copyright 2024, Royal Society of Chemistry.

Coordination chemistry has also been applied to regulate the electronic structure and optical properties of heterojunctions. For example, Chen et al. designed a narrow-bandgap P-bioHJ material, whose band structure was optimized through coordination modulation, resulting in a substantial increase in radical yield under cavitation-induced acoustic effects and enabling rapid intelligent sterilization [86]. In another study, Fas et al., reported advances in piezoelectric heterojunctions based on a light-cellular force-electric coupling mechanism for synergistically promoting bone regeneration and eliminating pathogenic bacteria. As shown in Fig. 5A, a heterojunction array (TiO_2_/Bi_2_WO_6_) was constructed by decorating TiO_2_ nanowires with piezoelectric nanocrystals (Bi_2_WO_6_) and fabricated as a biocompatible implant [111]. Under near-infrared light irradiation, the heterojunction simultaneously generates ROS and heat, enabling combined photodynamic and photothermal antibacterial effects (Fig. 5B). At the same time, mechanical forces exerted by stem cells growing on the implant surface can trigger the heterojunction to produce a local electric field, further promoting osteogenic differentiation and osseointegration. Through this cell-induced electric field, the system effectively suppresses postoperative infection recurrence while enhancing bone-integration efficiency, demonstrating its potential as an antibacterial bone-regenerative implant material. Compared with pure Bi_2_WO_6_, the TiO_2_/Bi_2_WO_6_ heterojunction exhibits significantly enhanced piezoelectric performance(Fig. 5C1). This is primarily attributed to the built-in electric field at the heterojunction interface, which disrupts the centrosymmetry of the material and induces polar symmetry, while oxygen vacancy defects further break the lattice symmetry and promote the formation of local polar regions. COMSOL finite-element simulations indicate that the load force (0.1-10 nN) applied by cells on the nanocrystal surface can induce material deformation and drive in situ electrical signal generation (Fig. 5C2). Density functional theory (DFT) calculations reveal that both TiO_2_ and Bi_2_WO_6_ are indirect-bandgap semiconductors(Fig. 5D). Introducing oxygen vacancies into Bi_2_WO_6_ (OVs-Bi_2_WO_6_) creates intermediate energy bands within its bandgap, predominantly derived from O 2p orbitals, which significantly reduce the energy required for electron transition, thereby improving near-infrared light utilization efficiency. Due to the lower work function of TiO_2_ compared to Bi_2_WO_6_, spontaneous electron transfer occurs at the heterojunction interface, forming an electron-depletion layer on the TiO_2_ side and an electron-accumulation layer on the Bi_2_WO_6_ side, thereby establishing a built-in electric field (IEF) directed from TiO_2_ to Bi_2_WO_6_, which facilitates the separation of photogenerated carriers. COMSOL multiphysics simulations further confirm the spatial potential distribution and electric field direction of the heterojunction. Based on the above analysis, the proposed mechanism is illustrated in Fig. 5, Fig. 6, Fig. 7, Fig. 8. Under near-infrared light excitation, the TiO_2_/Bi_2_WO_6_ heterojunction enhances the photodynamic effect through the synergy between its band structure and interface functionality, promoting ROS generation and thereby achieving dual functions of antibacterial activity and osteogenesis.

##### 3.2.3.2 Microstructural modulation and defect engineering

The microstructural, morphology, lattice features, and defect states of a material are pivotal to its piezocatalytic performance.

**Microstructure modulation:** Piezocatalytic generation of ROS plays a crucial role in advanced antibacterial therapies [13]. However, developing piezocatalytic materials that exhibit excellent performance across a broad temperature range while achieving efficient charge carrier separation remains a significant challenge. Among various piezocatalytic materials, barium titanate (BTO) has attracted considerable attention as a promising candidate for advanced oxidation processes due to its excellent ferroelectric/piezoelectric properties, environmental compatibility, tunable size and morphology, and structural simplicity [184]. A promising strategy involves modulating the band configuration through intrinsic polarization. In practical applications, achieving precise multiphase coexistence within a specific and broad temperature range is essential for enhancing piezocatalysis and promoting ROS generation. In addition to band structure regulation via piezoelectric potential, the effective spatial separation of charge carriers and the provision of driving forces for their transport are equally critical to piezocatalytic performance [[185], [186], [187]]. Key factors influencing piezocatalytic activity include high-intensity piezoelectric potential, corresponding band structure modulation, and control over internal charge conduction. To address these challenges, Huang et al. developed an environmentally friendly BTO-based perovskite ferroelectric material with tailored multiphase coexistence across a wide temperature range by introducing phase-boundary modifiers. This design enhances piezoelectric performance and increases piezoelectric potential by reducing polarization anisotropy and flattening the Gibbs free energy distribution. The enhanced piezoelectric potential in the modified BT-based piezocatalyst significantly improved its ROS generation rate under ultrasonic stimulation, reaching 3.5 times that of pure BT. Furthermore, the piezocatalyst exhibited a degradation rate constant of 0.0182 min^−1^ for dye wastewater within 30 min and achieved a 95% antibacterial rate against *E. coli*. Theoretical simulations revealed that the local distortion of TiO_6_ octahedra induced by the incorporation of Ca^2+^ and Zr^4+^ further enhanced piezocatalytic performance by promoting the spatial separation of electron-hole pairs and increasing responsiveness to minor structural deformations. This study provides valuable insights for designing high-performance piezocatalysts with efficient ROS generation capabilities, applicable in wastewater treatment and sonodynamic therapy [188].

**Oxygen vacancy engineering:** In addition to constructing heterojunctions, introducing oxygen vacancies is also an effective strategy to enhance ROS generation [189,190]. The core of this strategy lies in modulating the local structure of materials through coordination chemistry, such as introducing controllable defects into the crystal lattice via ion doping, thereby optimizing the piezoelectric and catalytic properties of the material. Taking Al^3+^-doped strontium titanate/titanium dioxide nanotubes (Al-STNT) as an example, this material has been developed as an ultrasound-responsive nanocoatings for modifying titanium implant surfaces. The introduction of Al^3+^ not only creates oxygen vacancies in the SrTiO_3_ lattice through coordination substitution but also induces lattice distortion, regulating the electronic structure and piezoelectric response of the material. Under ultrasonic stimulation, the coating leverages the piezoelectric effect to overcome the bandgap barrier, facilitating carrier separation and thereby significantly enhancing ROS production. During sonodynamic therapy, ultrasound-activated Al-STNT can generate substantial amounts of ROS on demand, achieving localized and controllable biofilm disruption and bacterial metabolic inhibition. Furthermore, owing to its surface coordination-engineered SrTiO_3_ nanostructure, the coating exhibits excellent bioactivity, promoting osteogenic differentiation and osseointegration within the alveolar bone. In rat models simulating dental implantation, Al-STNT demonstrated remarkable enhancement in the bonding strength and stability at the implant-bone interface while maintaining efficient piezocatalytic antibacterial effects. This highlights its dual functionality in intelligent responsive implant coatings: on one hand, enabling on-demand antibacterial activity through piezoelectric–chemical coupling, and on the other, promoting tissue regeneration via surface coordination modulation [168].

##### 3.2.3.3 Surface and interface engineering

**Morphological design:** Morphological regulation serves as a pivotal element in the design of piezoelectric materials for antibacterial and stimulus-responsive applications [191,192]. By integrating principles from coordination chemistry and crystal engineering, the rational design and optimization of the microstructure can not only enhance physical interactions with bacteria but also precisely regulate charge separation and transport dynamics under mechanical stimulation. This enables efficient synergistic integration of dual mechanisms: physical penetration and electrochemical catalysis. The design of specific morphological structures, such as nanoneedles, microspheres, or porous frameworks, requires adherence to principles of lattice matching and surface energy regulation to optimize bacterial capture and membrane penetration efficiency while simultaneously enhancing piezoelectric responsiveness. For example, exposing highly active crystal facets, constructing heterojunction interfaces, and modulating lattice distortion can strengthen localized polarization electric fields, promote the directional generation of ROS, and endow materials with intelligent responsiveness to stimuli such as ultrasound or mechanical stress. Thus, morphological engineering is not only a crucial strategy for achieving high antibacterial efficacy but also a theoretical foundation for developing smart and functionalized piezoelectric materials [193,194].

For instance, inspired by the natural antibacterial microstructure of gecko skin, Zhao et al. developed hybrid microparticles with burr-like microneedle topography, based on coordination-driven self-assembly, through the modification of manganese–zinc oxides with tannic acid (denoted as MZT, Fig. 6A). By coordinating metal-organic ligand interactions and regulating crystal growth, the material achieves synergistic intelligent design of morphology and chemical composition, thereby establishing a dual antibacterial mechanism. On one hand, the microneedle structure enhances bacterial membrane affinity through polyphenol-mediated coordination and, combined with physical puncture, achieves targeted bacterial removal. On the other hand, under ultrasound activation, the material exhibits responsive piezoelectric effects, forming an acid-responsive ROS catalytic system capable of intelligently switching antibacterial modes according to microenvironmental pH changes, selectively eliminating pathogens without damaging healthy tissues. Theoretical simulations further reveal that the surface piezoelectric potential can dynamically enhance catalytic reaction kinetics by regulating charge distribution and lattice polarization. In a mouse model infected with *Klebsiella pneumoniae*, the aerosol-delivered particles demonstrated excellent therapeutic efficacy. Both cell viability assays and histopathological analysis confirmed the material's favorable biocompatibility and intelligent controllability at both cellular and organismal levels, providing a new paradigm for the biomimetic design of targeted antibacterial materials while inhibiting the development of drug resistance and maintaining host microbiome homeostasis [195].

In another study, Wang et al. (Fig. 6B) developed a multifunctional antibacterial strategy based on electron transfer and mechanical interactions between ZnO lattice structures and bacteria. Addressing the fact that approximately 80% of bacterial transmission occurs via surface contact, the team grew ZnO nanowire arrays in situ on flexible carbon fiber fabric. Through regulation of ZnO lattice defects and interface band alignment, this structure effectively disrupts bacterial electron transport chains, achieving over 99.99% inactivation rates against *Staphylococcus aureus* and *Escherichia coli* within 2 min. After 10 washing cycles, the fabric retained 97% antibacterial efficiency, demonstrating excellent structural stability and reusability. Moreover, this strategy exhibited outstanding antibacterial performance across seven different fabric substrates, highlighting its universality based on lattice engineering and interface adaptation, thus providing a theoretical basis and application prospects for developing high-performance, biosafe protective clothing [51].

**Surface charge regulation:** Extensive research has confirmed that positively charged groups can disrupt negatively charged bacterial cell membranes through electrostatic interactions, thereby exhibiting significant antibacterial activity. In contrast, negatively charged surfaces can promote the adsorption of cations such as calcium ions from the surrounding environment through coordination chemistry mechanisms. This not only enhances biomineralization but also accelerates Ca^2+^ influx via coordination between calcium ions and cell surface receptors, thereby effectively promoting the osteogenic process [196]. Based on this principle, Janus membranes with asymmetric charge distributions provide an innovative strategy for guided tissue regeneration (GTR) therapy. This asymmetric charge design, founded on differences in coordination environments, simultaneously enhances antibacterial and osteogenic functions. It addresses the bone resorption issues caused by periodontitis while circumventing the technical challenges faced by traditional active ingredient-controlled release systems [197,198]. To overcome the limitations of conventional GTR membranes, which lack antibacterial and osteoinductive capabilities, researchers have developed a novel piezoelectric Janus barrier membrane. This membrane utilizes surface charges generated by piezoelectric materials under mechanical stress, with precise regulation of charge distribution based on coordination chemistry principles. Using electrospinning and subsequent processing techniques, a biodegradable PLLA-based Janus membrane (A-P(+)/PG(−)) with an asymmetric charge distribution was successfully fabricated. Its structure-function design is guided by coordination interactions: the positively charged A-P(+) surface faces the gingival tissue, removing bacteria through electrostatic adsorption and coordination effects; the negatively charged PG(−) surface covers the bone defect area, synergistically promoting bone repair by activating immunomodulatory pathways and enhancing mineral deposition through calcium ion coordination. Animal experimental results demonstrate that this Janus membrane significantly alleviates local inflammatory responses and effectively inhibits bone resorption. After 8 weeks of implantation, the bone density in the treatment group increased to 1637 ± 37 mg/cm^3^, outperforming clinically used collagen membranes lacking antibacterial functionality [199].

In summary, through the synergy of coordination chemistry-driven band structure engineering, microstructural and defect engineering, and surface and interface engineering, the piezocatalytic ROS generation efficiency of piezoelectric materials can be systematically enhanced from two dimensions: electronic structure modulation and structural optimization. This integrated strategy of “coordination structure design-band engineering regulation-piezocatalytic synergy” provides an innovative theoretical foundation and technological pathway for developing next-generation high-performance, self-powered intelligent antibacterial systems. Although theoretical calculations (eg. DFT) have been widely used to investigate structure-property relationships of materials, and machine learning has been successfully applied to the high-throughput design and screening of nanomaterials, the systematic application of machine learning in the field of antibacterial piezoelectric materials has not yet been reported. Therefore, integrating machine learning with the design of piezoelectric antibacterial materials under the guidance of coordination chemistry principles represents a promising and emerging research direction in this field.

### 3.3 Construction of physiology-responsive piezoelectric adaptive intelligent regulation systems

In the treatment of deep tissue infections, due to the strong physical barrier effect and high resistance to clearance of biofilms, single-mechanism antibacterial strategies often fail to achieve effective eradication. The primary reasons for the difficulty in clearing biofilms include: the dense extracellular polymeric matrix severely hinders the penetration of antibacterial agents, dormant bacteria within are insensitive to conventional therapies, and quorum sensing systems mediate adaptive resistance. Therefore, multiple synergistic mechanisms are required to overcome the multi-layered defenses of biofilms.The infected microenvironment significantly compromises the efficiency of piezocatalytic antibacterial therapy through multiple mechanisms. First, the dense biofilm structure not only impedes the transmission of mechanical stress and the diffusion of ROS but also causes substantial attenuation of external energy input. Second, persistent hypoxic conditions in the lesion area suppress oxygen-dependent free radical chain reactions, directly limiting the generation of highly efficient ROS. Third, high concentrations of reducing substances in the microenvironment rapidly quench the generated ROS, while acidic conditions may inactivate the catalyst surface. Additionally, bacteria readily develop adaptive resistance under sublethal stress, and the complex pathological environment hinders the effective accumulation of materials in the core infection zone [200]^.^ These physical barriers, chemical quenching, hypoxic constraints, and biological resistance collectively represent the core challenges facing piezocatalytic therapy, highlighting the urgent need to develop novel piezocatalytic material systems capable of intelligently responding to hypoxic/acidic microenvironments and actively penetrating biofilm barriers.

#### 3.3.1 pH-responsive

Bacterial-infected wound repair represents a significant clinical challenge in contemporary medicine, where conventional monotherapeutic approaches frequently exhibit limited therapeutic outcomes or undesirable adverse effects. In a recent investigation, Zhu et al., engineered adaptive antibacterial and tissue-regenerative nanocomposites (NCs) through the in situ growth of zeolitic imidazolate framework-8 (ZIF-8) on BTO substrates followed by controlled ciprofloxacin (CIP) encapsulation. This innovative therapeutic platform integrates pH-responsive drug release with ultrasound-activated piezocatalysis, enabling dynamic microenvironment adaptation at infection sites. In vitro analyses revealed that heterostructure-enhanced ROS production coupled with pH-dependent CIP release achieved remarkable synergistic antimicrobial activity (>99.9% pathogen eradication). Moreover, progressive ZIF-8 decomposition modulated piezocatalytic intensity while sustaining Zn^2+^ release, thereby coordinating antibacterial action with tissue regeneration. Finally, murine full-thickness wound models confirmed exceptional healing efficiency (99.3%) following BTO@ZIF-8/CIP NCs treatment [57].

#### 3.3.2 Hypoxic microenvironment-responsive system

O_2_ plays a critical role in the generation of ROS by activated sonosensitizers during antibacterial sonodynamic therapy (a-SDT) [201]. However, hypoxic conditions, commonly observed in clinical settings such as biofilm and deep-seated infections, significantly limit the bactericidal efficacy of a-SDT. Moreover, O_2_ consumption during SDT can exacerbate hypoxia, further reducing treatment effectiveness [202]. To address these challenges, researchers have explored strategies such as incorporating O_2_ microbubbles into a-SDT [203,204]. Drawing on advancements in sonosensitizer molecular design, Sun et al. recently addressed the hypoxic microenvironment through an innovative approach involving the redox interaction between endogenous H_2_O_2_ and catalase. They introduced an ultrasound-responsive nanozyme system designed to mitigate hypoxia-related limitations and enhance the effectiveness of a-SDT. Experimental findings revealed that this nanoenzyme system effectively and precisely eliminated deep-seated bacterial infections, offering a novel and promising strategy for sonodynamic therapy [205]^.^

Rather than devising multiple strategies to counteract hypoxia, some researchers have leveraged the hypoxic environment as a beneficial condition to develop hypoxia-targeted SDT therapies. In these approaches, hypoxic precursor drugs are activated to produce high cytotoxicity and enhance tissue oxygenation levels. Conversely, O_2_-dependent SDT can create a sufficiently hypoxic environment to activate dormant drugs. This synergy between SDT and hypoxia-activated drugs addresses their individual limitations, resulting in a highly effective combined disinfection strategy. Traditional sonodynamic and photodynamic hybrid therapies (SDT/PDT) face several challenges in treating deep organ abscesses, such as hypoxic microenvironments, difficulties in controlled drug release, limited laser penetration, and issues in monitoring therapeutic efficacy. Inspired by the aforementioned method, Chen et al. recently developed an implantable, wireless-controlled antibacterial patch equipped with modules for on-demand drug release, light regulation, and therapeutic monitoring to overcome these obstacles. This compact, self-adhesive patch delivers distinct electrical-thermal release signals for indocyanine green (ICG) and hemoglobin (Hb). Hb is released first to generate oxygen and alleviate hypoxia at the infection site, followed by ICG,which acts as both a photosensitizer and sonosensitizer to enhance antibacterial efficacy [92]. Lai et al. developed a self-activated composite dressing featuring piezoelectric and photothermal functional layers to address wound infections and deliver targeted therapy. The dressing incorporates piezoelectric poly-L-lactide (PLLA), which generates controllable ROS with antibacterial properties under ultrasound irradiation, effectively inhibiting bacterial growth at the wound site. Additionally, the dressing is modified with calcium peroxide (CaO_2_), which in the presence of catalase, converts ROS into molecular oxygen (O_2_), alleviating chronic hypoxia in infected wounds. The sustained O_2_ production promotes cell proliferation, migration, and tissue regeneration, accelerating wound healing. By integrating the photothermal properties of reduced graphene oxide (rGO), the dressing enhances wound recovery under near-infrared radiation by upregulating heat shock protein 90 (Hsp90) secretion. This multifunctional wound dressing demonstrates a user-friendly interface and holds significant potential for clinical applications in treating infected wounds [206].

#### 3.3.3 ROS-responsive system

Unlike planktonic bacteria, biofilms are complex structures composed of microorganisms and extracellular polymeric substances (EPS) that shield bacteria from the host immune system and impede the penetration of antimicrobial agents. While sonodynamic therapy (SDT) provides a precise approach to treating bacterial infections, its effectiveness is significantly reduced by bacterial biofilms, which block the entry of sonosensitizers and complicate the eradication of biofilm-associated diseases. In a recent study, Zheng et al. developed a nanomotor (Cu-tbDMSNs@Arg) capable of rapidly penetrating biofilms by simultaneously releasing nitric oxide (NO) and hydrogen sulfide (H_2_S) in biofilm microenvironments (BME) rich in hydrogen peroxide (H_2_O_2_) and glutathione (GSH). The nanomotor, containing Cu^2+^, also regulates a Fenton-like reaction to generate ROS, significantly enhancing its antibacterial efficacy and achieving a 2.47 log(10) reduction in bacterial counts. In a MRSA-infected pyomyositis mouse model, the Cu-tbDMSNs@Arg nanomotors effectively targeted and accumulated at the infection site, eliminating biofilms while exhibiting anti-inflammatory and angiogenic properties. This innovative BME-mediated NO and H_2_S dual-gas-driven nanomotor presents a promising approach for treating biofilm infections with excellent biocompatibility [102]. More recently, Zhao et al. designed asymmetric Ag-TiO_2_-L-arginine nanomotors that release NO under ultrasound stimulation, propelling them deep into biofilms. The Ag layer facilitates electron transfer, enhancing SDT, which, combined with PTT, further disrupts bacterial biofilms. In a mouse model of subcutaneous abscess, dual excitation resulted in significant bacterial reduction and accelerated tissue healing [104].

Beyond exogenous stimulus-triggered systems, a more advanced paradigm lies in “closed-loop” smart platforms that leverage infection microenvironment cues to achieve autonomous detection and on-demand therapy. In a pioneering study, Huang et al. developed a closed-loop patch (CLP) based on a bioinspired infection sensor for intelligent wound management [207]. The core innovation lies in the use of an engineered hyaluronic acid (HA) hydrogel (THgel) loaded with TiH_1_._924_ nanodots, which specifically responds to hyaluronidase (HAase), a virulence factor secreted by colonizing pathogens such as *S. aureus*. As HAase degrades the hydrogel, the capacitance of the underlying interdigital electrode decreases proportionally, enabling real-time, quantitative detection of infection before obvious clinical signs (e.g., erythema or purulence) appear. Once the signal crosses a predetermined threshold (20% of the initial value), the patch autonomously triggers the release of TiH_1_._924_ nanodots, which, upon subsequent ultrasound irradiation, generate ROS to eradicate the pathogens. Notably, this system integrates a "one-stone-for-three-birds" design: (1) infection detection via capacitance change, (2) on-demand sonodynamic therapy, and (3) promotion of wound healing through angiogenic HA fragments released during hydrogel decomposition. In a murine wound infection model, the CLP + US group achieved ∼90% wound closure within 12 days, with a healing rate 14.6-fold higher than that of untreated controls. This work exemplifies the power of a closed-loop system that autonomously senses, decides, and acts based on infection-specific enzymatic triggers, offering a compelling blueprint for next-generation smart antibacterial platforms.

The high-glucose environment in diabetic foot ulcers fosters biofilm formation, making bacterial infections particularly challenging to treat [208]. Although photo/sonodynamic therapies generate ROS to target bacterial infections, the limited penetration of sonosensitizers into biofilms, combined with the short lifetime (milliseconds) and diffusion range (nanometers) of ROS, hampers the complete elimination of biofilms and the treatment of biofilm-related infections. To address these challenges, researchers have developed nitric oxide (NO)-driven chemical nanomotors, which exhibit exceptional biofilm penetration capabilities. For instance, Zhao et al. designed NO-driven Janus nanomotors that enhance antimicrobial efficacy through synergistic ROS/NO treatment and deep biofilm penetration. In environments with elevated H_2_O_2_ levels, NO is unilaterally released from BT@PDA-La, enabling self-propelled motion and improved biofilm penetration. Under ultrasound, the piezoelectric properties of BT@PDA-La are activated, generating ROS via catalytic reactions. This ROS/NO synergy effectively disrupts biofilms and eliminates embedded drug-resistant bacteria in *vitro*. Notably, BT@PDA-La demonstrates superior biofilm penetration, effectively eradicating biofilm infections while promoting muscle healing by reducing oxidative stress, modulating inflammatory factors, and stimulating angiogenesis. This study highlights a promising strategy for enhancing the penetration of nanomotors in pathological environments and advances their potential for clinical applications in treating bacterial infections [209].

### 3.4 Design of synergistic multifunctional platforms

Faced with the limitations of traditional single-mode antibacterial strategies in treating biofilm infections, such as propensity to induce resistance, poor penetration, ineffectiveness against persister cells, and tissue toxicity, the development of multi-mode synergistic antibacterial systems capable of overcoming these bottlenecks holds critical scientific significance and clinical translational value for enhancing therapeutic efficacy and addressing the resistance crisis.

#### 3.4.1 SDT and drug synergistic multifunctional platforms

Traditional single-administration methods for treating bacterial-infected wounds often result in side effects or suboptimal efficacy. To overcome these limitations, Zhu et al. developed a multifunctional piezoelectric composite, BTO@ZIF-8/CIP NCs, for wound healing. This composite combines pH-responsive drug delivery with heterostructure-enhanced ultrasound-controlled sonodynamics, achieving synergistic antibacterial effects through enhanced ROS generation during the sonodynamic process and controlled release of ciprofloxacin (CIP) in acidic environments. Additionally, the biocompatible Zn^2+^ released from the composite balances antibacterial activity and promotes tissue repair. As a result, the multifunctional piezoelectric composite demonstrated significant antibacterial efficacy both in *vitro* and in *vivo*, while accelerating wound healing. This study offers valuable insights into the rational design of multi-stimulus-responsive nanomaterials for wound treatment [57].

#### 3.4.2 SDT and gas therapy synergistic multifunctional platforms

To address the limited penetration of sonosensitizers into biofilms, in a related study, Zhao et al. designed nitric oxide (NO)-actuated titanium dioxide Janus nanoparticles, which enhanced multimodal disruption of infectious biofilms (Fig. 7A) [104]. Biofilm clearance evaluation in *vitro* revealed that conventional BT@PDA NPs and BT@PDA/US showed limited efficacy due to EPS barrier effects. In contrast, BT@PDA-La/US demonstrated concentration-dependent biofilm disruption, achieving complete removal at 300 μg/mL (Fig. 7B and C). The therapeutic regimen is illustrated in Fig. 6D–a, consistent with the observed in *vitro* biofilm inhibition, both BT@PDA-La nanoparticles (48.9% survival rate) and ultrasound-assisted BT@PDA treatment (38.2% survival) showed substantially enhanced MRSA eradication compared to unmodified BT@PDA nanoparticles (89.7% survival; p < 0.05). Most notably, the combined BT@PDA-La/US therapy achieved near-complete bacterial clearance (0.2% residual) in infected muscle tissue, demonstrating exceptional therapeutic effectiveness against the established infection (Fig. 7D–b). Serum ELISA results further confirmed the antimicrobial efficacy of BT@PDA-La/US, showing significantly lower TNF-α and IL-6 levels in treated mice compared to other intervention groups (p < 0.05), with no statistical difference observed versus healthy controls(Fig. 7D–c).

#### 3.4.3 SDT and PTT synergistic multifunctional platforms

Photothermal antibacterial technology is a novel antibacterial strategy that utilizes photothermal materials to generate localized high temperatures or ROS under near-infrared light (NIR) to kill bacteria. This photothermal-piezoelectric synergy enables multimodal antibacterial effects with combined merits of efficacy, accuracy and adaptability, representing a breakthrough approach against treatment-resistant infections and biofilm pathologies. Ding et al. employed a synergistic therapeutic strategy by coupling the piezoelectric and pyroelectric properties of BTO nanotubes (BNT) under simultaneous ultrasound (US) and near-infrared (NIR) light irradiation to effectively treat bacterial infections in an osteomyelitis model [210].

#### 3.4.4 SDT and cascade enzymatic reactions synergistic multifunctional platforms

Cascade catalysis is the sequential series of multiple catalytic processes, commonly seen in biological systems. Nanozymes, which are promising alternatives to natural enzymes, have been developed from synthetic materials to achieve amplified catalytic activity via cascade engineering. Inspired by the advantages of cascaded engineering, Gao et al., constructed a piezoelectric/enzymatic cascade system for dual-driven catalytic eradication of multi-drug-resistant bacterial biofilms. Leveraging the piezoelectric effect and cascade enzymatic reactions, the MoSe_2_ NFs significantly reduced methicillin-resistant *S*. *aureus* (*MRSA*) bacterial load both in *vitro* and in *vivo* under low-power ultrasound stimulation [101].

#### 3.4.5 SDT and PDT synergistic multifunctional platforms

Dai et al., innovatively developed a flexible organic-inorganic composite film (P(VDF-TrFE)/ZnO/TPP) based on piezoelectric catalysis and photodynamic synergistic therapy [211]. This material enables efficient skin wounds healing under routine sunlight exposure and natural body movement conditions. Mechanistic studies revealed that the composite film significantly improves pathogenic bacteria inactivation efficiency by enhancing ROS production, promotes dense and orderly collagen growth, and increases wound healing efficiency, confirming the unique advantages of piezoelectric catalysis and photodynamic therapy in synergistic wound healing.

#### 3.4.6 SDT and macrophages

Macrophages play a crucial role in combating *S. aureus* infections, although the bacteria can evade immune responses, leading to abscess formation. Cellular adoptive transfer (ACT), an immunotherapy approach, enhances the body's immune defense by reinfusing modified or in *vitro*-activated immune cells into patients. Inspired by this, Liu et al. explored the use of piezoNPs-activated macrophages (piezoMϕ) for treating bacterial infections in immunosuppressed individuals. They discovered that piezoNPs (BTO@Au) generate ROS under ultrasound irradiation, significantly boosting the antibacterial activity of macrophages. Through ACT, the activated piezoMϕ demonstrated remarkable effectiveness in treating MRSA-induced abscesses, sepsis, pneumonia, and peritonitis, markedly improving survival rates and reducing bacterial load [212].

#### 3.4.7 Targeted multifunctional platforms

In recent years, passive targeting focuses on developing nanomaterial-based alternatives to antibiotics, whereas active targeting achieves specific recognition and precise elimination of target bacteria through the construction of surface structures endowed with biomimetic properties or biomolecular modifications. For instance, Qian et al. developed a pre-activated macrophage membrane-driven piezoelectric catalytic nano-spray agent (BTO@MMSa-system) that specifically targets infection sites and eliminates bacteria, thereby promoting the healing of infected wounds [213]. Under ultrasound (US) irradiation, the biocompatible BTO@MMSa nano-system targets infected areas, rapidly generating ROS to kill bacteria and accelerate wound healing. RNA-seq transcriptomics revealed that the antibacterial mechanism of BTO@MMSa involves disrupting bacterial membranes and inhibiting metabolic, biosynthetic, and energy processes. This targeted delivery system leverages macrophage membrane functionality for precise bacterial infection treatment, offering a promising alternative to broad-spectrum antibiotics and addressing the growing issue of antibiotic resistance.

## 4 Advanced applications and case studies

Piezoelectric material-based intelligent antibacterial therapies have been extensively explored for treating infectious diseases, demonstrating unique advantages particularly in the treatment of deep-seated infections. This section focuses on summarizing and discussing recent innovative applications of smart piezoelectric materials across various infection models, including deep tissue infections such as skin, bone, and oral sites(Fig. 8, Table 5) [87,92,215,222,223]. Through the integration of responsive design, adaptive energy regulation, and real-time feedback mechanisms, smart piezoelectric systems offer a new generation of technological pathways for precisely targeted deep infection treatment while enabling efficient biofilm removal.

**Table 5:** The application of piezoelectric antibacterial systems in the treatment of infectious diseases.

Disease types	Materials	Mechanical Source	Instrument	US Conditions	ROS	Bacteria	AE (%)	Intelligentization	Ref.
Skin infections	BTO@Au	Ultrasound	DJO-2776 sonicator	1.5 W/cm^2^, 1 MHz	•O_2_^−^ ^1^O_2_ H_2_O_2_	*E. coli, S. aureus*	99.23%, 99.94%	Mechanically responsive	[180]
	SF-MA/DA/Ag@BT	Ultrasound	N/A	1 W/cm^2^, 1 MHz	•O_2_	*E. coli, S. aureus*	99.23%, 99.96%	Mechanically responsive	[214]
	ZnO@GDY NR	Ultrasound	DM-200F	1 W/cm^2^, 1 MHz	^1^O_2_ •O_2_^−^ •OH	*S. aureus, P. aeruginosa*	100%, 100%	Mechano-chemical dual responsive	[154]
	PPy/CNT@CB/PVDF-HFP	Mechanical	Hand-held massage	N/A	H_2_O_2_ •O_2_^−^ •OH	*E. coli, S. aureus*	95%, 85%	Mechano-electrical dual responsive	[215]
	WS_2_	Illumination	N/A	120 W, 40 kHz	•OH	*E. coli, S. aureus*	93.0%, 90.2%	Light responsive	[216]
	MoS_2_	Ultrasound	N/A	1.5 W/cm^2^, 1 MHz	^1^O_2_ •OH	*S. aureus*	99.85%,	Mechanically responsive	[217]
N/A Bone infections	KV-MPs	Ultrasound	Sonoplus 190(Enraf-Nonius)	N/A	N/A	*E. coli, S. aureus*	-	Mechano-AI dual responsive	[218]
	ZnO-TiO_2-x_	Ultrasound	N/A	2.5 W/cm^2^, 1 MHz	^1^O_2_ •O_2_^−^ •OH	*S. aureus*	98.53%	Mechanically responsive	[145]
	SDBTO	Ultrasound	Intelect Mobile Ultrasound (Chattanooga 2776)	1.5 W/cm^2^, 1 MHz	•O_2_^−^ •OH	*S. aureus*	97.12%	Mechanically responsive	[174]
	HNTM-MoS_2_	Ultrasound	Intelect Mobile Ultrasound (Chattanooga 2776)	1.5 W/cm^2^, 1 MHz	^1^O_2_ •O_2_^−^	*S. aureus*	98.5%	Mechanically responsive	[132]
	ZnO@PCL/PVDF	Ultrasound	N/A	1.5 W/cm^2^, 1 MHz	H_2_O_2_ •OH	*S. aureus*	98.5%	Mechanically responsive	[219]
	BiFeO_3_/Ti_3_C_2_	Ultrasound	N/A	1 W/cm^2^, 1 MHz	•O_2_^−^ •OH	*S*. *aureus*	99.87%	Mechanically responsive	[220]
Oral infections	NaNbO_3_	Ultrasound	N/A	120W, 40 kHz	•O_2_^−^ •OH	*E. coli*	100%	Mechanically responsive	[182]
	SrTiO_3_	Ultrasound	N/A	1.5 W/cm^2^, 1 MHz	^1^O_2_ •O_2_^−^ •OH	*P. gingivalis, F. nucleatum*	80.4%, 82.1%	Mechanically responsive	[168]
	g-C_3_N_4_x/Bi_2_O_3_y	Illumination&Ultrasound	N/A	60 W, 40 kHz	·OH •O_2_^−^	*S. mutans*	63%	Mechano-light dual responsive	[221]
Liver infections	ICG + Hb	Illumination&Ultrasound	N/A	2 W, 500 kHz	·OH ^1^O_2_	*E. coli*	99.9%	Mechano-light dual responsive	[92]
Water infections	BTO@Au	Mechanical	Low-frequency hydromechanical device	80W, 40 kHz	•O_2_^−^	*E. coli, S. aureus*	99.9%, 97.1%	Low-frequency hydromechanical response	[222]

### 4.1 Treatment of skin infections

Chronic non-healing wounds pose significant health threats and economic burdens to society [215,[224], [225], [226]]. Effective wound management strategies are crucial for preventing infection and promoting healing. Studies have shown that electrical stimulation can promote cell proliferation, migration, and cytokine secretion, thereby accelerating wound healing [227]. However, traditional electrical stimulation devices are limited by poor portability and reliance on external power sources. Wound dressings based on intelligent piezoelectric materials can convert mechanical energy into continuous electrical energy, enabling self-powered electrical stimulation and providing innovative solutions for wound healing. In the design of intelligent piezoelectric materials, Huang et al. developed programmable core-shell piezoelectric microcapsules (KV-MPs) for the sequential treatment of deep infected wounds. The shell encapsulates piezoelectric material KNN, which generates ROS under ultrasonic excitation to achieve precise antibacterial effects. The core is loaded with vascular endothelial growth factor, which is released in a time-controlled manner as the shell degrades, promoting angiogenesis and tissue regeneration. This intelligent system demonstrated a programmable "first antibacterial, then healing" function in a mouse model, significantly improving the healing efficiency of deep infected wounds [218].

**Fig. 9:**
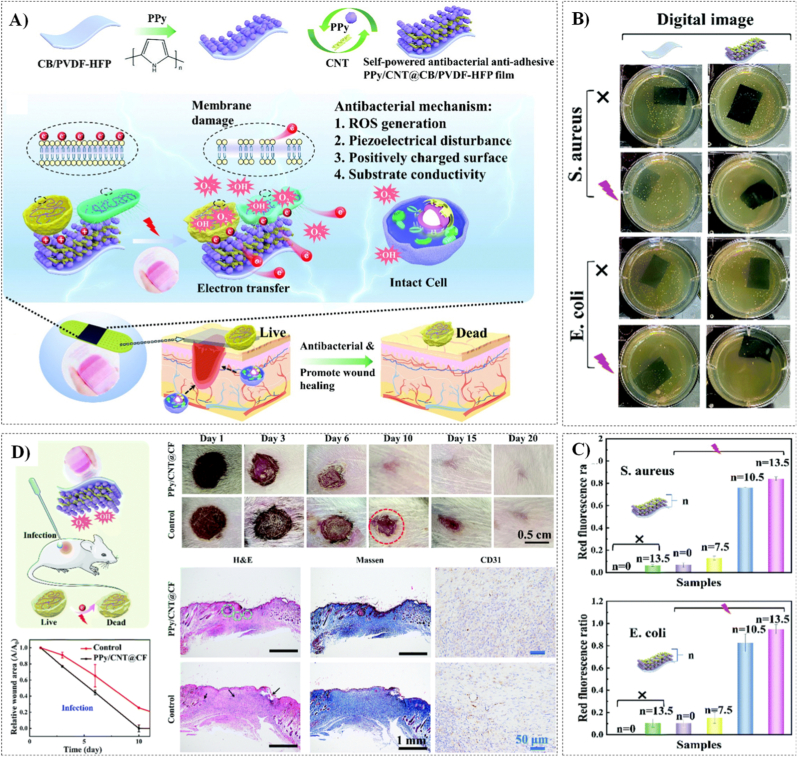
A)The preparation process and proposed antibacterial mechanism of the PPy/CNT@CF piezoelectric nanocomposite; B) In *vivo* antibacterial effects of PPy/CNT@CF against the *S. aureus*-infected wound; C) In *vitro* antibacterial effect of smooth CF and PPy/CNT@CF against *E. coli* and *S. aureus*, Reproduced with permission. [215]. Copyright 2022, Royal Society of Chemistry.

To address the challenges of treating deep tissue infections, Liu et al. constructed an ultrasound-responsive piezoelectric catalytic hydrogel. The hydrogel is embedded with barium titanate nanoparticles, which generate strong built-in electric fields and ROS under ultrasound, providing deep tissue penetration and high antibacterial activity. The hydrogel achieves self-healing and long-term bioadhesion through multiple hydrogen bonds, conforming to irregular deep wounds and significantly accelerating full-thickness healing in infected wound models [77]. Similarly, Zhang et al. developed an intelligent piezocatalytic device based on a layered polypyrrole/carbon nanotube structure on a carbon black-doped PVDF-HFP film (PPy/CNT@CB/PVDF-HFP) (Fig. 9A). This device does not require an external ultrasound source; it can be activated by mechanical pulses generated through a handheld massager to produce ROS on demand. Experimental results demonstrated disinfection efficiencies of 95% and 85% against *E. coli* and *S. aureus*, respectively (Fig. 9B and C). The device exhibited good biocompatibility and effectively promoted wound healing in a mouse model of infected wounds with only 10 min of daily mechanical stimulation (Fig. 9D). By enabling controllable antibacterial functionality through simple physical stimulation, this system provides a novel strategy for intelligent, patient-managed wound therapy [215]. Furthermore, Chen et al. utilized digital light processing technology to fabricate a 3D-printed piezoelectric catalytic hydrogel (SF-MA/DA/Ag@BT, abbreviated as SPAB) [100]. This hydrogel exhibits high uniformity, a porous structure, and piezoelectric catalytic performance. Under ultrasound exposure, it generates ROS, thereby enhancing antibacterial activity and accelerating the healing process of infected wounds [214].

To address deep infections in specific areas such as the feet, Liu's team further designed an intelligently responsive ferroelectric antibacterial insole. Under walking pressure, the insole generates a piezoelectric field and ROS, inhibiting biofilm formation and promoting fibroblast migration and angiogenesis. This provides a stable, biocompatible, and patient-adaptive therapeutic strategy for deep infected wounds [228].

Biomaterials hold unique promise for combating drug-resistant bacterial infections. However, current design strategies are largely empirical, with limited systematic guidance or predictability, making it difficult to fully harness the synergistic potential among material components. Artificial intelligence (AI) offers a powerful solution by extracting key insights from vast datasets, identifying complex underlying patterns, and enabling accurate predictive modeling. Through AI-driven design strategies, machine learning algorithms can systematically analyze the intricate relationships between material composition, structure, and performance, facilitating the rational optimization of biomaterial properties. Furthermore, artificial intelligence technology has demonstrated unique advantages in screening novel antibacterial agents [229]. For example, to address the issue of MRSA infection, this study focused on FabI, a key enzyme in bacterial fatty acid synthesis, and integrated virtual screening, machine learning (ML), explainable artificial intelligence (XAI), molecular dynamics simulations, and binding free energy calculations to identify novel FabI inhibitors from natural product databases. The research team systematically screened 41,423 drug-like polyphenolic and related phenolic compounds from the COCONUT database and ultimately obtained the optimal candidate compound, CNP0408084. This compound exhibited high binding affinity (−12.57 kcal/mol), favorable binding stability (RMSD = 1.79 Å), and high coverage (55%) of key structural features. Binding free energy calculations (ΔG_bind = −38.72 kcal/mol) and conformational analyses further confirmed its characteristics as a stable, tight-binding inhibitor. In summary, this study demonstrates the value of integrating machine learning and molecular simulation approaches in natural product drug discovery, providing a generalizable framework for prioritizing virtual screening hits based on structural features [230].

**Fig. 10:**
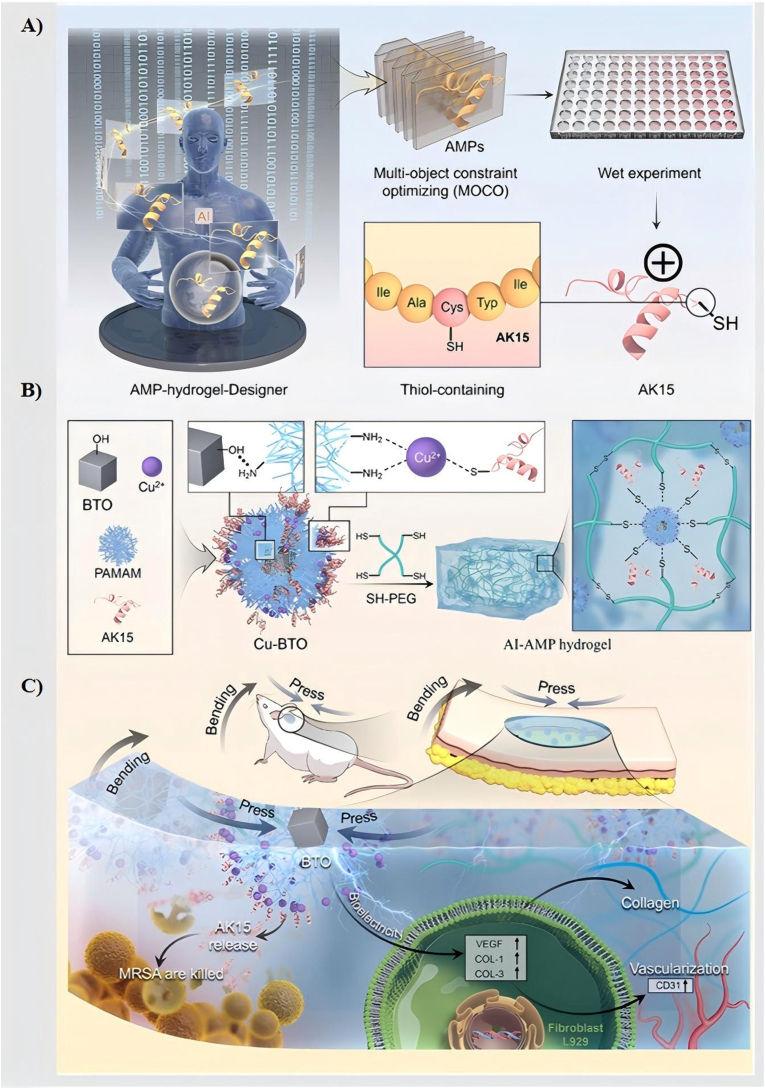
Schematic illustration of the AI-guided design and performance of the AI-AMP hydrogel. A) Generation and screening of AK15 peptide using AMP-hydrogel-Designer. B) Preparation process of the AI-AMP hydrogel. C) Application and mechanism of the hydrogel in eradicating MRSA and promoting wound healing in a rat model [231]. Copyright 2025, Wiley-VCH GmbH.

Based on the above research, as illustrated in Fig. 10, Ma et al. have developed an AI-enabled platform termed AMP-hydrogel-Designer for the automated design of AI-AMP hydrogels. The platform integrates cutting-edge computational techniques, including generative pre-training, prompt tuning, contrastive learning, knowledge distillation, and reinforcement learning. Using this approach, a novel sulfur-containing antimicrobial peptide (AK15) was generated. Its unique amino acid sequence confers potent and broad-spectrum antibacterial activity against drug-resistant bacteria. The resulting AI-AMP hydrogel acts as an integrated multifunctional system, comprising three key components that work in synergy. The hydrogel backbone, composed of PEG-4SH (four-arm polyethylene glycol alkyl ether), crosslinks with AK15 and Cu^2+^ to form a stable, injectable network. AK15 serves as an active bactericidal agent, disrupting bacterial membrane integrity and cooperating with copper ions to generate ROS, thereby enhancing antibacterial efficacy. Furthermore, copper-modified barium titanate nanoparticles (Cu-BTO) introduce mechanoelectrical transduction capability, converting mechanical energy into electrical signals that stimulate cell migration, angiogenesis, and the expression of healing-related factors. By combining AI-driven design, intelligent piezoelectric response, and efficient antibacterial repair, this platform provides an innovative intelligent solution for the treatment of drug-resistant bacterial infections [231].

In summary, intelligent piezoelectric materials convert mechanical stimuli into controllable electrical signals and chemical activity, achieving targeted antibacterial effects and programmable healing for deep infected wounds. This offers intelligent and precise novel material strategies for the clinical management of chronic non-healing wounds.

### 4.2 Treatment for bone infections

Bone tissue infections pose a significant challenge in orthopedics, particularly in complex cases such as deep osteomyelitis, which require addressing both persistent infection and bone defect repair simultaneously [232,233]. Current treatment modalities, primarily relying on antibiotics and surgery, are limited by issues such as suboptimal efficacy, the development of antibiotic resistance, and tissue damage. Therefore, the development of non-invasive, intelligent piezoelectric material systems for targeted therapy and coordinated bone regeneration in deep infected tissues has emerged as a critical research focus in this field. For the treatment of deep bone infections, researchers have developed an ultrasound-responsive piezoelectric nano-sonosensitizer coated with a red blood cell membrane. This system enhances biocompatibility through biomimetic membrane modification and generates ROS under ultrasound excitation to effectively clear bacterial infections in osteomyelitis sites. Metabolomic analysis further reveals that this intelligent piezoelectric system can systematically regulate bacterial metabolic pathways by interfering with tryptophan metabolism, enhancing oxidative stress, and disrupting nucleotide turnover, among other mechanisms [132].

To address deep infections associated with implants, researchers have further developed a spatiotemporally programmable piezoelectric-chemodynamic cascade catalytic nanoreactor. This system integrates piezoelectric barium titanate with a polydopamine-copper composite interface, enabling directional transfer of piezoelectric charge carriers and Cu^2+/^Cu^+^ cyclic catalysis under ultrasound triggering, synergistically enhancing ROS generation. Combined transcriptomic and metabolomic analyses demonstrate that this system can efficiently clear deep biofilm infections by inducing a “copper death-like” bacterial cell death mode and disrupting the tricarboxylic acid cycle [234]. Additionally, Li et al. designed a titanium implant surface functionalization system with a metal-piezoelectric heterostructure. Under ultrasound activation, this intelligent surface not only directly kills bacteria and suppresses biofilm gene expression through piezodynamic effects but also activates macrophage immune responses, enhancing their phagocytic and antibacterial functions. In an osteomyelitis animal model, a single ultrasound treatment effectively controlled deep infection and promoted osseointegration, demonstrating an integrated “therapy-immunity-regeneration” intelligent regulatory strategy [87]. In recent years, piezoelectric heterojunction implants with photo–force–electric coupling have provided novel intelligent application scenarios for the treatment of bone infections. For example, researchers have constructed a TiO_2_/Bi_2_WO_6_ piezoelectric heterojunction array on the surface of titanium implants. This system exhibits NIR light responsiveness and cell-force-to-electricity conversion capability. Under NIR irradiation, the heterojunction facilitates the separation of photogenerated carriers via a built-in electric field, synergizing photothermal and photodynamic effects to efficiently eradicate bacteria. Simultaneously, mechanical forces exerted by stem cells seeded on the implant trigger the heterojunction to generate a localized electric field, further promoting osteogenic differentiation and osseointegration. This intelligent system demonstrates excellent stage-specific antibacterial and bone-regenerative performance in a rat model of infected bone defects, achieving chronologically coordinated “antibacterial–osteogenic” therapy [179].

In the context of coordinated bone defect repair and infection treatment, researchers developed a ZnO@PCL/PVDF nanofiber scaffold with piezoelectric properties and an oriented structure. Under ultrasound stimulation, this scaffold mimics the physiological electrical microenvironment, simultaneously exerting antibacterial, immunomodulatory, and osteogenic effects. Animal experiments confirmed its ability to effectively inhibit deep bacterial colonization, reduce inflammatory responses, and accelerate bone defect healingtreatment [219]. Similarly, an ultrasound-responsive system based on a BiFeO_3_/MXene ferroelectric heterojunction, leveraging synergistic polarization fields and sonoluminescence mechanisms, achieved over 99% bacterial clearance in deep tissues within 20 min, showcasing highly efficient spatiotemporally controllable therapeutic capabilities [235].

In summary, intelligent piezoelectric materials, by converting external mechanical/acoustic stimuli into controllable electrical fields, ROS generation, and immune regulatory signals, offer innovative solutions with spatiotemporal precision, programmable responsiveness, and multi-mechanism synergy for the treatment of deep bone tissue infections, demonstrating promising clinical translation potential.

### 4.3 Treatments of oral infections

Despite nearly seven decades of development, dental composite restorations still exhibit limited clinical longevity [168,182,221,236,237]. The primary cause of restoration failure is the degradation of the bond at the tooth-biomaterial interface due to chemical, biological, and mechanical factors. Oral biofilms form at these interfaces, producing enzymes and acids that demineralize hard tissues and degrade the composite. Addressing this issue requires strategies to remove bacteria from bonded interfaces and remineralize marginal gaps, thereby extending the clinical service of restorations. To address this, Montoya et al. incorporated piezoelectric barium titanate nanoparticles as multifunctional bioactive fillers into dental resin composites, developing a piezoelectric composite material that integrates dual capabilities of intelligent antibacterial action and remineralization promotion [236]. Under the natural stress of oral mastication, this material spontaneously generates surface potentials and ROS, achieving on-demand in situ clearance of interfacial biofilms (with biofilm reduction up to 90%). Simultaneously, it induces the formation of a dense calcium phosphate mineralization layer, enhancing interfacial sealing. The researchers further pioneered a method to evaluate adhesive interface strength under synergistic effects of bacteria and cyclic loading, validating the long-term functional stability of this material in simulating the complex deep oral microenvironment.For the treatment of deep mucosal tissue infections, Badaraev et al. fabricated nonwoven piezoelectric membranes from vinylidene fluoride-tetrafluoroethylene copolymer via electrospinning and deposited an intelligent responsive copper coating on the surface using magnetron sputtering [238].

Under micro-stresses generated by oral movement, these membranes release electrical signals, synergizing with the controlled release of copper ions to dual-regulate bacterial clearance and tissue regeneration processes. In vivo experiments confirmed that this piezoelectric membrane can directionally accelerate the healing of deep oral mucosa, demonstrating the advantages of stress-driven intelligent therapy.Further addressing the prevention and treatment of deep peri-implant infections, Xu et al. designed a surface-confined metal/piezoelectric nanostructure, embedding it into the surface of polymer implants to construct an ultrasound-responsive intelligent piezocatalytic system. Upon ultrasound triggering, this system generates local electron discharge and oxidative stress at the implant-bacteria interface, effectively inhibiting the activity of deeply adherent *S. aureus* by disrupting bacterial membrane structures and depleting energy metabolic resources, while maintaining excellent biocompatibility. In an ex vivo human tooth model, this system was applied as a dental cement and successfully achieved deep and precise treatment of root canal reinfection through simple ultrasound stimulation [237].

In conclusion, smart piezoelectric materials, by transforming either the natural mechanical stimuli of the oral cavity or external ultrasonic energy into controllable electrochemical signals and antibacterial activity, offer in situ, on-demand, and programmable novel therapeutic strategies for infections at dental restoration interfaces, deep mucosal tissues, and peri-implant sites. This highlights their promising potential in the management of deep-seated oral infections.

### 4.4 Cure pyogenic liver abscesses

**Fig. 11:**
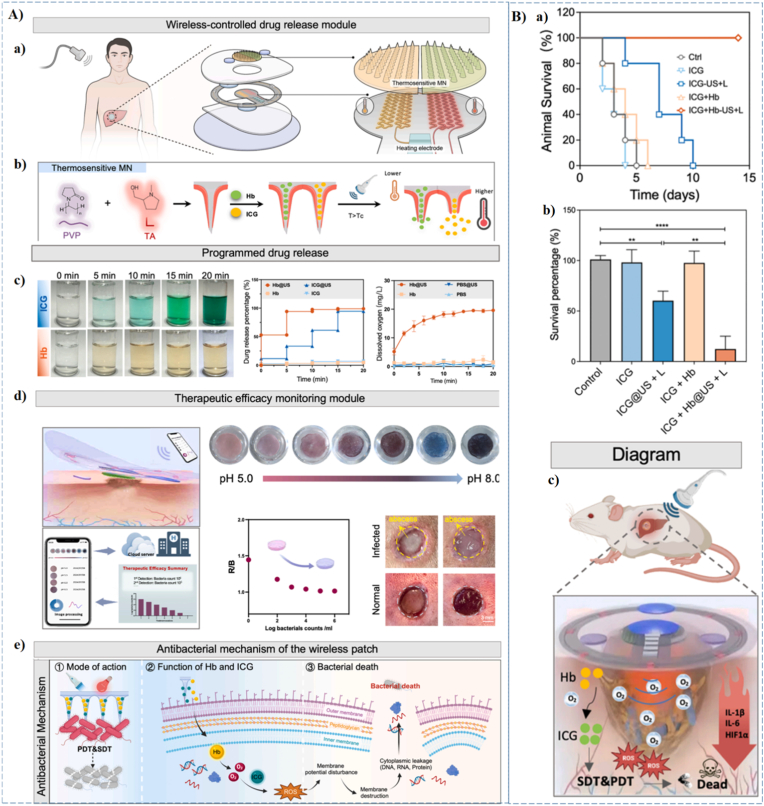
Design, structure and application of the implantable antibacterial patch for wireless SDT/PDT against PLA. A) Schematic diagram of the wireless thermal-controlled drug release module(a); The manufacturing process and drug release mechanism of the thermosensitive microneedles(b); Programmed drug releasing process of ICG and Hb from the wireless antibacterial patch and oxygen releasing levels of different groups when activated by ultrasound (c); Schematic diagram and results of the wireless antibacterial patch for monitoring PLA treatment efficacy(d); Schematic diagram displaying the antibacterial mechanism of ICG + Hb-loaded wireless patch powered by ultrasound(e); B) In *vivo* therapeutic effects of different patches for PLA in SD rats: c Survival curve of the infected rats after different treatments(a); The survival percentage of *E.coil* after different treatments(b); Schematic diagram of possible therapeutic mechanism of the wireless antibacterial patch for PLA(c). Reproduced with permission [92]. Copyright 2024, Elsevier.

Traditional sonodynamic/photodynamic combination therapies face challenges in treating deep tissue infections, including inadequate drug release control, limited light penetration depth, hypoxic microenvironments, and lack of therapeutic feedback. To address these issues, Chen et al. developed an integrated wirelessly controlled antibacterial patch with on-demand drug release, light-controlled synergistic therapy, and real-time efficacy monitoring capabilities, offering an innovative solution for the treatment of deep liver abscesses (Fig. 11A) [92]. The core of the patch is a wireless power supply and intelligent control system based on a piezoelectric transducer (Fig. 11A–a). The thermally responsive microneedles are fabricated by blending polyvinylpyrrolidone with hemoglobin (Hb) and indocyanine green (ICG), followed by coating with a phase-change material (tannic acid). This coating exhibits temperature-dependent melting and release properties, enabling precise drug release only when the phase transition threshold is reached (Fig. 11A–b). Under ultrasound excitation, the electrical energy converted by the piezoelectric effect triggers the programmed melting of the microneedle coating, achieving temporally controlled release of Hb and ICG. Scanning electron microscopy images clearly document the intelligent structural deformation of the microneedles (Fig. 11A–c).Intelligent therapeutic synergy and real-time feedback are the standout advantages of this system. The infection-responsive hydrogel integrated into the patch dynamically reflects therapeutic outcomes, supporting real-time clinical adjustments. In a pyogenic liver abscess model, the patch achieved complete infection clearance (Fig. 11A–d), validating its broad therapeutic potential for deep tissue infections. Through the sequential release and synergistic action of Hb (an oxygen carrier) and ICG (a dual-mode photosensitizer/sonosensitizer), the system simultaneously alleviates hypoxia in the lesion and initiates combined sonodynamic/photodynamic therapy (Fig. 11A–e). This self-adhesive miniaturized device conforms to deep tissues and is remotely controlled wirelessly via external ultrasound. The piezoelectric ceramic-driven drug release module generates differentiated electrothermal signals for Hb and ICG, while ultrasound activates miniature LEDs to initiate synergistic sonodynamic/photodynamic therapy (Fig. 11B). Notably, the ICG + Hb@US + L treatment group achieved a 100% survival rate in infected rat models, significantly outperforming other control groups (Fig. 11B–a). Bacterial activity assays demonstrated that the system achieved over 99.9% clearance of *E. coli* (Fig. 11B–b), primarily attributable to localized high-concentration drug delivery via the microneedles (Fig. 11B–c).

### 4.5 Anti-infective therapy for damaged gastrointestinal (GI) organs

Endogenous bacterial infections arising from compromised gastrointestinal (GI) tract integrity can induce systemic inflammatory cascades, potentially leading to severe sepsis and life-threatening complications [239]. To address this issue, Zhao et al. developed an intelligent piezoelectric implant patch based on highly oriented PVDF-TrFE nanofiber membranes, designed to modulate the electrical microenvironment of biofilms surrounding deep gastrointestinal perforation wounds. Under external ultrasound stimulation, the implant releases pulsed charges that effectively penetrate bacterial biofilms, disrupt their membrane protein structures, and interfere with the electron transport chain, thereby inhibiting bacterial proliferation. Through multi-omics analysis and **molecular dynamics simulations**, the study elucidated the mechanism by which intelligent piezoelectric charges achieve efficient antibacterial effects by disrupting the macromolecular structure of biofilms and interfering with energy metabolism pathways. In a rat cecal ligation and puncture model, the combined application of the patch and ultrasound therapy significantly reduced the degree of intra-abdominal infection, suppressed the systemic spread of bacteria, and alleviated systemic inflammatory responses. This research provides a wireless, controllable, and intelligent piezoelectric therapeutic strategy for deep endogenous infections [239].

## 5 Challenges and future directions

Recent advancements in antibiotic-free therapies have shown significant promise in preventing and controlling multi-drug-resistant (MDR) bacterial infections. In the field of biomedicine, interdisciplinary approaches to antibacterial therapy have emerged as a cutting-edge research frontier. Among these, antimicrobial piezocatalytic therapy has garnered considerable attention from researchers and clinicians due to its potential as a noninvasive treatment for hard-to-reach infections. Piezocatalytic antimicrobial therapy relies on a highly efficient and biocompatible advanced oxidation process, initiated by mechanical energy, to catalyze the generation of reactive species, particularly ROS. This process involves interactions between piezoelectric nanomaterials and chemical substances, such as oxygen and water molecules, and has demonstrated efficacy in treating various conditions, including wound sterilization, periodontitis, and bone repair promotion.

This review outlines the principles of piezocatalysis and its diverse applications in antimicrobial piezocatalytic therapy, focusing on the generation of ROS for treating wound, bone, liver and oral infections, as well as water disinfection. It aims to serve as a valuable reference for designing piezocatalytic materials in antibacterial nanomedicine and to advance the research and clinical translation of nanocatalytic medicine. The pharmaceutical industry thrives not on a single technology but on the integration of multiple disciplines to drive innovation. Piezocatalytic medicine, intersecting materials science, chemistry, biomedicine, and physics, holds vast potential for diverse biomedical applications. Although significant progress in designing piezocatalytic antibacterial systems has been made in recent years, this emerging field is still in its early stages and faces significant challenges in development and clinical implementation. As discussed below, translating these into complex application scenarios still faces multiple challenges and opportunities, which are summarized in Table 6.

**Table 6:** Summary of key design principles, major application scenarios, and remaining translational bottlenecks in piezocatalytic antibacterial therapy.

Key Design Principles	Major Application Scenarios	Translational Bottlenecks & Challenges
➢ High piezocatalytic ROS generation: • High piezoelectric coefficient (d33) • •Narrow bandgap for ultrasound response • Surface defect/vacancy engineering	➢ **Wound infections:** • Acute wounds • Chronic ulcers • Diabetic wounds ➢ **Bone & implant-related** infections**:** • Osteomyelitis • Peri-implantitis ➢ **Oral & periodontal infections:** • Periodontitis, • Peri-implant mucositis ➢ **Deep-seated & visceral infections:** • Liver abscess • Abdominal • peritoneal infections ➢ **Others**	➢ **Mechanism understanding:** • Unclear in vivo bio-effects of piezocatalysis • Lack of atomic-scale charge transfer pathways
➢ Biocompatibility & biosafety: • Hemolysis rate <5% • Low immunogenicity (IL-6, TNF-α) • Controlled degradability (ideal)		➢ **Methodological inconsistencies:** • No standardized ROS detection protocol • Ununified piezoelectric characterization (d33, surface potential) • Variable ultrasound parameters (MHz, W/cm^2^, duty cycle)
➢ **Ultrasound responsiveness & adaptability:** • Tunable frequency/intensity • pH/bacterial concentration response • Adaptive energy control (reinforcement learning)		➢ **Preclinical models gap:** • Oversimplified animal models (acute vs. chronic biofilm) • Lack of implant-related/long-term infection models • Poor translation predictability
➢ **AI-driven material design & optimization:** • GNN prediction of d33 & bandgap • High-throughput virtual screening • Closed-loop design-validation-iteration		➢ **Clinical translation & manufacturing:** • Poor batch-to-batch consistency • Unknown long-term in vivo fate (>6 months) • No GMP-compliant production pipeline
➢ **Intelligent system integration:** • Sensing-decision-response capability • Real-time microenvironment monitoring ● IoT & AI hub for full-cycle management		➢ **Regulatory & standardization gaps:** • No minimum reporting standard for ultrasound therapy • Lack of ex vivo ROS quantification SOP • No dedicated safety panel for non-degradable piezoceramics (e.g., BTO)

Beyond the aforementioned technical bottlenecks, several common methodological issues urgently require community-wide attention: (1) the lack of standardized protocols for in situ and in *vivo* ROS detection makes quantitative comparison across different studies difficult; (2) the absence of unified characterization benchmarks for piezoelectric performance (e.g., piezoelectric coefficient d_33_, surface potential) hinders fair evaluation of different material systems; (3) significant discrepancies exist between current animal models (e.g., acute subcutaneous infection, superficial wound infection) and real-world clinical infectious processes (e.g., chronic biofilm-associated infections, implant-related infections), limiting the translational predictability of preclinical findings. Addressing these common challenges will be critical for advancing the field towards clinical relevance [229,240].

**Material design integrated with machine learning guidance:** Piezoelectric semiconductor materials have proven to be effective sonosensitizers due to their physical stability, hydrophilicity, and ultrasound-responsive properties, playing a crucial role in sonodynamic therapy. Their ability to generate ROS and biocompatibility are key areas of focus for clinical translation. Current optimization strategies, such as material modification and combination therapies, primarily aim to enhance ROS generation efficiency. However, the quantitative relationship between ROS efficiency, sonosensitizer dosage, and ultrasound parameters remains unclear. Despite this, preclinical studies have demonstrated the promising potential of piezoelectric sonosensitizers in antibacterial therapy. It is noteworthy that machine learning approaches offer new pathways to address these challenges. Specifically, graph neural networks (GNNs) provide a powerful paradigm for piezoelectric crystal structure prediction. By learning atomic-scale representations from known piezoelectric materials such as perovskites and wurtzite-type compounds, GNN models can predict key properties including the piezoelectric charge coefficient (d_33_) and bandgap for millions of hypothetical compositions. This capability enables virtual screening of high-performance sonosensitizers prior to experimental synthesis. Firstly, by constructing predictive models linking material composition, structure, and performance, candidate sonosensitizers with optimized piezoelectric coefficients and band structures can be efficiently screened. Data-driven models can analyze the nonlinear mapping between ultrasound parameters (frequency, intensity, duration) and ROS yield, enabling precise pre-optimization of treatment protocols. Additionally, through adversarial sample generation and transfer learning, the kinetic behaviors of materials in complex biological environments can be simulated even with limited experimental data. In the future, integrating high-throughput computing, automated experimentation, and closed-loop learning frameworks may establish an intelligent material development paradigm of “design-validation-iteration”, systematically advancing the clinical translation of piezoelectric sonosensitizers. Deepening the understanding of SDT mechanisms and sonosensitizer pharmacokinetics, combined with rationally designed approaches driven by machine learning, will be a critical direction for the advancement of this field.Material.

**Intelligence-enabled mechanism research:** Piezocatalytic nanomaterials demonstrate broad application prospects in the field of medical science and technology. Although existing studies have employed various advanced techniques to explore their catalytic mechanisms, the specific biological effects and modes of action within living organisms remain unclear. Current research often attributes medical outcomes to the catalytic effects of materials or electric fields generated by surface charges. However, the direct impacts of nanomaterials themselves and their piezocatalytic activity on cells, tissues, and organs, as well as the underlying mechanisms, require systematic elucidation. This understanding is crucial for achieving breakthroughs in piezocatalytic medicine. It is noteworthy that intelligent approaches are providing new perspectives for mechanism research in this field. Beyond static analysis, reinforcement learning provides a transformative paradigm for closed-loop control of multi-modal energy. An RL agent can autonomously learn optimal sequences of ultrasound parameters (frequency, intensity, duty cycle, pulse pattern) through real-time interaction with the infection microenvironment, thereby balancing maximization of ROS generation against minimization of off-target tissue damage. This adaptive energy control strategy can substantially outperform conventional fixed-protocol ultrasound treatments. For example, it may initially employ high-frequency bursts to rapidly generate ROS, subsequently switching to low-frequency pulses to sustain membrane disruption [241]. By integrating multi-omics data with machine learning models, correlation networks linking “material properties-biological responses” can be constructed to systematically analyze the effects of piezocatalytic processes on cellular signaling pathways, metabolic networks, and gene expression mechanisms. With the aid of artificial intelligence-assisted molecular dynamics simulations and quantum chemical calculations, the interfacial behaviors and charge transfer pathways of piezoelectric materials in biological microenvironments can be revealed at the atomic scale. Furthermore, combining intelligent sensing and real-time imaging technologies enables dynamic monitoring and mechanistic inversion of piezocatalytic processes in vivo. In the future, establishing a closed-loop research system that integrates computational simulations, intelligent sensing, and adaptive experimentation will advance piezocatalytic medicine from phenomenological observation to mechanistic understanding, providing a theoretical foundation for precision medicine.

**Intelligent empowerment of biosafety assessment and clinical translation strategies for piezocatalytic materials:** The clinical translation of piezocatalytic materials requires systematic resolution of two core challenges, biosafety and therapeutic efficacy optimization, through intelligent approaches. By leveraging artificial intelligence models and big data analytics, material toxicity can be efficiently predicted, metabolic pathways simulated, and treatment parameters such as ultrasound dosage intelligently optimized, thereby overcoming the efficacy limitations of conventional conservative protocols. Simultaneously, real-time dynamic tracking of in vivo material distribution, retention, and long-term biological fate is enabled through intelligent imaging and biosensing technologies, providing precise safety assurance, particularly for non-degradable materials. Establishing an intelligent closed-loop system that integrates material design, safety validation, and treatment implementation is essential to advancing this technology toward safe and efficient clinical applications.

**Intelligent empowerment of scalable production and standardized manufacturing:** It is imperative to develop and standardize facile, large-scale preparation techniques for high-performance piezocatalysts to accelerate their clinical translation. Key technical bottlenecks that need to be addressed include ensuring batch-to-batch consistency, process reproducibility, and precise control over material physicochemical properties. Furthermore, a standardized evaluation system encompassing both fundamental characteristics and piezocatalytic performance of nanomaterials should be established to provide technical support for the industrial application of nanocatalysts. Typically, by harnessing the core capabilities of artificial intelligence (AI), including big data analytics, pattern recognition, and predictive optimization, we can overcome critical bottlenecks in piezocatalysis research, such as high experimental costs, poorly understood mechanisms, and suboptimal efficiency. This strategy allows for the precise prediction of material properties and the intelligent control of catalytic processes, ultimately transforming traditional research paradigms and paving the way for full-scale, intelligent advancement in the field.

**AI-driven evolution of piezocatalytic antibacterial systems:** The integration of artificial intelligence (AI) technology is driving the evolution of piezocatalytic antibacterial products from static materials into intelligent systems, enabling a paradigm shift toward intelligence and diversification. At the intelligence level, AI endows products with sensing-decision-response capabilities: by analyzing microenvironmental parameters (e.g., pH, bacterial concentration) in real time, they achieve on-demand activation and precise antibacterial action; through adaptive algorithms, they optimize piezoelectric output to maintain high catalytic efficiency under varying physiological conditions; and leveraging predictive models, they enable early intervention, transitioning from treatment to prevention. At the diversification level, AI facilitates tailored solutions for multiple scenarios: generative models and high-throughput screening allow the customization of material properties for specific pathogens and application sites; combined with smart manufacturing processes, products with diverse forms and functions, such as bone implants and smart wound dressings, are developed; and coordinated integration of piezocatalysis with other therapies enables personalized combination treatment strategies. Ultimately, these intelligent units will form a closed-loop diagnostic and therapeutic system, where data exchange and strategy optimization are achieved via the Internet of Things, and an AI hub enables dynamic early warning, precise intervention, and full-cycle intelligent management of infectious diseases, marking the advent of the intelligent era in antibacterial therapy.

**Toward Clinical Translation:** In terms of biosafety assessment, evaluating the long-term safety of nanomedicines requires the establishment of a systematic experimental framework. The core component is a 6- to 12-month repeated-dose toxicity study in both rodent and non-rodent animal models, during which general physiological status, hematological parameters, serum biochemistry (indicators of liver, kidney, and cardiac function), and histopathological changes in major organs are regularly assessed throughout the dosing and recovery phases. Given the unique properties of nanomaterials such as barium titanate (BTO), particular attention must be paid to their long-term biodistribution, accumulation, and clearance kinetics in organs rich in the mononuclear phagocyte system (e.g., liver and spleen). Their physicochemical stability under physiological conditions, as well as their degradation and drug release behavior, should be determined. Concurrently, immunogenicity (inflammatory cytokines IL-6, TNF-α), hemocompatibility (hemolysis rate <5%), and complement activation risk should be evaluated. When toxicity signals are detected, further mechanistic investigations at the cellular level are required, including studies on cell-material interactions, intracellular distribution, oxidative stress levels (ROS, GSH, MDA), and modes of cell death. In addition, three-dimensional organoid models can be employed for in vitro repeated-dose toxicity screening, and physiologically based pharmacokinetic (PBPK) modeling can be used to predict long-term in vivo behavior, thereby enabling a comprehensive assessment of the chronic safety risks of nanomedicines.

Regarding the harmonization of therapeutic protocols, the current lack of standardization in ultrasound parameters makes cross-study comparison virtually impossible. Therefore, the field should adopt a minimum reporting standard for ultrasound therapy, which must clearly include frequency (MHz), intensity (W/cm^2^), duty cycle (%), treatment duration, and waveform (continuous or pulsed). For ROS detection, a standardized operating procedure (SOP) for ex vivo ROS quantification in infected tissues should be established and widely promoted, utilizing validated probe panels such as dihydroethidium for •O_2_^−^ and hydroxyphenyl fluorescein for •OH. Finally, in the realm of scalable manufacturing, the transition from lab-scale synthesis to Good Manufacturing Practice (GMP)-compliant production must address batch-to-batch reproducibility in key material properties including size, morphology, and piezoelectric coefficient. The convergence of nanomedicine with AI and computational science offers transformative opportunities to overcome these barriers. AI-driven algorithms, including machine learning, deep learning, and generative models, are increasingly being applied to optimize nanoparticle design and synthesis, predict nanobio interactions, and enhance diagnostic and therapeutic outcomes. These approaches not only accelerate material discovery but also enable data-driven, adaptive nanotheranostic systems that can autonomously optimize across diverse disease contexts.

## 6 Conclusion

In summary, piezoelectric catalytic materials have demonstrated multidimensional application prospects in the field of nanomedicine. Beyond their use in disinfection and sterilization, they have shown remarkable efficacy in the treatment of critical diseases, wound and medical device sterilization, neural regeneration, bone repair, and biosensing, and will continue to play an indispensable role in the future. However, despite the significant development opportunities in this field, several key scientific challenges and technical bottlenecks remain, urgently requiring breakthroughs. With the in-depth study of structure-property relationships guided by coordination chemistry, coupled with the deep integration of intelligent technologies and the establishment of AI-enabled systems, particularly the rapid advancement of nanocatalytic technologies and the continuous emergence of novel functional materials, piezocatalytic medicine is poised to expand innovative treatment modalities through precise molecular design, data-driven intelligent optimization, adaptive therapeutic strategies, and controllable biological response mechanisms. This will accelerate the clinical translation process, inject new momentum into the development of modern medicine, and ultimately contribute to global healthcare.

**Table:** Abbreviations:

Abbreviation	Full Name
•O_2_^−^	Superoxide anion free radical
•OH	Hydroxyl radical
^1^O_2_	Singlet oxygen
2D	Two dimensional
*B. subtilis*	*Bacillus subtilis*
BP	Black phosphorus
BTO	Barium titanate (BaTiO_3_)
*C. albicans*	*Candida albicans*
CB	Conduction band
CDT	Chemodynamic therapy
*E. coli*	*Escherichia coli*
GSH	Glutathione
MDR	Multi-drug resistance
MOFs	Metal-organic frameworks
MRI	Magnetic resonance imaging
NIR	Near infrared
*P. aeruginosa*	*Pseudomonas aeruginosa*
PDT	Photodynamic therapy
PFM	Piezoresponse force microscopy
PTT	Photothermal therapy
QDs	Quantum dots
ROS	Reactive oxygen species
*S. aureus*	*Staphylococcus aureus*
SDT	Sonodynamic therapy
TME	Tumor microenvironment
US	Ultrasound
VB	Valence band

## Declaration of generative AI and AI-assisted technologies in the manuscript preparation process

In the preparation of this manuscript, the authors employed “DeepSeek” for the purpose of linguistic refinement and to enhance the native fluency of the English expression. Following the use of this tool, the authors thoroughly reviewed, edited, and approved the final content, assuming full responsibility for all aspects of the published work.

## CRediT authorship contribution statement

**Jiaqi Liu:** Data curation, Formal analysis, Investigation, Methodology, Validation, Writing – original draft. **Jun Li:** Data curation, Investigation, Methodology, Project administration, Supervision, Validation, Writing – review & editing. **Huiyan Sun:** Formal analysis, Methodology, Resources, Validation. **Jungmok Seo:** Formal analysis, Methodology, Supervision. **Yanmei Zhang:** Conceptualization, Formal analysis, Funding acquisition, Project administration, Supervision, Writing – original draft, Writing – review & editing. **Yunlong Yu:** Formal analysis, Funding acquisition, Investigation, Software, Supervision, Writing – review & editing.

## Declaration of competing interest

The authors declare that they have no known competing financial interests or personal relationships that could have appeared to influence the work reported in this paper.

## Data Availability

No data was used for the research described in the article.

## References

[1] Wp S.E., Norhidayah M., Ar M.N.A. (2025). Factors associated with multidrug-resistant organism (MDRO) mortality: an analysis from the national surveillance of multidrug-resistant organism, 2018-2022. BMC Infect. Dis..

[2] Alrafaie A. (2025). Harnessing phages to tackle antimicrobial resistance: a Saudi Arabian perspective. Front. Cell. Infect. Microbiol..

[3] Vadaga B.S., Sharma S., Batchu R., Dasgupta M., Kodgire P. (2026). Unveiling the role of outer membrane proteins (OMPs) in biofilm formation and harnessing them for targeting biofilm-forming bacterial infections. World J. Microbiol. Biotechnol..

[4] Mirzaei R., Mohammadzadeh R., Alikhani M.Y., Shokri Moghadam M., Karampoor S., Kazemi S., Barfipoursalar A., Yousefimashouf R. (2020). The biofilm-associated bacterial infections unrelated to indwelling devices. IUBMB Life.

[5] Grooters K.E., Ku J.C., Richter D.M., Krinock M.J., Minor A., Li P., Kim A., Sawyer R., Li Y. (2024). Strategies for combating antibiotic resistance in bacterial biofilms. Front. Cell. Infect. Microbiol..

[6] Burgess A., MacGowan A. (2026). Antibiotic resistance. Medicine.

[7] Farah H., Kadhim-Abosaoda M., Mohaisen-Mousa H., Renuka Jyothi S., Priyadarshini-Nayak P., Bethanney Janney J., Singh G., Singh-Chauhan A., Kumar-Mishra M. (2026). Nanomedicine strategies against biofilm-associated infections: advances, challenges, and translational barriers. Microbiologyopen.

[8] Dolai J., Biswas A., Jana N.R. (2022). Piezoelectric nanoparticles for ultrasound-based wireless therapies. ACS Appl. Nano Mater..

[9] Liang X., Jiang R., Lu Y., Su Y., Zhao Y. (2025). Piezoelectric enables Universality enhancement of conventional water treatment: mechanisms, Effects, and challenges. Chem. Eng. J..

[10] Liu G., Li C., Li D., Xue W., Hua T., Li F. (2024). Application of catalytic technology based on the piezoelectric effect in wastewater purification. J. Colloid Interface Sci..

[11] Sudrajat H., Rossetti I., Carra I., Colmenares J.C. (2024). Piezocatalytic reduction: an emerging research direction with bright prospects. Curr. Opin. Chem. Eng..

[12] Wang X., Dai X., Chen Y. (2023). Sonopiezoelectric nanomedicine and materdicine. Small.

[13] Sengupta D., Naskar S., Mandal D. (2023). Reactive oxygen species for therapeutic application: role of piezoelectric materials. Phys. Chem. Chem. Phys..

[14] Moreira J., Pandur Ž., Fernandes M., Martins P., Correia V., Lanceros-Mendez S., Stopar D. (2025). Insights into the antimicrobial mechanism of piezoelectric materials. ACS Omega.

[15] Yang S., Wang Y., Liang X. (2023). Piezoelectric nanomaterials activated by ultrasound in disease treatment. Journal.

[16] Barik S., Namdeo P.K., Sharma R.K. (2024). Nanomaterials-Based approach for photodynamic therapy. ChemistrySelect.

[17] Dong H., Zhu Z., Li Z., Li M., Chen J. (2024). Piezoelectric composites: State-of-the-Art and future prospects. JOM.

[18] Li S., Tang X., Guo W., Li Y., Chen D., Zhang J., Zhang Q., Xu H., Zhou X., Wan Z., Premadasa R., Lu H., Zhang Q., Salehi H., Jiao P. (2024). Numerical simulations of piezoelectricity and triboelectricity: from materials, structures to devices. Appl. Mater. Today.

[19] L. Liu, G. Zhang, J. Ma, D. Wang, J. Wang, L. Xuan, J. Nie, G. Wang, Y. Wang, Piezo-photocatalysis over high-nuclearity titanium-oxide cluster via lead heterocoordination enhances activity by piezoelectric effect, Sci. Adv. 11 eaea4692, 10.1126/sciadv.aea4692. PMC12710690 41406209

[20] Liu S., Yang B., Huo Y., Zhao J., Hu J., Chen L., Li L., Tan M.-L., Liu P., Cai K., Ji W. (2025). Coordination-Driven assembly modulates the piezoelectric response of bio-inspired amino acid-based supramolecular materials. J. Am. Chem. Soc..

[21] Lu C., Chen X. (2022). Flexible piezoionic strain sensors toward artificial intelligence applications. Synlett.

[22] Pandey R., Kumar Mishra S., Kumar Dubey A. (2024). Recent Advances in smart piezoelectric biomaterials: animal studies and beyond. Chem. Eng. J..

[23] Shang S.Y., Zheng F.Y., Tan W., Xing Z.Y., Chen S.Y., Peng F.L., Lv X., Wang D., Zhu X.D., Wu J.G., Zhou Z.K., Zhang X.D., Yang X. (2025). Piezoelectric biomaterial with advanced design for tissue infection repair. Adv. Sci..

[24] Zhang X.Y., Yang Z.W., Zhang J., Wang L.W., Zhou M., Ren N., Ding L.H., Wang A.Z., Wang Z., Liu H., Yu X. (2024). Piezotronic effect enhanced catalytic sterilization: mechanisms and practical applications. Nano Energy.

[25] Chen X.G., Zhang S.P., Peng S.F., Qian Y., Zhou J.H. (2025). Piezoelectric materials for bone implants: opportunities and challenges. Nano Energy.

[26] Huang H.G., Wang K.Z., Liu X.Y., Liu X., Wang J.Z., Suo M.R., Wang H., Chen S., Chen X., Li Z.H. (2025). Piezoelectric biomaterials for providing electrical stimulation in bone tissue engineering: barium titanate. J. Orthop. Transl..

[27] Zaszczynska A., Zabielski K., Gradys A., Kowalczyk T., Sajkiewicz P. (2024). Piezoelectric scaffolds as smart materials for bone tissue engineering. Polymers.

[28] Zhang J.Y., Shen W., Ge X.M., Yan P., Hu J.R., Wang R. (2025). Piezoelectric materials lead to bone regeneration: from basic research to the exploration of innovation pathways. Int. J. Polym. Mater. Polym. Biomater..

[29] Ren Z., Peng Y., He H., Ding C., Wang J., Wang Z., Zhang Z. (2023). Piezoelectrically mediated reactions: from catalytic reactions to organic transformations. Chin. J. Chem..

[30] Tu S., Guo Y., Zhang Y., Hu C., Zhang T., Ma T., Huang H. (2020). Piezocatalysis and Piezo-Photocatalysis: Catalysts classification and modification strategy, reaction mechanism, and practical application. Adv. Funct. Mater..

[31] Guan L., Mehdi D., Li H., Chen F., Jin S. (2025). Covalent organic frameworks: an emerging class of piezoelectric materials for mechanical energy transfer application. Chin. Chem. Lett..

[32] Wang X.Q., Zhang S.J., Hu Y.S., Zhou W., Huang X.J. (2025). Piezoelectric polymers and their applications in antimicrobial fields. Mater. Chem. Front..

[33] Ghosh B., Ullah Z., Sehar T., Sarkar S. (2025). Piezoelectric sterilization techniques: from innovations to applications. Front. Chem..

[34] Chen C., Zhang Y., Zheng Y., Zhang Y., Liu H., Wu J., Yang L., Yang Z. (2025). Topology in biological piezoelectric materials. Adv. Mater..

[35] Singh K., Verma R., Chauhan A., Jasrotia R., Saini S., Thakur P., Kumar V., Thakur P., Thakur A. (2024). Waste water treatment using piezoelectric materials: a review on piezo-photocatalysis. Top. Catal..

[36] Kumar Das K., Kesarwani U., Prakash R., Maiti P., Shankar O., Dubey A.K. (2025). Piezoelectric catalyst BaTiO3 and K0.5Na0.5NbO3 induced cellular and antibacterial response in poly (vinylidene fluoride) for self-powered implants for orthopedic applications. Catal. Today.

[37] Ren J., Wang X., Bao T., Shen X., Yin D., Liang Q., Sun S., Xiao C., Deng C. (2024). Piezoelectric dual network dressing with adaptive electrical stimulation for diabetic infected wound repair via antibacterial, antioxidant, anti-inflammation, and angiogenesis. Chem. Eng. J..

[38] Zhang S., Huang X., Wang X., Li L. (2025). Smart stimuli-responsive piezoelectric antibacterial adhesives for caries prevention. Int. Dent. J..

[39] Chang J., Maltby T., Moineddini A., Shi D., Wu L., Chen J., Yu J., Hung J., Viola G., Vilches A., Song W. (2025).

[40] Zhang X., Yang Z., Zhang J., Wang L., Zhou M., Ren N., Ding L., Wang A., Wang Z., Liu H., Yu X. (2024). Piezotronic effect enhanced catalytic sterilization: mechanisms and practical applications. Nano Energy.

[41] Linh N.H., Dong N.X., Quang T.T., Hung D.T., Truong D.V. (2025). Revealing photo-electrochemical, piezoelectric, and ferroelectric properties of γ-SnTe monolayer via density functional theory. Comput. Mater. Sci..

[42] Jiang T., Wang Y., Cai C., Nie C., Peng H., Ao Z. (2025). Piezocatalysis for water treatment: mechanisms, recent advances, and future prospects. Environ. Sci. Ecotechnol..

[43] Zhu P., Chen Y., Shi J.L. (2020). Piezocatalytic Tumor therapy by ultrasound-triggered and BaTiO_3_-Mediated piezoelectricity. Adv. Mater..

[44] Chen S., Zhu P., Mao L.J., Wu W.C., Lin H., Xu D.L., Lu X.Y., Shi J.L. (2023). Piezocatalytic medicine: an emerging frontier using piezoelectric materials for biomedical applications. Adv. Mater..

[45] Wang K., Han C., Li J., Qiu J., Sunarso J., Liu S. (2022). The mechanism of piezocatalysis: energy band theory or screening charge effect?. Angew. Chem. Int. Ed..

[46] Bossl F., Menzel V.C., Jeronimo K., Arora A., Zhang Y., Comyn T.P., Cowin P., Kirk C., Robertson N., Tudela I. (2023). Importance of energy band theory and screening charge effect in piezo-electrocatalytical processes. Electrochim. Acta.

[47] Wu W.Z., Wang Z.L. (2016). Piezotronics and piezo-phototronics for adaptive electronics and optoelectronics. Nat. Rev. Mater..

[48] Amaechi I.C., Youssef A.H., Dörfler A., González Y., Katoch R., Ruediger A. (2022). Catalytic applications of non-centrosymmetric oxide nanomaterials. Angew. Chem., Int. Ed..

[49] Zaszczynska A., Gradys A., Sajkiewicz P. (2020). Progress in the applications of smart piezoelectric materials for medical devices. Polymers.

[50] Yang X.B.Q., Yang Z.R., Wang X.S., Guo Y.H., Xie Y.F., Yao W.R., Kawasaki H. (2024). Piezoelectric nanomaterials for antibacterial strategies. Appl. Mater. Today.

[51] Wang Y., Liu K.K., Zhao W.B., Sun J.L., Chen X.X., Zhang L.L., Cao Q., Zhou R., Dong L., Shan C.X. (2023). Antibacterial fabrics based on synergy of piezoelectric effect and physical interaction. Nano Today.

[52] Moreira J., Pandur Z., Fernandes M., Martins P., Correia V., Lanceros-Mendez S., Stopar D. (2025). Insights into the antimicrobial mechanism of piezoelectric materials. ACS Omega.

[53] Liu W., Li J., Chen Z., Liang Z., Yang B., Du K., Fu J., Mahjoub A.R., Xing M. (2024). Unveiling the “sono-physico-chemical” essence: cavitation and vibration effects in ultrasound–assisted processes. Chin. J. Catal..

[54] Chen Y.B., Wan X.F., Yue Y., He S., Cao J., He W.X., Tai T.T., Wang D., Zhou Z.K., Deng Y. (2025). Low-Intensity ultrasound-activated cavitation effect triggers piezoelectric catalysis coordinating respiratory chain interference tactics against bacterial infection. Adv. Funct. Mater..

[55] Yu Y., Zeng Y.X., Ouyang Q.L., Liu X.M., Zheng Y.F., Wu S.L., Tan L. (2023). Ultrasound-Induced abiotic and biotic interfacial electron transfer for efficient treatment of bacterial infection. ACS Nano.

[56] Wang X.H., Shan M.Y., Zhang S.K., Chen X., Liu W.T., Chen J.Z., Liu X.Y. (2022). Stimuli-Responsive antibacterial materials: molecular structures, design principles, and biomedical applications. Adv. Sci..

[57] Zhu Z.X., Gou X., Liu L.Y., Xia T., Wang J.Y., Zhang Y.M., Huang C.J., Zhi W., Wang R., Li X.H., Luo S.N. (2023). Dynamically evolving piezoelectric nanocomposites for antibacterial and repair-promoting applications in infected wound healing. Acta Biomater..

[58] Tian G., Guo L., Gao Y., Deng W., Wang S., Xu T., Peng L., Zhang B., Yang T., Lan B., Sun Y., Ao Y., Huang L., Liu Y., Li X., Jin L., Yang W., Yu X. (2025). A wearable all-in-one obstructive sleep apnea management system with flexible piezoelectric monitoring and soft magnetoelastic stimulating. Matter.

[59] Takagaki K., Takahashi K., Hayashi T., Takeuchi S., Kuwahara N., Ishii Y. (2024). Mask-Type acoustic sensor featuring a conventional disposable mask embedded with electrospun Poly(styrene) fiber mats. Adv. Energy Sustain. Res..

[60] Zang P.Y., Yu C.H., Zhang R., Yang D., Gai S.L., Yang P.P., Lin J. (2024). Revealing the optimization route of piezoelectric sonosensitizers: from mechanism to engineering methods. Small.

[61] Zhang Z., Wang Z., Li X., Zheng Y., Yang Z. (2025). Design and manufacturing of piezoelectric biomaterials for bioelectronics and biomedical applications. Chem. Rev..

[62] Chen C., Yang X., Liu Y., Jia J., Li Y., Dai X., Liu O. (2024). Piezoelectric materials for anti-infective bioapplications. J. Mater. Chem. B.

[63] Meng F., Guo C., Cui T., Xu M., Chen X., Xu H., Liu C., Chen S. (2025). Piezoelectric catalysis for antibacterial applications. Mater. Chem. Front..

[64] Wu C., Cheng K., Cheng J., Chen P., Mu G., Zhao K., Tang Y. (2024). Reinforcing titanium surface modification through the integration of piezoelectric effect and structural design to mitigate early bacterial infection. Ceram. Int..

[65] Pradeesh E.L., Udhayakumar S., Vasundhara M.G., Kalavathi G.K. (2022). A review on piezoelectric energy harvesting. Microsyst. Technol..

[66] Zhao H., Yao Z., Li T., Lu Y., Wang K., He J., Tian S., Huang F., He C. (2025). Phase and defect engineering of 1T/2H MoS2-x nanosheets for enhanced piezocatalytic inactivation of antibiotic-resistant bacteria. J. Hazard Mater..

[67] Liu K., Zhou Z., Wang H., Li Q., Chen B., Wang X., Nie J., Ma G. (2025). A heterojunction piezoelectric antimicrobial Asymmetric Hydrogel for dynamic wound healing and monitoring. Small.

[68] Han M., Yildiz E., Bozuyuk U., Aydin A., Yu Y., Bhargava A., Karaz S., Sitti M. (2024). Janus microparticles-based targeted and spatially-controlled piezoelectric neural stimulation via low-intensity focused ultrasound. Nat. Commun..

[69] Moreira J., Fernandes M.M., Carvalho E.O., Nicolau A., Lazic V., Nedeljković J.M., Lanceros-Mendez S. (2022). Exploring electroactive microenvironments in polymer-based nanocomposites to sensitize bacterial cells to low-dose embedded silver nanoparticles. Acta Biomater..

[70] Sun L., Yang W., Xie S., Xi X., Song A., Li G., Wei J., Zhao J. (2025). Piezoelectric iridium-doped Bismuth Ferrite/Sodium alginate Hydrogel for antibiosis and stimulating osteoblastic differentiation. ACS Appl. Nano Mater..

[71] Jia P., Li J., Huang H. (2024). Piezocatalysts and Piezo-Photocatalysts: from material design to diverse applications. Adv. Funct. Mater..

[72] Tian S.-L., Song L.-N., Chang L.-M., Liu W.-Q., Wang H.-F., Xu J.-J. (2024). A magnetic/force coupling assisted lithium-oxygen battery based on magnetostriction and piezoelectric catalysis of CoFe2O4/BiFeO3 cathode. Nano Energy.

[73] Li Z., Roscow J., Khanbareh H., Haswell G., Bowen C. (2024). Energy harvesting from water flow by using piezoelectric materials. Adv. Energy Sustain. Res..

[74] Ali A., Ali S., Shaukat H., Khalid E., Behram L., Rani H., Altabey W.A., Kouritem S.A., Noori M. (2024). Advancements in piezoelectric wind energy harvesting: a review. Results Eng..

[75] Mhiri M.T., Larbi W., Chouchane M., Guerich M. (2025). Optimization of a macrofiber piezoelectric energy harvester using artificial neural networks. Compos. Struct..

[76] Wang M., Yang Y., Dai E., Rao W.-F. (2022). Fast optimize arm wearable piezoelectric energy harvesters via artificial neural network. Mater. Lett..

[77] Chen X.X., Wang J.X., Wang Z.C., Xu H.W., Liu C., Huo B.J., Meng F.Q., Wang Y.L., Sun C.Y. (2023). Low-frequency mechanical energy in the environment for energy production and piezocatalytic degradation of organic pollutants in water: a review. Journal of Water Process Engineering.

[78] Panda S., Hajra S., Mistewicz K., In-na P., Sahu M., Rajaitha P.M., Kim H.J. (2022). Piezoelectric energy harvesting systems for biomedical applications. Nano Energy.

[79] Clementi G., Cottone F., Di Michele A., Gammaitoni L., Mattarelli M., Perna G., López-Suárez M., Baglio S., Trigona C., Neri I. (2022). Review on innovative piezoelectric materials for mechanical energy harvesting. Journal.

[80] Li J., Liu X., Zhao G., Liu Z., Cai Y., Wang S., Shen C., Hu B., Wang X. (2023). Piezoelectric materials and techniques for environmental pollution remediation. Sci. Total Environ..

[81] Dowarah S., Kalita P., Kumar S. (2024). Progress in mechanical energy harvesting via piezoelectric polymers and biomaterials. Polymer.

[82] Ghosh B., Ullah Z., Sehar T., Sarkar S. (2025).

[83] Liu W., Zhao Q., Jiang R., Ni X., You T., Li C., Deng Y., Xu B., Chen Y., Chen L. (2025). Stereoisomeric engineering mediated zinc metal electrodeposition: critical balance of solvation and adsorption capability. Adv. Powder Mater..

[84] Feng J., Li X., Xu T., Zhang X., Du X. (2024). Photothermal-driven micro/nanomotors: from structural design to potential applications. Acta Biomater..

[85] Ge M., Ruan Z., Zhu Y.-X., Wu W., Yang C., Lin H., Shi J. (2024).

[86] Chen Y., Wan X., Yue Y., He S., Cao J., He W., Toctocan Tai T., Wang D., Zhou Z., Deng Y. (2024). Low‐Intensity Ultrasound‐Activated cavitation effect triggers piezoelectric catalysis coordinating respiratory chain interference tactics against bacterial infection. Adv. Funct. Mater..

[87] Li K., Xu W., Chen Y., Liu X., Shen L., Feng J., Zhao W., Wang W., Wu J., Ma B. (2023). Piezoelectric nanostructured surface for ultrasound‐driven immunoregulation to rescue titanium implant infection. Adv. Funct. Mater..

[88] Tschon M., Codispoti G., Cabras P., Cafarelli A., Trucco D., Vannozzi L., Manferdini C., Carniato M., Cassiolas G., Martini L., Fini M., D'Atri G., Jost C., Fedutik Y., Nessim G.D., Dumont E., Lisignoli G., Ricotti L. (2026). In vivo efficacy of an injectable piezoelectric nanocomposite hydrogel and low-intensity pulsed ultrasound in two preclinical models of osteoarthritis. Biomaterials.

[89] Weng Z., Wei Q., Ye C., Xu Y., Gao J., Zhang W., Liu L., Zhang Y., Hu J., Zhong Q. (2024). Traditional herb (Moxa) modified zinc oxide nanosheets for quick, efficient and high tissue penetration therapy of fungal infection. ACS Nano.

[90] Roy D., Mukherjee B., Deb S., Pandit S.K., Dolai J., Dhang S., Saha S., Banerjee A., Jana N.R. (2026). Piezoelectric film-based bacterial disinfection by mechanical agitation. ACS Appl. Mater. Interfaces.

[91] Poudel P., Sharma S., Ansari M.N.M., Kumar P., Ibrahim S.M., Vaish R., Kumar R., Thomas P. (2022). The bacterial disinfection of water using a galloping piezoelectric wind energy harvester. Energies.

[92] Chen Y., Guan H., Wang X., Wen Y., He Q., Lin R., Yang Z., Wang S., Zhu X., Zhong T. (2024). Implantable and wireless-controlled antibacterial patch for deep abscess eradication and therapeutic efficacy monitoring. Nano Energy.

[93] Ghosh S., Zheng M., He J., Wu Y., Zhang Y., Wang W., Shen J., Yeung K.W.K., Neelakantan P., Xu C., Qiao W. (2025). Electrically-driven drug delivery into deep cutaneous tissue by conductive microneedles for fungal infection eradication and protective immunity. Biomaterials.

[94] Ke Y., Li T., Li J., Pei M., Wang X., Xie W., Zhuang S., Ye X., Li Z., Wang Z., Yang F. (2026). Heartbeat electro-language: exploring piezoelectric technologies for cardiovascular health monitoring. eScience.

[95] Kim M., Doh I., Oh E., Cho Y.-H. (2023). Flexible piezoelectric pressure sensors fabricated from nanocomposites with enhanced dispersion and vapor permeability for precision pulse wave monitoring. ACS Appl. Nano Mater..

[96] Trevino J.E., Mohan S., Salinas A.E., Cueva E., Lozano K. (2021). Piezoelectric properties of PVDF-conjugated polymer nanofibers. J. Appl. Polym. Sci..

[97] Ahbab N., Naz S., Xu T.-B., Zhang S. (2025). A comprehensive review of piezoelectric PVDF polymer fabrications and characteristics. Journal.

[98] Deng J.Z., Sun Q., Wu Z.Y., Wang Y.Y. (2024). Enhanced self-driven flexible piezoelectric nanogenerator sensor based on NaNbO3/P(VDF-TrFE) films for security applications. Surf. Interfaces.

[99] Liu Z.R., Cai M.J., Zhang X.D., Yu X., Wang S., Wan X.Y., Wang Z.L., Li L.L. (2021). Cell-Traction-Triggered On-Demand electrical stimulation for neuron-like differentiation. Adv. Mater..

[100] Yu X., Wang L.W., Zhu Z.L., Han X., Zhang J., Wang A.Z., Ding L.H., Liu J. (2023). Piezoelectric effect modulates nanozyme activity: underlying mechanism and practical application. Small.

[101] Gao X., Liu Y., Li Y., Jin B., Jiang P., Chen X., Wei C., Sheng J., Liu Y.-N., Li J. (2023). Interfaces, Piezoelectric nanozyme for dual-driven catalytic eradication of bacterial biofilms. ACS Appl. Mater. Interfaces.

[102] Zheng J., Liu H., Deng Y., Lian L., Hua N., Zhao S., Chen W., Li J., Liu Y.-N. (2024). Biofilm microenvironment-mediated dual-gases-driven nanomotors for combating drug-resistant bacterial infections. ACS Mater. Lett..

[103] Zhu Z., Gou X., Liu L., Xia T., Wang J., Zhang Y., Huang C., Zhi W., Wang R., Li X. (2023). Dynamically evolving piezoelectric nanocomposites for antibacterial and repair-promoting applications in infected wound healing. Acta Biomater..

[104] Zhao W., Ding Q., Zhou B., Liu J., Shi Y., Liu C., Li C., Dong B., Qi M., Kim J.S. (2024). Nitric oxide‐actuated titanium dioxide janus nanoparticles for enhanced multimodal disruption of infectious biofilms. Adv. Funct. Mater..

[105] Tian B.S., Tian R.X., Liu S.H., Wang Y., Gai S.L., Xie Y., Yang D., He F., Yang P.P., Lin J. (2023). Doping engineering to modulate lattice and electronic structure for enhanced piezocatalytic therapy and ferroptosis. Adv. Mater..

[106] Zhou D., Li Z., Chen Y., Dong H., Zhou Y., Bian Z., Zhu M. (2025). Leveraging solvent effects for enhanced oxidation and coordination in selective piezocatalytic gold recovery from end-of-life electronics. Angew. Chem. Int. Ed..

[107] Yin H., Li X., He A., Dai B., Dong H., Zhou C., Zhang H., Qie W., Wang H., Kong R., Pan Z., Xie Y. (2026). Comprehensive band structure and surface reactivity engineering enable high-performance, enamel-safe dental piezo-photocatalysis. Nano Energy.

[108] Pai S.M., Shah K.A., Sunder S., Albuquerque R.Q., Brütting C., Ruckdäschel H. (2025). Machine learning applied to the design and optimization of polymeric materials: a review. Next Mater..

[109] Yue T., He J., Li Y. (2025). Machine-Learning-Assisted molecular design of innovative polymers. Acc. Mater. Res..

[110] Peng C., Wu W.T., Huo H.X., Li J., Wang E. (2025). Defect-Boosted piezoelectric and nanozymatic synergetic catalysis for deep bacterial abscess therapy. ACS Nano.

[111] Fan Y.Z., Zhai J.X., Wang Z.G., Yin Z.Y., Chen H.Y., Ran M.F., Zhu Z.R., Ma Y.B., Ning C.Y., Yu P., Mao C.B. (2025). Piezoelectric heterojunctions as bacteria-killing bone-regenerative implants. Adv. Mater..

[112] Liu S., Zhang Y., Guo Y., Cheng Z., Yuan M., Xu Z., Liao G., Li Q. (2025). Step-scheme/Mott-Schottky integrated heteroiunctions in BiFeO_3_/ZnIn_2_S_4_/Ag hollow nanospheres: facilitating efficient piezo-photocatalytic activation of peroxydisulfate to enhance nizatidine degradation and antibacterial activity. Colloid Interface Sci..

[113] Peng C., Wu W., Huo H., Li J., Wang E. (2025). Defect-Boosted piezoelectric and nanozymatic synergetic catalysis for deep bacterial abscess therapy. ACS Nano.

[114] Ge M., Zhu W.B., Mei J.W., Hu T.T., Yang C., Lin H., Shi J.L. (2025). Piezoelectric-Enhanced nanocatalysts trigger Neutrophil N1 polarization against bacterial biofilm by disrupting redox homeostasis. Adv. Mater..

[115] Zheng F.Y., Wan X.F., Zhang Y.M., Yue Y., Li Q.C., Zhang Z., Li S.Y., Xu H., Su Q., Chen X.T., Tong L., Zhao L., Cao J., Tang X., Yang X., Wu J.G., Li J., Lv X., Zhou Z.K., Wang D. (2025). A multimodal defect-rich nanoreactor triggers sono-piezoelectric tandem catalysis and iron metabolism disruption for implant infections. Sci. Adv..

[116] Qiu L.H., Ma S.S., Yang R., Zheng D.W., Huang Y.L., Zhu Z.W., Peng S.J., Li M., Zhong H., Peng F. (2025). Ultrasound-activated piezoelectric heterojunction drives nanozyme catalysis to induce bacterial cuproptosis-like death and promote bone vascularization and osseointegration. Biomaterials.

[117] Cheng J., Wu L., Fu H., Hu L., Wang W., Heng B.C., Zhang X., Liu O., Deng X., Liu Y. (2025). Biodegradable piezoelectric janus membrane with enhanced antibacterial and osteoinductive properties for periodontitis therapy. Adv. Healthcare Mater..

[118] Zhao H.N., Yao Z.N., Li T., Lu Y.H., Wang K.W., He J., Tian S.H., Huang F., He C. (2025). Phase and defect engineering of 1T/2H MoS2-x nanosheets for enhanced piezocatalytic inactivation of antibiotic-resistant bacteria. J. Hazard Mater..

[119] Xuan X., Huang S., Qin M., Shen J., Wang L., Zhang X., Zhang J., Lu X., Hou Z., Gao X. (2023). Defective ReS2 triggers high intrinsic piezoelectricity for piezo-photocatalytic efficient sterilization. ACS Appl. Mater. Interfaces.

[120] Shi S.G., Jiang Y.J., Yu Y.X., Liang M.M., Bai Q., Wang L.A., Yang D.Q., Sui N., Zhu Z.L. (2023). Piezo-Augmented and photocatalytic nanozyme integrated microneedles for antibacterial and anti-inflammatory combination therapy. Adv. Funct. Mater..

[121] Yuan R., Xue D., Xu Y., Xue D., Li J. (2022). Machine learning combined with feature engineering to search for BaTiO3 based ceramics with large piezoelectric constant. J. Alloys Compd..

[122] Li X., Qiu J., Cui H., Chen X., Yu J., Zheng K. (2024). Machine learning accelerated discovery of functional MXenes with giant piezoelectric coefficients. ACS Appl. Mater. Interfaces.

[123] Liu Y., He H., Cao Y., Liang Y., Huang J. (2024). Inverse design of TPMS piezoelectric metamaterial based on deep learning. Mech. Mater..

[124] Cao Y., Fu H., Lu J., Chen Y., Jing T., Fan X., Xu B. (2026). Artificial intelligence empowered new materials: discovery, synthesis, prediction to validation. Nano-Micro Lett..

[125] Zhou Y., Ruan Q., Li Y., Fan Y., Chen D. (2025). A full in-silico approach for quantifying the effect of intramolecular hydrogen bonds on physicochemical properties. J. Mol. Liq..

[126] Chen J., Ayranci C., Tang T. (2023). Piezoelectric performance of electrospun PVDF and PVDF composite fibers: a review and machine learning-based analysis. Mater. Today Chem..

[127] Anand A., Kumari P., Kalyani A.K. (2025). High throughput screening of new piezoelectric materials using graph machine learning and knowledge graph approach. Comput. Mater. Sci..

[128] Riebesell J., Goodall R.E.A., Benner P., Chiang Y., Deng B., Ceder G., Asta M., Lee A.A., Jain A., Persson K.A. (2025). A framework to evaluate machine learning crystal stability predictions. Nat. Mach. Intell..

[129] Li H., Lu W., Li C., Liu J., Feng Z., Liu J., Yang L. (2026). Structure-driven prediction and mechanism insights into piezoelectric performance of potassium sodium niobate via interpretable machine learning. Comput. Mater. Sci..

[130] Ma B., Wu X., Zhao C., Lin C., Gao M., Sa B., Sun Z. (2023). An interpretable machine learning strategy for pursuing high piezoelectric coefficients in (K0.5Na0.5)NbO3-based ceramics. npj Comput. Mater..

[131] Sun D., Iqbal N., Liao W., Lu Y., He X., Wang K., Ma B., Zhu Y., Sun K., Sun Z. (2022). Efficient degradation of MB dye by 1D FeWO4 nanomaterials through the synergistic effect of piezo-Fenton catalysis. Ceram. Int..

[132] Feng X., Ma L., Lei J., Ouyang Q., Zeng Y., Luo Y., Zhang X., Song Y., Li G., Tan L. (2022). Piezo-augmented sonosensitizer with strong ultrasound-propelling ability for efficient treatment of osteomyelitis. ACS Nano.

[133] Thakur D., Sharma M., Vaish R., Balakrishnan V. (2021). WS2 monolayer for piezo–phototronic dye degradation and bacterial disinfection. ACS Appl. Nano Mater..

[134] Liu D., Li L., Shi B.-L., Shi B., Li M.-D., Qiu Y., Zhao D., Shen Q.-D., Zhu Z.-Z. (2023). Ultrasound-triggered piezocatalytic composite hydrogels for promoting bacterial-infected wound healing. Bioact. Mater..

[135] Zhao T., Li Y., Zhang Z. (2026). Recent advances in β-phase engineering of PVDF-based piezoelectric composites for enhanced piezoelectricity and wearable applications. Chem. Commun..

[136] Li W.X., Yang T.N., Liu C.S., Huang Y.H., Chen C.X., Pan H., Xie G.Z., Tai H.L., Jiang Y.D., Wu Y.J., Kang Z., Chen L.Q., Su Y.J., Hong Z.J. (2022). Optimizing piezoelectric nanocomposites by high-throughput phase-field simulation and machine learning. Adv. Sci..

[137] Park M.S., Kim M.J., Jeong J.Y., Han D.Y., Kim S., Hwang G.-T., Yoo H., Lee E.K. (2025). A self-powered kinetic motion sensor fabricated from electrospun MOF-5/PVDF-TrFE composites piezoelectric nanogenerators. Macromol. Res..

[138] Ding F., Shuai X., Chen Y., Zhang D., Qi F., Shuai C. (2025). Ti3C2Tx Nanosheet/UIO-66 MOF nanoparticle composites as piezoelectric scaffolds for promoting nerve regeneration. ACS Appl. Nano Mater..

[139] Li B., Lv M., Zhang Y.J., Gong X.Q., Lou Z.Z., Wang Z.Y., Liu Y.Y., Wang P., Cheng H.F., Dai Y., Huang B.B., Zheng Z.K. (2024). Single-Particle imaging photoinduced charge transfer of ferroelectric polarized heterostructures for photocatalysis. ACS Nano.

[140] Guo S., Shu G., Luo H., Kuang X., Zheng L., Wang C., Zhou C.-A., Song L., Ma K., Yue H. (2024). Low-Cytotoxic core–sheath ZnO NWs@ TiO_2–x_ N_y_ triggered piezo-photocatalytic antibacterial activity. ACS Appl. Mater. Interfaces.

[141] Shah A.A., Khan A., Dwivedi S., Musarrat J., Azam A. (2018). Antibacterial and antibiofilm activity of barium titanate nanoparticles. Mater. Lett..

[142] He D., Wang W., Feng N., Zhang Z., Zhou D., Zhang J., Luo H., Li Y., Chen X., Wu J. (2023). Defect-modified nano-BaTiO3 as a sonosensitizer for rapid and high-efficiency sonodynamic sterilization. ACS Appl. Mater. Interfaces.

[143] Seil J.T., Taylor E.N., Webster T.J. (2009). Ieee, reduced activity of Staphylococcus epidermidis in the presence of sonicated piezoelectric zinc oxide nanoparticles. Journal.

[144] Tang Y., Huang Q.-X., Zheng D.-W., Chen Y., Ma L., Huang C., Zhang X.-Z.J.M.T. (2022). Engineered Bdellovibrio bacteriovorus: a countermeasure for biofilm-induced periodontitis. Mater. Today.

[145] Cai X., Li B., Zhang Y., Han J., Han Y. (2024). Piezopotential and vacancies co-enhanced sonodynamic response of a ZnO-TiO_2-x_ heterojunction array for osteointegration in MRSA-infected osteomyelitis. Chem. Eng. J..

[146] Masimukku S., Hu Y.-C., Lin Z.-H., Chan S.-W., Chou T.-M., Wu J.M. (2018). High efficient degradation of dye molecules by PDMS embedded abundant single-layer tungsten disulfide and their antibacterial performance. Nano Energy.

[147] Chen X., Xu H., Liu C., Wang Z., Wang R., Wang J., Pan R., Qi J., Wang Y., Meng F.J.C.E.J. (2024). Synthesis and characterization of Vs-B/MoS_2_ with double defects for efficient piezocatalytic antibiotic degradation and bacterial disinfection. Chem. Eng. J..

[148] Feng X., Lei J., Ma L., Ouyang Q., Zeng Y., Liang H., Lei C., Li G., Tan L., Liu X. (2022). Ultrasonic interfacial engineering of MoS_2_‐modified Zn single‐atom catalysts for efficient osteomyelitis sonodynamic ion therapy. Small.

[149] Jin L., Liu X., Zheng Y., Li Z., Zhang Y., Zhu S., Jiang H., Cui Z., Chu P.K., Wu S. (2022). Interface polarization strengthened microwave catalysis of MoS2/FeS/Rhein for the therapy of bacteria‐infected osteomyelitis. Adv. Funct. Mater..

[150] Jin L., Zheng Y., Liu X., Zhang Y., Li Z., Liang Y., Zhu S., Jiang H., Cui Z., Wu S. (2022). Magnetic composite rapidly treats Staphylococcus aureus‐infected osteomyelitis through microwave strengthened thermal effects and reactive oxygen species. Small.

[151] Xu M., Wu S., Ding L., Lu C., Qian H., Qu J., Chen Y. (2023). Engineering ultrasound-activated piezoelectric hydrogels with antibacterial activity to promote wound healing. J. Mater. Chem. B.

[152] Carvalho E., Marques-Almeida T., Cruz B., Correia D., Esperança J., Irastorza I., Silvan U., Fernandes M., Lanceros-Mendez S., Ribeiro C. (2024). Piezoelectric biomaterials with embedded ionic liquids for improved orthopedic interfaces through osseointegration and antibacterial dual characteristics. Biomater. Adv..

[153] Shuai C., Liu G., Yang Y., Qi F., Peng S., Yang W., He C., Wang G., Qian G. (2020). A strawberry-like Ag-decorated barium titanate enhances piezoelectric and antibacterial activities of polymer scaffold. Nano Energy.

[154] Bai Q., Zhang J., Yu Y., Zhang C., Jiang Y., Yang D., Liu M., Wang L., Du F., Sui N. (2022). Piezoelectric activatable nanozyme-based skin patch for rapid wound disinfection. ACS Appl. Mater. Interfaces.

[155] Li M., Zou X., Ding Y., Wang W., Cheng Z., Wang D., Wang Z., Shao Y., Bai J. (2022). Multifunctional sensors for respiration monitoring and antibacterial activity based on piezoelectric PVDF/BZT-0.5 BCT nanoparticle composite nanofibers. Smart Mater. Struct..

[156] Li Q., Lin R., Tang Z., Liang S., Xue X., Xing L. (2024). A flexible self-cleaning/antibacterial PVDF/T-ZnO fabric based on piezo-photocatalytic coupling effect for smart mask. J. Phys. Appl. Phys..

[157] Guo W., Wang Y., Zhang K., Dai X., Qiao Z., Liu Z., Yu B., Zhao N., Xu F.-J. (2023). Near-infrared light-propelled MOF@ Au nanomotors for enhanced penetration and sonodynamic therapy of bacterial biofilms. Chem. Mater..

[158] Cai L., Du J., Han F., Shi T., Zhang H., Lu Y., Long S., Sun W., Fan J., Peng X. (2023). Piezoelectric metal–organic frameworks based sonosensitizer for enhanced nanozyme catalytic and sonodynamic therapies. ACS Nano.

[159] Guo Y., Mao C., Wu S., Wang C., Zheng Y., Liu X. (2024). Ultrasound-Triggered piezoelectric catalysis of zinc oxide@glucose derived carbon spheres for the treatment of MRSA infected osteomyelitis. Small.

[160] Huang H., Miao Y., Li Y. (2025). Recent advances of piezoelectric materials used in sonodynamic therapy of tumor. Coord. Chem. Rev..

[161] Zhu L.C., Guo Z.S., Luo Y., Huang H.Y., Zhang K.X., Duan B.B., Peng R.M., Yao H.C., Liang C., Wang K.Y. (2025). High-Efficiency carriers' separation strategy based ultrasmall-bandgap CuWO4 sono-enhances GSH antagonism for cuproptosis Cascade immunotherapy. Adv. Sci..

[162] Zhong X.Y., Li X.Y., Gu L.P., Yang H., Du J., Wang Q., Li Y.H., Miao Y.Q. (2025). Piezoelectric-mediated two-dimensional copper-based metal-organic framework for synergistic sonodynamic and cuproptosis-driven tumor therapy. J. Colloid Interface Sci..

[163] Bleher K., Cieslik P.A., Comba P. (2025). Bispidine coordination chemistry. Dalton Trans..

[164] Grover V., Ravikanth M. (2024). Coordination chemistry of porphycenes. Coord. Chem. Rev..

[165] Wang Y., Su P., Lin Z., Li X., Chen K., Ye T., Li Y., Zou Y., Wang W. (2025). A Tribo/Piezoelectric nanogenerator based on Bio-MOFs for energy harvesting and antibacterial wearable device. Adv. Mater..

[166] Gauffre F., Coppel Y., Marty J.-D., Mingotaud C., Kahn M.L. (2025). When coordination chemistry meets soft matter: from hybrid nanoparticles to assemblies. Coord. Chem. Rev..

[167] Yu W., Lu Z., Jiang H., Li A., Chen L. (2025). Metal-organic framework composites for green catalysis: coordination chemistry approaches to sustainable energy and environmental solutions. Coord. Chem. Rev..

[168] Pan Q., Zheng Y., Zhou Y., Zhang X., Yuan M., Guo J., Xu C., Cheng Z., Kheraif A.A.A., Liu M. (2024). Doping engineering of piezo‐sonocatalytic Nanocoating Confer dental implants with enhanced antibacterial performances and osteogenic activity. Adv. Funct. Mater..

[169] Dong H., Zhou Y., Wang L., Chen L., Zhu M. (2024). Oxygen vacancies in piezocatalysis: a critical review. Chem. Eng. J..

[170] Wan L.C., Tian W.R., Li N.J., Chen D.Y., Xu Q.F., Li H., He J.H., Lu J.M. (2022). Hydrophilic porous PVDF membrane embedded with BaTiO_3_ featuring controlled oxygen vacancies for piezocatalytic water cleaning. Nano Energy.

[171] Han Y., Zhang H., Yang R., Yu X., Marfavi Z., Lv Q., Zhang G., Sun K., Yuan C., Tao K. (2024). Ba^2+^-doping introduced piezoelectricity and efficient Ultrasound-Triggered bactericidal activity of brookite TiO_2_ nanorods. J. Colloid Interface Sci..

[172] Lei C., Lei J., Zhang X., Wang H., He Y., Zhang W., Tong B., Yang C., Feng X. (2023). Heterostructured piezocatalytic nanoparticles with enhanced ultrasound response for efficient repair of infectious bone defects. Acta Biomater..

[173] Teichert J., Ruck M. (2019). Influence of common anions on the coordination of metal cations in polyalcohols. Eur. J. Inorg. Chem..

[174] Lei J., Wang C., Feng X., Ma L., Liu X., Luo Y., Tan L., Wu S., Yang C. (2022). Sulfur-regulated defect engineering for enhanced ultrasonic piezocatalytic therapy of bacteria-infected bone defects. Chem. Eng. J..

[175] Hu H., Li X., Zhang K., Yan G., Kong W., Qin A., Ma Y., Li A., Wang K., Huang H., Sun X., Ma T. (2025). Dual modification of metal–organic frameworks for exceptional high piezo-photocatalytic hydrogen production. Adv. Mater..

[176] Liu P., Qin R., Fu G., Zheng N. (2017). Surface coordination chemistry of metal nanomaterials. J. Am. Chem. Soc..

[177] Roy S., Wang S., Ullah Z., Hao H., Xu R., Roy J., Gong T., Hasan I., Jiang W., Li M., Mondal D., Li J., Jin J., Zhang Y., Xia W., Guo B. (2025). Defect-Engineered biomimetic piezoelectric nanocomposites with enhanced ROS production, macrophage Re-polarization, and Ca^2+^ channel activation for therapy of MRSA-Infected wounds and osteomyelitis. Small.

[178] Chowdhury A.R., Jaksik J., Hussain I., Tran P., Danti S., Uddin M.J. (2019). Surface-Modified nanostructured piezoelectric device as a cost-effective transducer for energy and biomedicine. Energy Technol..

[179] Fan Y., Zhai J., Wang Z., Yin Z., Chen H., Ran M., Zhu Z., Ma Y., Ning C., Yu P., Mao C. (2025). Piezoelectric heterojunctions as bacteria-killing bone-regenerative implants. Adv. Mater..

[180] Wu M., Zhang Z., Liu Z., Zhang J., Zhang Y., Ding Y., Huang T., Xiang D., Wang Z., Dai Y. (2021). Piezoelectric nanocomposites for sonodynamic bacterial elimination and wound healing. Nano Today.

[181] Yu Y., Zeng Y., Ouyang Q., Liu X., Zheng Y., Wu S., Tan L. (2023). Ultrasound-induced abiotic and biotic interfacial electron transfer for efficient treatment of bacterial infection. ACS Nano.

[182] Sharma A., Bhardwaj U., Jain D., Kushwaha H.S. (2022). NaNbO3/ZnO piezocatalyst for non-destructive tooth cleaning and antibacterial activity. iScience.

[183] Zheng Y., Wang S., Jin W., Li Z., Yang G., Li X., Li N., Wang Y., Sheng F., Song Z. (2025). An ultrasound-driven PLGA/Zn-KNN hybrid piezoelectric scaffold with direct and immunoregulatory antibacterial activity for bone infection. Bioact. Mater..

[184] Huang H., Wang K., Liu X., Liu X., Wang J., Suo M., Wang H., Chen S., Chen X., Li Z. (2025). Piezoelectric biomaterials for providing electrical stimulation in bone tissue engineering: barium titanate. J. Orthop. Transl..

[185] Mao L., Bai L., Wang X., Chen X., Zhang D., Chen F., Liu C. (2022). Enhanced cell osteogenesis and osteoimmunology regulated by piezoelectric biomaterials with controllable surface potential and charges. ACS Appl. Mater. Interfaces.

[186] Zhang J., Xue D., Tang J., Liu H., Fu S., Liu X., Gu C., Zhou X., Jiang T. (2024). Piezoelectric modulated charge transfer in SERS substrate based on black phosphorous-graphene oxide/polyvinylidene fluoride. Chem. Eng. J..

[187] Yang C., Saiding Q., Chen W., An S., Zhao S., Khan M.M., Kong N., Ge M., Shi J., Lin H., Tao W. (2026). Chemically modified and inactivated bacteria enable intra-biofilm drug delivery and long-term immunity against implant infections. Nat. Biomed. Eng..

[188] Huang Y., Lv B., Zhao C., Yin J., Wang Y., Wang Y., Fu X., Wu T., Wu J., Zhang X. (2023). High‐efficiency reactive oxygen species generation by multiphase and TiO_6_ distortion‐mediated superior piezocatalysis in perovskite ferroelectrics. Adv. Funct. Mater..

[189] Lian W., Zhang P., Che H., Liu B., Ao Y. (2025). Efficiently piezo-catalytic generation of reactive oxygen species on phosphorus-doped BiOCl enhancing micropollutants degradation. Small.

[190] Hwang H.J., Choi Y., Kim S., Lee S.H., Choi S.J., Kwon H.-Y., Kwon D., Park S., Lee H., Ok M.-R., Kim Y.-C., Park B.-I., Han H.-S. (2024). Synergistic integration of electrical stimulation, reactive oxygen species regulation, and pro-angiogenic for accelerated wound healing. Nano Energy.

[191] Liu Y., Xu H.-Y., Li B., Qi S.-Y., Jin L.-G., Shan L.-W., Dong L.-M. (2025). Critical influence of morphology regulation on the piezocatalytic mechanism in SnSe. Langmuir.

[192] Chen Y., Ji Y., Fang J., Wang D., Dong R., Dai B. (2024). Recent advances in efficient piezo-photocatalysis modulated by morphology and structure control. Int. J. Hydrogen Energy.

[193] Yang G., Wang C.R., Wang Y.P., Liu X.Q., Zhang Y., Xu M., Deng H.Z., Wang W.W. (2023). Noncovalent co-assembly of aminoglycoside antibiotics@tannic acid nanoparticles for off-the-shelf treatment of pulmonary and cutaneous infections. Chem. Eng. J..

[194] Zhao L.Y., Song X.X., Ouyang X.L., Zhou J.H., Li J.P., Deng D.W. (2021). Bioinspired virus-like Fe_3_O_4_/Au@C nanovector for programmable drug delivery via hierarchical targeting. ACS Appl. Mater. Interfaces.

[195] Zhao X.M., Cao Y.Q., Hu J., Yue Z.X., Liu X., Deng D.W. (2025). Hybrid spike-facilitated capture and biofilm destruction Co-Enhances ultrasound-mediated bactericidal therapy. ACS Nano.

[196] Radu C.M., Radu C.C., Arbanasi E.M., Hogea T., Murvai V.R., Chis I.A., Zaha D.C. (2024). Exploring the efficacy of novel therapeutic strategies for periodontitis: a literature review. Life-Basel.

[197] Woo H.N., Cho Y.J., Tarafder S., Lee C.H. (2021). The recent advances in scaffolds for integrated periodontal regeneration. Bioact. Mater..

[198] Wu S.Y., Luo S.L., Cen Z.H., Li Q.Q., Li L.W., Li W.R., Huang Z.K., He W.Y., Liang G.B., Wu D.C., Zhou M.H., Li Y. (2024). All-in-one porous membrane enables full protection in guided bone regeneration. Nat. Commun..

[199] Li K., Song J., Lu Y.H., Zhang D.X., Wang Y.Q., Wang X.Y., Tang Y.J., Yu Y.J., Zhang X.H., Yang X.P., Cai Q. (2025). Biodegradable piezoelectric janus membrane enabling dual antibacterial and osteogenic functions for periodontitis therapy. ACS Appl. Mater. Interfaces.

[200] Ruan Z., Shi T., Guo Z., Zhu Y., Wang W., Ma Y., Ding C., Zhang Y., Wang X., Chen Y., Lin H., Ge M. (2025). Oxygen-Immunomodulated nanocatalysts enhance anti-infection by trained immunity. Adv. Funct. Mater..

[201] Ma G.R., Cheng K., Wang X., Zeng Y.Q., Hu C.L., He L.Y., Shi Z., Lin H.W., Zhang T., Sun S., Huang P.T. (2025). Dual oxygen supply system of carbon dot-loaded microbubbles with acoustic cavitation for enhanced sonodynamic therapy in diabetic wound healing. Biomaterials.

[202] Zhou Y., Pan Q., Wang R., Ge Y., Zhang X., Guo Y., Sun R., Liu X., Liu S., Liu M. (2025). Red blood cell-mimetic hollow covalent organic framework with oxygen-replenishing and cavitation-amplified for enhanced sonodynamic therapy of periodontitis. Chem. Eng. J..

[203] Fan C.-H., Wu N., Yeh C.-K. (2023). Enhanced sonodynamic therapy by carbon dots-shelled microbubbles with focused ultrasound. Ultrason. Sonochem..

[204] Beguin E., Sheng J., Nesbitt H., Owen J., McHale A., Callan J., Stride E.P. (2016). Magnetic targeting of oxygen loaded microbubbles for sonodynamic therapy. J. Acoust. Soc. Am..

[205] Sun D., Pang X., Cheng Y., Ming J., Xiang S., Zhang C., Lv P., Chu C., Chen X., Liu G. (2020). Ultrasound-switchable nanozyme augments sonodynamic therapy against multidrug-resistant bacterial infection. ACS Nano.

[206] Lai Y.-H., Barman S.R., Ganguly A., Pal A., Yu J.-H., Chou S.-H., Huang E.-W., Lin Z.-H., Chen S.-Y. (2023). Oxygen-producing composite dressing activated by photothermal and piezoelectric effects for accelerated healing of infected wounds. Chem. Eng. J..

[207] Huang X., Cheng S., Gong F., Yang X., Pei Z., Cui X., Hou G., Yang N., Han Z., Chen Y., Cheng Y., Cheng L. (2024). A closed-loop patch based on bioinspired infection sensor for wound management. Nano Today.

[208] Ge M., Guo Z., Ruan Z., Ma Y., Zhang Z., Dong H., Shi T., Hu T., Lu L., Chen Y., Lin H., Tan C. (2025). Chemical substrate-enabled piezoelectric metabolic reprogramming therapy unlocks the vicious triad of diabetic wounds. Cell Biomaterials.

[209] Zhao Y., Liu Y., Liao R., Ran P., Liu Y., Li Z., Shao J., Zhao L. (2024). Biofilm microenvironment-sensitive piezoelectric nanomotors for enhanced penetration and ROS/NO synergistic bacterial elimination. ACS Appl. Mater. Interfaces.

[210] Ding T.X., Liu F.W., Xin H., Chen Y.C., Kong L., Han J., Ma D.Y., Han Y., Zhang L. (2024). Pyro-piezoelectric effect of BaTiO_3_ bio-nanocarrier for osteomyelitis therapy. Nano Today.

[211] Dai B.Y., Xie C., Zhang S.T., Li X.Y., He A., Mou Y.B., Dong H., Zhuang R.H., Yin H., Zhang H., Qie W.X., Wang L., Xie Y.N., Lin Z.Q. (2025). Synergistic photodynamic and piezocatalytic therapies for enhanced infected wound healing via piezo-phototronic effect. Adv. Funct. Mater..

[212] Liu X.Y., Xu W.X., Feng J.K., Wang Y., Li K., Chen Y., Wang W.J., Zhao W.W., Ge S.H., Li J.H. (2025). Adoptive cell transfer of piezo-activated macrophage rescues immunosuppressed rodents from life-threating bacterial infections. Nat. Commun..

[213] Qian X., Lu T.L., Huang C.Q., Zheng D.W., Gong G.C., Chu X., Wang X.L., Lai H.H., Ma L.M., Jiang L., Sun X.D., Ji X.F., Li M., Zhang Y. (2024). Bioinspired sonodynamic nano spray accelerates infected wound healing via targeting and disturbing bacterial metabolism. Adv. Funct. Mater..

[214] Chen Y., Wang C., Zhang Z., Yu F., Wang Y., Ding J., Zhao Z., Liu Y. (2024). 3D-printed piezocatalytic hydrogels for effective antibacterial treatment of infected wounds. Int. J. Biol. Macromol..

[215] Zhang Y., An Q., Zhang S., Ma Z., Hu X., Feng M., Zhang Y., Zhao Y. (2022). A healing promoting wound dressing with tailor-made antibacterial potency employing piezocatalytic processes in multi-functional nanocomposites. Nanoscale.

[216] Kumar A., Sharma M., Vaish R. (2024). WS2 nanoparticles screen printed cotton for dual piezo-and photocatalytic antibacterial and dye degradation performance. Ind. Crops Prod..

[217] Wang C., Sun W., Xiang Y., Wu S., Zheng Y., Zhang Y., Shen J., Yang L., Liang C., Liu X. (2023). Ultrasound‐Activated piezoelectric MoS2 enhances sonodynamic for bacterial killing. Small Sci..

[218] Huang D., Nie M., Wang J., Zhao Y., Sun L. (2024). Spatiotemporal piezoelectric microcapsules for programmable sonodynamic sterilization and wound healing. Chem. Eng. J..

[219] Wang A., Ma X., Yang Y., Shi G., Han L., Hu X., Shi R., Yan J., Guo Q., Zhao Y. (2024). Biophysical-driven piezoelectric and aligned nanofibrous scaffold promotes bone regeneration by re-establishing physiological electrical microenvironment. Nano Res..

[220] Shuai C., Long X., Yang Y., Sun B., Zhang Z., Wang G., Peng S. (2024). Poly (L-lactic acid)-BiFeO_3_/Ti_3_C_2_ scaffolds for antibacterial sonodynamic therapy. ACS Appl. Nano Mater..

[221] He J., Cui S., Hou Y., Liu S., Zhang Z., Zhao M., He L., Wang R., Liu S. (2023). Bifunctional defect mediated direct Z-scheme g-C_3_N_4−x_/Bi_2_O_3−y_ heterostructures with enhanced piezo-photocatalytic properties for efficient tooth whitening and biofilm eradication. J. Mater. Chem. B.

[222] Liu X., Shen L., Xu W., Kang W., Yang D., Li J., Ge S., Liu H. (2021). Low frequency hydromechanics-driven generation of superoxide radicals via optimized piezotronic effect for water disinfection. Nano Energy.

[223] Montoya C., Jain A., Londoño J.J., Correa S., Lelkes P.I., Melo M.A., Orrego S. (2021). Multifunctional dental composite with piezoelectric nanofillers for combined antibacterial and mineralization effects. ACS Appl. Mater. Interfaces.

[224] Dai J.J., Shao J., Zhang Y., Hang R.Y., Yao X.H., Bai L., Hang R.Q. (2024). Piezoelectric dressings for advanced wound healing. J. Mater. Chem. B.

[225] Xu N., Gao Y., Li Z., Chen Y., Liu M., Jia J., Zeng R., Luo G., Li J., Yu Y. (2023). Immunoregulatory hydrogel decorated with Tannic acid/Ferric ion accelerates diabetic wound healing via regulating Macrophage polarization. Chem. Eng. J..

[226] Yu Y., Li P., Zhu C., Ning N., Zhang S., Vancso G.J. (2019). Multifunctional and recyclable photothermally responsive cryogels as efficient platforms for wound healing. Adv. Funct. Mater..

[227] Mao W., Li X., He A., Ding M., Zhang Y., Dai Z., Li Q., Xiu W., Hu Y., Mou Y., Yang D., Dong H. (2026). Harnessing piezoelectric biomaterials for pathogenic eradication and tissue regeneration. Exploration.

[228] Liu Q., Liu X., Fan L., Bai X., Pan H., Luo H., Zhang D., Huang H., Bowen C.R. (2024). Ferroelectric catalytic BaTiO_3_‐based composite insoles to promote healing of infected wounds: analysis of antibacterial efficacy and angiogenesis. Interdiscip. Mater..

[229] Shi J., Ju K., Chen H., Mirabolghasemi A., Akhtar S., Sasmito A., Akbarzadeh A. (2024). 3D printed architected shell-based ferroelectric metamaterials with programmable piezoelectric and pyroelectric properties. Nano Energy.

[230] Liu Q., Song S., Ni Y., Yao M., Li M., Si X. (2026). Discovery of a novel FabI inhibitor as antibacterial agents based on machine learning. Journal of the Chinese Chemical Society n/a.

[231] Jiang Z., Feng J., Wang F., Wang J., Wang N., Zhang M., Hsieh C.-Y., Hou T., Cui W., Ma L. (2025). AI-Guided design of antimicrobial peptide hydrogels for precise treatment of drug-resistant bacterial infections. Adv. Mater..

[232] Moreno-Mateo F., Perea S.H., Onel K.B. (2021). Chronic recurrent multifocal osteomyelitis: diagnosis and treatment. Curr. Opin. Pediatr..

[233] Urish K.L., Cassat J.E. (2020). Staphylococcus aureus osteomyelitis: bone, bugs, and surgery. Infect. Immun..

[234] Huang Y.L., Wan X.F., Su Q., Zhao C.L., Cao J., Yue Y., Li S.Y., Chen X.T., Yin J., Deng Y., Zhang X.Z., Wu T.M., Zhou Z.K., Wang D. (2024). Ultrasound-activated piezo-hot carriers trigger tandem catalysis coordinating cuproptosis-like bacterial death against implant infections. Nat. Commun..

[235] Li J.F., Liu X.M., Zheng Y.F., Cui Z.D., Jiang H., Li Z.Y., Zhu S.L., Wu S.L. (2023). Achieving fast charge separation by ferroelectric ultrasonic interfacial engineering for rapid sonotherapy of bacteria-infected osteomyelitis. Adv. Mater..

[236] Montoya C., Jain A., Londoño J.J., Correa S., Lelkes P.I., Melo M.A., Orrego S. (2021). Multifunctional dental composite with piezoelectric nanofillers for combined antibacterial and mineralization effects. ACS Appl. Mater. Interfaces.

[237] Xu W., Yu Y., Li K., Shen L., Liu X., Chen Y., Feng J., Wang W., Zhao W., Shao J. (2023). Surface-confined piezocatalysis inspired by ROS generation of mitochondria respiratory chain for ultrasound-driven noninvasive elimination of implant infection. J. Mater. Chem. B.

[238] Badaraev A.D., Koniaeva A., Krikova S.A., Shesterikov E.V., Bolbasov E.N., Nemoykina A.L., Bouznik V.M., Stankevich K.S., Zhukov Y.M., Mishin I.P., Varakuta E.Y., Tverdokhlebov S.I. (2020). Piezoelectric polymer membranes with thin antibacterial coating for the regeneration of oral mucosa. Appl. Surf. Sci..

[239] Zhao X., Wang L.-Y., Tang C.-Y., Li K., Huang Y.-H., Duan Y.-R., Zhang S.-T., Ke K., Su B.-H., Yang W. (2023). Electro-microenvironment modulated inhibition of endogenous biofilms by piezo implants for ultrasound-localized intestinal perforation disinfection. Biomaterials.

[240] Liu X., Xu W., Feng J., Wang Y., Li K., Chen Y., Wang W., Zhao W., Ge S., Li J. (2025). Adoptive cell transfer of piezo-activated macrophage rescues immunosuppressed rodents from life-threating bacterial infections. Nat. Commun..

[241] Zhou Y., Zhu X., Qu K., Xu F. (2026). AI-enabled wearable microfluidics for next-generation infection monitoring and therapeutics. Lab Chip.

